# A Genome-Wide Screen Indicates Correlation between Differentiation and Expression of Metabolism Related Genes

**DOI:** 10.1371/journal.pone.0063670

**Published:** 2013-05-22

**Authors:** Priti Roy, Brijesh Kumar, Akhilesh Shende, Anupama Singh, Anil Meena, Ritika Ghosal, Madhav Ranganathan, Amitabha Bandyopadhyay

**Affiliations:** 1 Department of Biological Sciences and Bioengineering, Indian Institute of Technology Kanpur, Kanpur, U.P., India; 2 Department of Chemistry, Indian Institute of Technology Kanpur, Kanpur, U.P., India; Karlsruhe Institute of Technology, Germany

## Abstract

Differentiated tissues may be considered as materials with distinct properties. The differentiation program of a given tissue ensures that it acquires material properties commensurate with its function. It may be hypothesized that some of these properties are acquired through production of tissue-specific metabolites synthesized by metabolic enzymes. To establish correlation between metabolism and organogenesis we have carried out a genome-wide expression study of metabolism related genes by RNA in-situ hybridization. 23% of the metabolism related genes studied are expressed in a tissue-restricted but not tissue-exclusive manner. We have conducted the screen on whole mount chicken (*Gallus gallus*) embryos from four distinct developmental stages to correlate dynamic changes in expression patterns of metabolic enzymes with spatio-temporally unique developmental events. Our data strongly suggests that unique combinations of metabolism related genes, and not specific metabolic pathways, are upregulated during differentiation. Further, expression of metabolism related genes in well established signaling centers that regulate different aspects of morphogenesis indicates developmental roles of some of the metabolism related genes. The database of tissue-restricted expression patterns of metabolism related genes, generated in this study, should serve as a resource for systematic identification of these genes with tissue-specific functions during development. Finally, comprehensive understanding of differentiation is not possible unless the downstream genes of a differentiation cascade are identified. We propose, metabolic enzymes constitute a significant portion of these downstream target genes. Thus our study should help elucidate different aspects of tissue differentiation.

## Introduction

Metabolic enzymes have been extensively studied *in vitro* to delineate their biochemical properties. An implicit assumption that most of these enzymes carry out essential housekeeping functions for the maintenance of cellular or organismal physiology has largely precluded the study of *in vivo* roles of this class of genes. However, several lines of arguments suggest that metabolic enzymes should have tissue-specific roles during embryonic development.

Organs and their constituent tissues have characteristic chemical and physical properties which are commensurate with their functions. Most of these properties are imparted by lipid and carbohydrate macromolecules, by themselves or as conjugates of proteins, synthesized by these tissues. Thus, one may expect that at least some of the enzymes, involved in lipid and carbohydrate metabolism, should have tissue-restricted functions, and synthesis of these enzymes should be a necessary prerequisite for bringing about the phenotypic changes associated with differentiation. Also, enzymes involved in critical post-translational modifications of signaling molecules and/or transcription factors should also be expressed in a cell-type specific manner.

Recent literature suggests that many metabolic enzymes carry out specific cellular functions during embryonic development (Table S1). One of the best known examples of a metabolic enzyme regulating a developmental event is Lunatic Fringe, a glycosyltransferase gene known to be essential for boundary formation in flies as well as in mice [1]. Similarly *Pipe*, a dorso-ventral patterning regulator identified in flies [2] and *Jaws*, a synovial joint positioning regulator identified in mice [3] are metabolic enzymes. Other metabolic enzymes like transglutaminase (TGM2) [4], WW domain containing E3 ubiquitin protein ligase 2 (WWP2) [5], and tyrosine hydroxylase (TH) [6] are known to regulate chondrogenesis, craniofacial development, and the development of heart chambers, respectively. On the other hand, acyl-CoA synthetase (ACSL) was found to function as a gap gene regulating segmentation in Drosophila [7]. Few of these genes were originally identified as developmentally important and later were found out to be metabolic enzymes. On the other hand, some of these metabolic enzymes were originally characterized for their biochemical activity and only later their importance as regulators of development was appreciated. Thus, identification of metabolic enzymes that are potentially important for development, by virtue of their differential expression, may pave the way of discovering their developmental roles, if any. Based on the above we propose that the developing embryo is an attractive biological context for discovering novel tissue-specific roles of metabolic enzymes.

Proteins possessing more than one independent function are collectively termed as “moonlighting proteins”. One of the best known classes of proteins exhibiting moonlighting activity are lens crystallins which are metabolic enzymes [8]. Many metabolic enzymes including some involved in glycolysis and TCA cycle are also known to have moonlighting activity [9], [10]. Many metabolic enzymes including Glyceraldehyde-3-phosphate dehydrogenase (GAPDH), inosine monophosphate dehydrogenase (IMPDH) etc., are known to play roles in transcription[11]–[13]. The serendipitous discovery of atypical roles of metabolic enzymes thus underscores the necessity of creating a resource for systematic identification of candidate metabolic enzymes possessing non-canonical roles. Several proteomic [14] and bioinformatic [15] approaches were employed to discover proteins with moonlighting properties but with limited success.

Thus far, the lack of a suitable resource has limited our ability to systematically investigate tissue-specific roles of metabolism related genes. Genes expected to have tissue-specific functions must be expressed in a tissue-restricted manner. Here we report a study in which we carried out a genome-wide whole-mount mRNA expression screen for metabolism related genes in the developing chick embryo to identify genes expressed in a tissue-restricted manner. Of the 1620 genes investigated, we found that 410 exhibit tissue-restricted expression. Our data reveals striking correlation between differentiation and expression of metabolism related genes. The database of expression patterns compiled through this study (http://202.3.77.85/metab_new2/) should also serve as a valuable resource for systematic identification of candidate metabolism related genes likely to have tissue-restricted functions during organogenesis.

## Materials and Methods

### Tissue

Fertilized white leghorn chicken (*Gallus gallus*) eggs were procured from Government Poultry Farm, Chak Gazaria, U.P., Lucknow, CSA University of Agriculture & Technology, Kanpur and Santosh’ Poultry Farm, Nankari village, Kanpur. The eggs were incubated in a humidified chamber at 38°C for different durations to get desired stage of development (69 Hrs. for HH18, 3.5 days for HH22, 5 days for HH26 and 6 days for HH28). Embryos were harvested and fixed overnight in 4% paraformaldehyde (Sigma). For WM-ISH, the tissues were dehydrated through methanol (Merck) gradient and stored in 100% methanol at −20°C till further use. For section *in situ* hybridiazation (S-ISH), the tissues were dehydrated through ethanol (Merck) gradient, treated with xylene followed by 50% (v/v) xylene/paraffin wax at 55°C for 30 minutes, followed by 10 hours in paraffin wax at 55°C, embedded in paraffin and stored at 4°C till further use.

### *In situ* Hybridizations

Whole-mount *in situ* hybridizations (WM-ISH) were carried out as previously described [16] with minor modifications. All WM-ISH were carried out in 12-well plates (CLS3512, Sigma-Aldrich) containing Net-well inserts (CLS3477, Sigma-Aldrich) and holders (CLS320, Sigma-Aldrich). For each gene, two embryos each for HH18, HH22, HH26 and HH28 were used. An expression pattern was recorded if the signal was identical in both the embryos of the same stage. WM-ISH was repeated for 20% of the genes for which signal was obtained in the first round. For section *in situ* hybridization 8 µm sections from paraffin embedded embryos were collected using LeicaRM2255 microtome. Section *in situ* hybridizations for chromogenic detection were performed as described previously [17]. DIG-labeled probes (single detection) for whole-mount as well as section *in situ* hybridizations were detected with NBT and BCIP (Roche). Fluorescent double RNA in-situ hybridizations were performed using the Tyramide amplification kit (Invitrogen-T30955) according to manufacturer’s protocol. Templates for anti-sense RNA probe synthesis were generated by polymerase chain reaction with T3 and T7 primers on ChEST clones (MRC Geneservice, now known as Source Bioscience). All probes were synthesized with T3 RNA polymerase (Promega) and digoxigenin or biotin labeled nucleotides (Roche). The details of the ChEST clones used as well as the full names of the genes are provided in Table S6. The ChEST clones were streaked on LB agar plates. Four colonies per clone were used for a colony PCR based screening to ensure that no cross-well contamination has occurred. One colony per clone was then used for culture and plasmid DNA isolation.

### Alcian Blue Staining

The paraffin embedded tissue sections were deparaffinized by xylene treatment followed by rehydration to distilled water by passing through ethanol gradient (100% ethanol X 2, 90% ethanol, 70% ethanol, distilled water, 2 minutes each). The slides were then incubated in 3% acetic acid for 3 minutes, followed by 45 minutes staining in 1% solution of alcian blue, 8GX (Sigma, A5268) in 3% acetic acid. The slides were washed under running tap water for 2 minutes and dehydrated to 100% ethanol by passing through ethanol gradient (70% ethanol, 95% ethanol, 100% ethanol, 2 minutes each). After clearing in xylene, the slides were mounted in DPX mountant.

### Imaging

Stained whole embryos were viewed with Leica DMS6D and images were captured with Leica DFC290. For sections Leica DFC 500 was used to capture images.

### Tree Structure Analysis to Determine Relative Correlation between Tissues

In our study we have examined the expression of each gene in 4 different embryonic stages. Thus, a given gene in a given structure may be expressed in 2^4^ = 16 possible unique stage-wise expression patterns (SWEPs). This can be expressed as a sequence of 4 binary digits. For example, a gene that is expressed in stages HH26 and HH28 but not in stages HH18 and HH22 may be represented by 0011. Out of the 16 possible SWEPs, a negligible number of genes had a recurrent expression pattern (ex 1101, 1001). The missing expression in the intermediate stages is likely due to error in experiment or annotation. In all analysis, such patterns are “filled-in” for the missing intermediate stages i.e. a 1101 SWEP is replaced with a 1111 SWEP. This post-processing of the data along with absence of 0000 SWEP, by design of the experiment, reduced the number of possible SWEPs from 16 to 10. On the basis of number of genes with identical SWEPs between different pairs of structures, we have come up with a distance measure between two structures
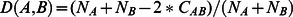
where:

*D*(*A*, *B*) = Distance between structures A and B

*N_A_* = No. of genes expressed in structure A

*N_B_* = No. of genes expressed in structure B

*C_AB_* = No. of genes with identical SWEP common between structures A and B

Since *C_AB_* ≤*Min*(*N_A_*,*N_B_*), 0≤*D*(*A*, *B*)≤1.

*D*(*A*, *B*) = 0 only if *N_A_* = *N_B_* = *C_AB_*, and *D*(*A*, *B*) = 1 only if *C_AB_ = *0. The distance matrix was constructed for nine different Embryonic Structures.

Using this distance measure, a phylogenetic tree of structures was constructed where the most similar structures are closest to each other. We used the Neighbor Joining (NJ) method [18] for this analysis. Seqneighjoin function in MATALB® (Mathworks, Natick, MA, USA) was used to construct the phylogenetic tree.

### Data Access

The compendium of expression patterns for metabolism related genes during chicken embryonic development, that has been generated through this work is archived in an online database. It has several features to identify co-expression groups as well as to find embryonic structures expressing similar sets of metabolic enzymes. It is available at the following website: http://202.3.77.85/metab_new2/signin.php.

## Results

### Metabolism Related Genes Exhibit Tissue-specific Expression

Existing literature [19] and conventional wisdom suggest that the primary objective of a differentiation program should be directed at imparting tissue-specific biochemical and biophysical properties. The objective of this work was to establish a correlation between metabolic activity acquisition and tissue morphogenesis. Expression of mRNAs encoding metabolism related genes may be used as an indicator of acquisition of metabolic activity. For this purpose, we carried out whole mount RNA *in situ* hybridization-based screen at four distinct stages of early chick embryonic development (Hamburger*–*Hamilton stages 18, 22, 26 and 28 or HH18, HH22, HH26 and HH28) [20] encompassing early morphogenetic events. For probe synthesis we acquired 53760 chick expressed sequence tags (ChESTs) available with MRC Gene Service (now known as Source Bioscience) which would provide ∼2.8 fold coverage of all the genes encoded by chick genome, assuming that the chick genome encodes approximately 19,000 genes [21], [22]. To generate a comprehensive picture we aimed to investigate the expression patterns of all metabolism related genes hence we included metabolic enzymes (catalysts of biochemical reactions) as well as transporter molecules (metabolic flux controllers) in our list, thus, in the rest of this report we will use the term “metabolism related genes (MRGs)” to describe our target gene collection. For generating the list of genes to be investigated we used certain filtering criteria as described in Table S2. We finally had 2236 MRGs in our list. In the absence of a fully annotated genome, estimating the accurate number of chick genes involved in metabolism is difficult. However, assuming that the MRC ChEST clone collection contains all chick genes our target list (Table S2) is nearly exhaustive. We have examined the expression of 1620 genes i.e., ∼73%, of our target gene list.

**Figure 1:**
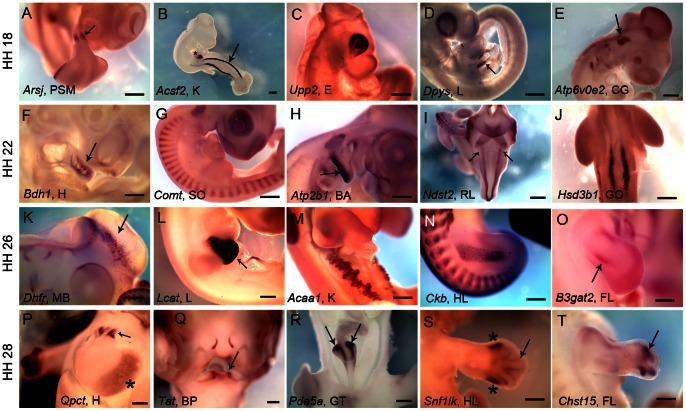
Diversity of expression patterns of metabolism related genes. (A–T) Whole-mount RNA in situ hybridization showing expression patterns of various mRNAs encoding MRGs. (A) *Arsj* is expressed in the pre-somitic mesoderm (arrow) at HH18. (B) *Acsf2* is expressed in the developing kidney (arrow) at HH18. Also, expression is detectable in the developing liver (asterisk). (C) *Upp2* is expressed in the developing eye at HH18. (D) *Dpys* is expressed in the developing liver (arrow) at HH18. (E) *Atp6v0e2* is expressed in the developing cranial ganglia (arrow) at HH18. (F) *Bdh1* is expressed in the developing heart (arrow) at HH22. (G) *Comt* is expressed in the somites at HH22. (H) *Atp2b1* is expressed in the branchial arches (arrow) at HH22. (I) *Ndst2* is expressed in the rhombic lip (arrows) at HH22. (J) *Hsd3b1* is expressed in the developing gonads at HH22. (K) *Dhfr* is expressed in the mid-brain (arrow) at HH26. (L) *Lcat* is expressed in the developing liver (arrow) at HH26. (M) *Acaa1* is expressed in the developing kidney at HH26. (N) *Ckb* is expressed in the hind limb muscles as well as in the somites at HH26. (O) *B3gat2* is expressed in an uncharacterized pattern (arrow) of fore limb at HH26. (P) *Qpct* is expressed in the developing heart ventricle (asterisk) and specifically in the heart valve (arrow) at HH28. (Q) *Tat* is expressed in the beak primordia (arrow) at HH28. (R) *Pde5a* is expressed in the developing ceca horns (arrows) of gut tube at HH28. (S) *Snf1lk* is expressed in the perichondrium (arrow) and in an uncharacterized pattern (asterisks) in the hind limb at HH28. (T) *Chst15* is expressed in an uncharacterized pattern (arrow) in the fore limb at HH28. Scale bar 5 mm. Abbreviations: PSM – pre-somitic mesoderm, K – kidney, E – eye, L – liver, CG – cranial ganglia, H – heart, SO – somites, BA – branchial arches, RL – rhombic lip, GO – gonad, MB – mid-brain, HL – hind limb, BP – beak primordial, GT – gut tube, FL – fore limb, HH- Hamburger and Hamilton stages Full gene names are presented in Table S6.

Of the 1620 genes investigated, expression could be detected for 442 genes. Among these genes, 410 (∼25%) are expressed in a tissue-restricted manner while 32 (2%) genes are ubiquitously expressed (Table 1). In our screen we found considerable diversity in expression patterns of MRGs (Fig. 1). Some of the subcategories of metabolic enzymes included in our screen such as kinases, enzymes involved in SUMOylation, uniquitinylation or GTP hydrolysis have well-established roles in regulating gene expression and thus may be expected to have tissue-restricted expression. Inclusion of genes from these classes could have resulted in identification of disproportionately large number of genes with tissue-restricted expression. However, our analysis (Table 1) shows that presence of genes from these classes did not influence the fraction of genes for which tissue-restricted expression could be detected. Compared to other gene classes, a greater fraction of genes encoding transporters and carrier proteins exhibited tissue-restricted expression–an observation that was expected, based on a previous study [23].

**Table 1:** Summary of the expression screen.

Gene Class/Category	Total number of genesinvestigated	Number of genes showingtissue-restrictedexpression	Percentage	Number of genesshowing ubiquitousexpression
Kinases	215	51	23.7	4
SUMO associated	5	1	20	1
Ubiquitin associated	42	5	11.9	3
Carriers/Transporters	127	42	33	2
GTPase and associated	41	15	36.6	1
Others	1190	296	24.9	21
**TOTAL**	**1620**	**410**	**25.3**	**32**

Summary of the results of whole-mount RNA in situ hybridization for genes from different categories of “metabolism related genes”.

### Most Metabolism Related Genes are Expressed in Multiple Embryonic Structures

**Figure 2:**
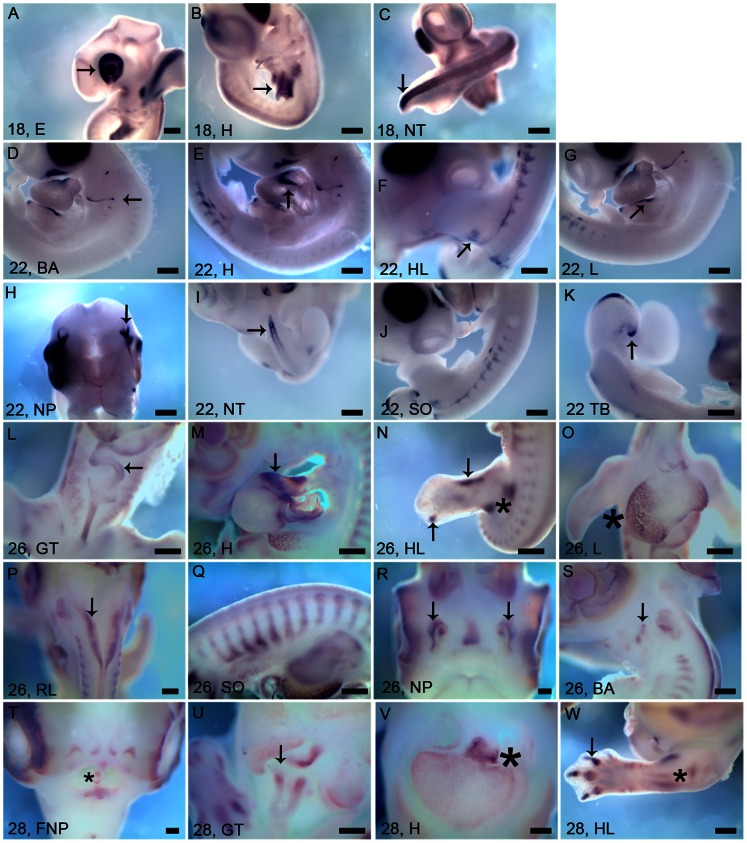
Diverse spatio-temporal expression patterns of lactate dehydrogenase A. (A–W) Whole-mount RNA in situ hybridization showing expression patterns of *Ldha* transcript (A–C) at stage HH18, (A) Eye (arrow), (B) Heart (arrow), (C) Neural tube (arrow), (D–K) at HH22, (D) Branchial arches (arrow), (E) Heart (arrow), (F) Hind limb (arrow), (G) Liver (arrow), (H) Nasal primordial (arrow), (I) Neural tube (arrow), (J) Somites, (K) Tail bud (arrow), (L–S) at HH26, (L) Gut tube (arrow), (M) Heart (arrow), (N) Skeletal elements (arrows) and uncharacterized structures (asterisk), (O) Liver (asterisk), (P) Rhomlic lip (arrow), (Q) Somites, (R) Nasal primordial (arrows), (S) Branchial arches (arrow), (T–W) at HH28, (T) Several structures of frontonasal primordia and tongue (asterisk), (U) Gut tube (arrow), (V) Heart (asterisk), (W) Hind limb digits (arrow) and muscles (asterisk). Scale bar 5 mm. Abbreviations: NT – neural tube, NP – nasal primordial, TB – tail bud, E – eye, L – liver, H – heart, SO – somites, BA – branchial arches, RL – rhombic lip, HL – hind limb, BP – beak primordial, GT – gut tube, FNP – frontonasal primordial. Full gene names are presented in Table S6.

**Figure 3:**
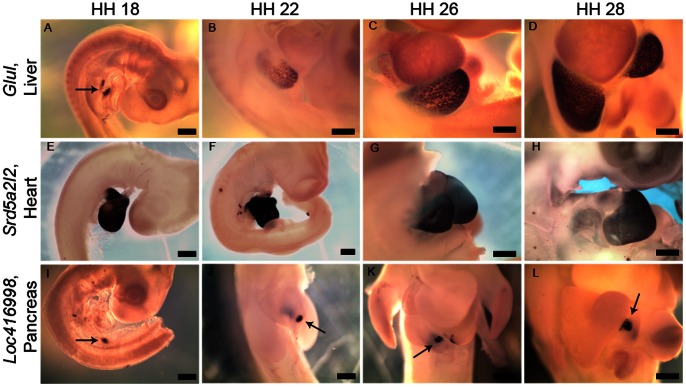
Exclusive expression of metabolism related genes in specific developing organs. (A–L) Whole-mount RNA in situ hybridization showing (A–D) *Glul* is expressed in the developing liver at, (A) HH18 (arrow), (B) HH22, (C) HH26, (D) HH28, (E–F) *Srd5a2l2* is expressed in the developing heart at, (E) HH18, (F) HH22, (G) HH26, (H) HH28, (I–L) *Loc416998* is expressed in the developing pancreas (arrow) at, (I) HH18, (J) HH22, (K) HH26 and (L) HH28. Scale bar 5 mm. Full gene names are presented in Table S6.

Surprisingly, very few MRGs were found to be expressed ubiquitously during the time window of our study. Our data reveals that while many MRGs are expressed in a tissue-restricted manner, most of them are not exclusive to a particular tissue. On the contrary, we found many instances where a particular metabolism-related gene was expressed in diverse tissues which are unrelated to each other. For example lactate dehydrogenase A (*Ldha*) is expressed in 16 different expression domains of 11 different organs or embryonic structures (Fig. 2). Further, 66 genes in our study exhibited expression in 10 or more different patterns within various organs/embryonic structures and 156 genes were expressed in five or more different organs/embryonic structures (Table S3b). On the other hand, the expression of only a few MRGs were found to be restricted to a single organ (Fig. 3).

### Different Embryonic Structures Exhibit Distinct Trends of Acquisition of Metabolic Activity

**Figure 4:**
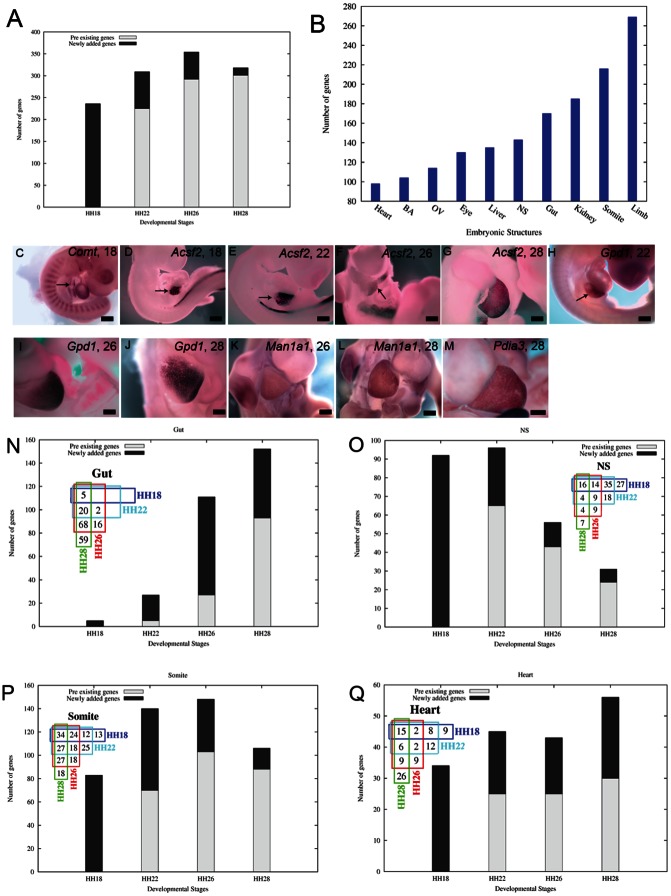
Acquisition of metabolic activity during chicken embryogenesis. (A) Total number of MRGs at different stages of embryonic development. The black portions (Newly added genes) of the bars at HH18, HH22, HH26 and HH28 denote the number of genes whose expression were detectable for the first time at HH18, HH22, HH26 and HH28, respectively. The gray portions (Pre-existing genes) of the bars at HH22, HH26 and HH28 denote the number of genes whose expression was also detectable at HH18, HH22 and HH26, respectively. (B). Total number of unique MRGs expressed in the corresponding embryonic structures. (C–M) Temporal variation in expression of MRGs in the developing liver. (C) *Comt* at HH18, (D–G) *Acsf2* at (D) HH18, (E) HH22, (F) HH26 and (G) HH28, (H–J) *Gpd1* at (H) HH22, (I) HH26 and (J) HH28, (K–L) *Man1a1* at (K) HH26 and (L)HH28 and (M) *Pdia3* at HH28. Arrows point to the developing liver. Scale bar 5 mm. (N–Q) Trends of acquisition of metabolic activity in (N) the gut, (O) the NS, (P) the somites and (Q) the heart. The black portion of the bars at HH18, HH22, HH26 and HH28 denote the number of MRGs whose expression were detectable for the first time at HH18, HH22, HH26 and HH28, respectively (Newly added genes). The gray portions of the bars at HH22, HH26 and HH28 denote the number of MRGs whose expression was also detectable at HH18, HH22 and HH26, respectively (Pre-existing genes). *Inset*, the Venn diagrams show the number of unique genes expressed in unique combinations of stages. (See also Figure S1). Abbreviations: BA – Branchial arches, OV – Otic vesicle, NS – Neural structures. Full gene names are presented in Table S6.

During embryonic development, simpler progenitor structures give rise to a variety of complex organs consisting of many different tissues, each with a distinct metabolic objective. Thus, it would be expected that the total number of MRGs expressed in an embryo will increase with time. Analysis of the total number of MRGs expressed at each of the four developmental stages revealed that the minimum number of genes is expressed at HH18 while the maximum number of genes is expressed at HH26 (Fig. 4A). Most genes that are expressed at a particular stage continue to be expressed in a subsequent stage (compare the gray portion of the bars in Fig. 4A) while progressively less new genes are added to the cohort at each stage (black portions of the bars in Fig. 4A). It should be noted that in this analysis, only the total number of MRGs expressed in the entire embryo at a given stage was considered without taking into account the temporal alterations in the spatial domains of expression. For further analysis we have closely examined the following ten embryonic structures/organs: otic vesicle (OV), eye, neuronal structures (NS), branchial arches (BA), somites, kidney, heart, limbs, liver, and gut. We observed a large variation in the number of MRGs expressed in these embryonic structures (Fig. 4B). For example, heart or BA expressed relatively less MRGs as compared to somites or limbs. When we analyzed the temporal variation in the number of MRGs expressed in any particular structure, we observed distinct trends. In any given tissue, the enzymes that are expressed in it appear at different time points and persist for varying time durations (Fig. 4C–4M). For example, in liver catechol-O-methyltransferase (*Comt*) is detectable at HH18 (Fig. 4C) only while acyl-coA synthetase family member 2 (*Acsf2*) appears at HH18 and persists throughout the time window of the study (Fig. 4D–4G). On the other hand enzymes like glycerol-3-phosphate dehydrogenase 1 (*Gpd1*) (Fig. 4H–4J), mannosidase alpha class 1A, member 1 (*Man1a1*) (Fig. 4K–4L), and protein disulfide isomerase family A, member 3 (*Pdia3*) (Fig. 4M) appear at progressively later stages and persist at least up to stage HH28.

We analyzed the temporal variation in the number of MRGs expressed in nine different embryonic structures (BA, an embryonic structure derived from multiple germ layers, was not included in this analysis) (Fig. 4N–4Q; Fig. S1). Overall, we observed four distinct trends of acquisition of metabolic activity in the nine structures analyzed. In the gut, at HH18, only 5 MRGs are expressed, all of which continue to be expressed till HH28 (Fig. 4N; also see the Venn diagram in the *inset*). Increasing numbers of new MRGs are added to this cohort at progressively later stages (black portions of the bars of Fig. 4N) at the same time most of the genes expressed in a preceding stage continue to be expressed in a subsequent stage (gray portions of the bars of Fig. 4N). Thus, in the gut, the total number of genes expressed at HH28 is very large as compared to the number expressed at HH18. In stark contrast, in the NS, relatively high (92) number of genes are expressed at HH18 and maximum number of MRGs (96) are expressed at HH22, of which only 20 continue to be expressed till HH28 (Fig. 4O; also see the Venn diagram in the *inset*). Decreasing numbers of new MRGs are added to this cohort at progressively later stages. Also there is a steady decline in the number of genes that continue to be expressed from a preceding stage to the next stage (gray portions of the bars of Fig. 4O). Interestingly, in two other ectodermally-derived structures (OV, Eye) also we see the same trend i.e. the number of MRGs expressed at HH18 is relatively high which dramatically decreased with progressive developmental stages (Fig. 4O; Fig. S1B, S1C).

On the other hand, in structures such as somites (Fig. 4P), liver (Fig. S1A), kidney (Fig. S1D) and limb (Fig. S1E) though the number of genes present at HH18 is the lowest amongst all the stages but this number is significantly more compared to that in the gut at HH18 (Fig. 4N). In all these structures, the trend of addition of new genes added to the cohort at HH18 indicates that the peak of tissue-specific MRG expression is around HH26. However, in all these structures other than the limb between HH26 and HH28 there is a decline in both the total number of genes expressed as well as the number of new genes added to the cohort (Fig. 4; Fig. S1). In stark contrast to all other structures in the heart there is a significant increase in the number of new genes added to the existing cohort between stages 26 and 28 (compare the black bars corresponding to stages 26 and 28 in Fig. 4N–4P; Fig. S1A–1E).

### Correlation between Expression of Metabolism Related Genes and Differentiation

**Figure 5:**
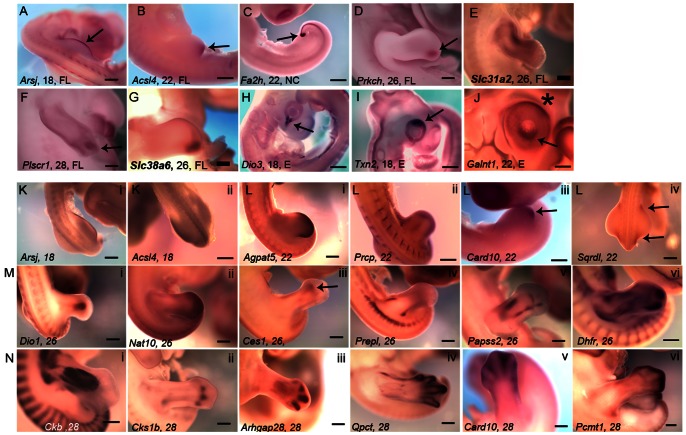
Correlation of expression of metabolism related genes with patterning and differentiation during embryogenesis. (A–C) Whole-mount RNA in situ hybridization showing expression of genes in signaling centers. (A) *Arsj* is expressed in the apical ectodermal ridge (AER) of the fore limb (arrow) at HH18, (B) *Acsl4* is expressed in the zone of polarizing activity of the fore limb (arrow) at HH22, (C) *Fa2h* is expressed in the tail bud (arrow) at HH22. Assymetric expression of MRGs (D–G) in the fore limb, (D) *Prkch* in digit 4 (arrow) at HH26, (E) *Slc31a2* in digit 4 (arrow) at HH26, (F) *Plscr1* in digit 3 (arrow) at HH28, (G) *Slc38a6* in anterior skeletal element (arrow) at HH26 and (H–J) in the eye (H) *Dio3* in ventral eye (arrow) at HH18, (I) *Txn2* in dorsal eye (arrow) at HH18 (J) *Galnt1* in dorsal eye (asterisk) and ventral lens (arrow) at HH22. (K–i to N-vi) Diverse patterns of gene expressions in the developing hind limb, (K-i to K-ii) at HH18, (K-i) *Arsj* in the AER, (K- ii) *Acsl4* in the limb mesenchyme, (L-i to L-iv) four of the eight patterns observed at HH22, (L-i) *Agpat5* in distal edge of limb mesenchyme, (L-ii) *Prcp* center of the limb mesenchyme, (L-iii) *Card10* in the distal posterior mesenchyme (arrow), (L-iv) *Sqrdl* in the base of the limb bud at the anterior and the posterior ends (arrows), (M-i to M-vi) six of the twenty patterns observed at HH26, (M-i) *Dio1* in the developing muscles in the center of the limb bud, (M-ii) *Nat10* in the distal edge of the limb mesenchyme, (M-iii) *Ces1* in an uncharacterized anterior structure (arrow), (M-iv) *Prepl* in uncharacterized structures along the flanks, (M-v) *Papss2* in the developing cartilage, (M-vi) *Dhfr* in uncharacterized pattern including limb muscles, (N-i to N-vi) six of the thirty one patterns at HH28, (N-i) *Ckb* in the limb muscles, (N-ii) *Cks1b* in the tendon, (N-iii) *Arhgap28* in the digits, (N-iv) *Qpct* in the perichondrium, (N-v) *Card10* in the interdigital mesenchyme, (N-vi) *Pcmt1* in the distal margin of the developing limb. Scale bar 5 mm. Full gene names are presented in Table S6.

There are few examples in the literature where MRGs are known to regulate embryonic patterning [2], [7]. We have observed expression of some MRGs in well established signaling centers that play critical roles in early embryonic patterning e.g., apical ectodermal ridge (AER), zone of polarizing activity (ZPA) of the limb and the tail bud (Fig. 5A–5C). In addition, we have observed asymmetric expression of MRGs in several embryonic structures such as the developing digits and the retina (Fig. 5D–5J).

Our study reveals that structures with relatively homogeneous tissue composition such as cranial ganglia (Fig. S2A–S2D) or Liver (Fig. S2E–S2T) have invariant patterns of gene expression as compared to structures such as somites (Fig. S3), kidney (data not shown), and limb (Fig. 5K–i to 5N–vi) which are composed of multiple different tissue or cell types and exhibit a wide variety of expression patterns.

Within the span of our study, many morphogenetic events take place in the limb such as differentiation of cartilage, tendons and muscles [24], apoptosis of interdigital mesenchyme [25] and innervation and vascularization of the limb [26], [27]. One may speculate that as most of the morphogenetic events within the limb, each with its distinct metabolic need, start only after HH18 and progresse throughout the screen period, the number of different domains of expression of MRGs will gradually increase within the time window of the screen, eventually resulting in a large number of expression domains each carrying out its own metabolic objective. In agreement with this, in the developing hind limb at HH18, we observed 2 distinct patterns of MRG expression, which increases to 8 patterns at HH22, to 20 patterns at HH26 and finally to 31 distinct patterns at HH28 (Fig. 5K–i to 5N-vi; also see Fig. 5A; 5B; 5D–G).

### Investigation of Possible Enrichment of Members of Metabolic Pathways in Developing Structures

**Figure 6:**
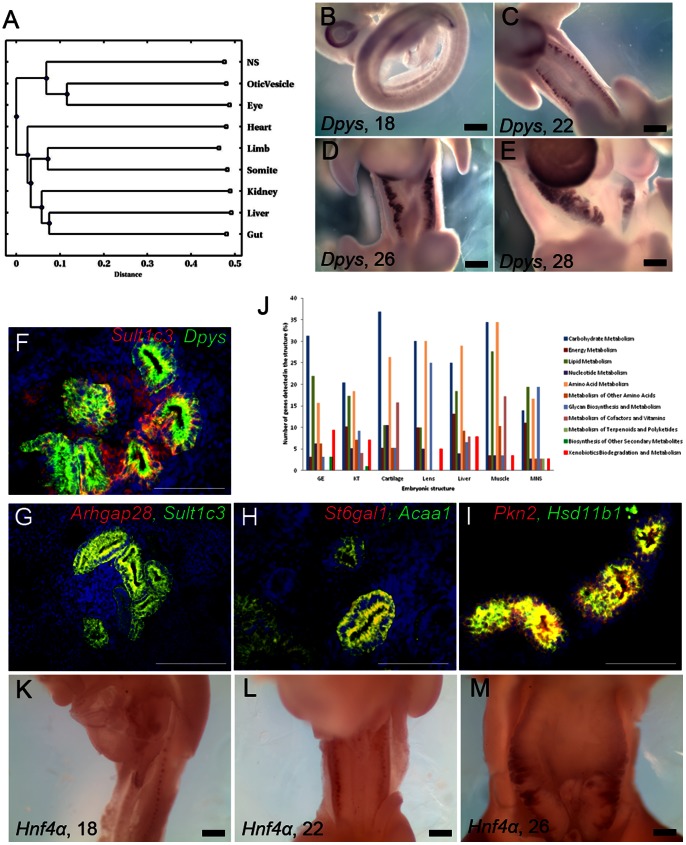
Clustering of metabolism related genes. (A) Tree structure of relative correlation between embryonic structures/organs based on sets of identically co-expressed genes (see Experimental Procedures), (B–E) Expression pattern of kidney tubule (KT) specific gene *Dpys* during development at (B) HH18, (C) HH22, (D) HH26 and (E) HH28. (F–I) Fluorescent double RNA in situ hybridization with transverse sections (16 µm thick) of HH28 chicken embryo showing co-expression of KT specific genes, (F) *Sult1c3* in red and *Dpys* in green, (G) *Arhgap28* in red and *Sult1c3* in green, (H) *St6gal1* in red and *Acaa1* in green, (I) *Pkn2* in red and *Hsd11b1* in green. Scale bar (white) –100 µm. (J) Graph showing distribution of genes belonging to 11 major metabolic pathways within the distinct co-expression groups representing different embryonic structures. (K–M) Expression of *Hnf4α* in the KT at (K) HH18, (L) HH22 and (M) HH26. Scale bar (black) – Panels B to E and K to M, 5 mm.

The analysis thus far suggests that there is a relationship between expressions of MRGs and differentiation. In several developing embryonic structures similar differentiation programs are executed. For example, muscle differentiation takes place in the somites (skeletal muscle), the limbs (skeletal muscle), the gut (smooth muscle) and the heart (cardiac muscle), while cartilage differentiation takes place in the limbs, in the somites, in the sclera of the eyes, in the jaw etc. Thus based on the differentiation programs shared by different embryonic structures there may be similarities in expression signatures of MRGs. To investigate this possibility we compared different embryonic structures for genes that are co-expressed in an identical spatio-temporal pattern. We define a gene as being identically co-expressed in two different structures if the stage of onset of expression for the gene and the duration through which the expression is detectable is identical in both the structures. The sets of identically co-expressed genes were compared between all the nine structures in a pair-wise manner (in this analysis BA, an embryonic structure derived from multiple germ layers, was not included). This analysis resulted in hierarchical clustering (Materials and Methods) of different embryonic structures. We found that the embryonic structures derived primarily from the ectoderm e.g., OV, eye and NS are most closely related to each other. Among these, OV and eye are closer to each other as compared to NS. Similarly, limb and somites clustered together. Also, liver and gut, the two endodermally-derived structures were found to be more closely related to each other than any other structure. In this analysis, heart did not cluster with any of the other structures (Fig. 6A).

Similarity in the MRG expression profiles amongst embryonic structures with similar tissue types, indicated that the cohort of MRGs expressed in a given tissue type is related to the metabolic objective of the tissue which may be manifested as preferential enrichment of members of certain metabolic pathways. Earlier reports suggest that different metabolic pathways are coordinately upregulated in various embryonic structures concomitant with differentiation activities [19], [28].To investigate this possibility it is imperative to analyze a set of genes that are expressed within the same cell. For this purpose, we chose several well-defined expression patterns within embryonic structures where many MRGs are likely to be expressed together. The following seven tissue/embryonic structures were selected for this analysis: 1) kidney tubules (KT), 2) lens, 3) inner lining of the gut (GE), 4) muscle, 5) cartilage, 6) multiple neuronal structures (MNS) and 7) liver. We observed a well-defined expression pattern in KT (Fig. 6B–6E), where 127 genes are expressed. To investigate the possibility that most of these genes are expressed in the same cell we carried out pair-wise analysis of randomly selected seven such genes using fluorescent double mRNA *in situ* hybridization (Fig. 6F–6I). Our data demonstrates that all the selected pairs of genes are co-expressed in the KT (Fig. 6F–6I). The other two embryonic structures where many genes are likely to be co-expressed are the lens (45 genes) (Fig. S4), and the inner lining of the gut (GE, 42 genes) (Fig. S5). There are certain tissues such as muscle, cartilage and neurons which occur in several embryonic structures. We observed that most of the genes that are expressed in any one of these structures are also present in all other embryonic structures comprising of the same tissue. We have assigned 57 genes to the muscle cluster many of which are expressed in a muscle-like pattern in the limbs (Fig. S6A; S6D; S6G; S6J), myotome-like pattern in the somites (Fig. S6B; S6H; S6K) and in the heart (Fig. S6C; S6F; S6I; S6L). Similarly we defined a set of 30 genes as a signature for the developing cartilage which were found to be expressed in the cartilage domain of developing limb. Similarly, we have designated a set of 81 genes as neuron-specific genes which were expressed in multiple neuronal structures (MNS). As 78% of liver cells are hepatocytes [29], [30] and the patterns of expression observed in the liver are invariant (Fig. S2) it may be assumed that most of the 135 liver-specific genes are expressed in the same cell.

We used KEGG [31], [32] annotations to identify the metabolic pathways to which the co-expressed genes in each of the seven well-defined tissues/structures belong. The number of genes within each embryonic structure with KEGG metabolic pathway annotations are as follows: 1) KT: 69 out of 127, 2) lens: 20 out of 45, 3) GE: 24 out of 42, 4) muscle: 29 out of 57, 5) cartilage: 19 out of 30, 6) MNS: 36 out of 81 and 7) liver: 76 out of 135. We examined for possible enrichment of members of 11 major metabolic pathways amongst these embryonic structures (Fig. 6J). Our analysis revealed that carbohydrate metabolism and amino acid metabolism are the two major metabolic pathways in all of these embryonic structures other than the MNS, where lipid metabolism and glycan metabolism are the two major pathways. Lipid metabolism is very prominent in the GE as well, while glycan metabolism is a major pathway in the lens. It may be noted that members of the lipid metabolism pathway are least represented in the cartilage and the lens. Carbohydrate metabolism is the major metabolic pathway in cartilage and GE while amino acid metabolism is the major metabolic pathway in the liver. Xenobiotic degradation pathway members are represented primarily in the liver, KT and GE (Fig. 6J). We further investigated possible enrichment of specific member pathways of each of the previously mentioned 11 major metabolic pathways in these embryonic structures. There are 143 specific pathways described in the KEGG database of which we found tissue-restricted expression of genes belonging to 62 specific pathways. We did not find enrichment of any of these specific pathways in the seven embryonic structures we analyzed. Despite conducting a comprehensive expression screen of MEs we could not detect differential enrichment of metabolic pathways within distinct structures. Thus we hypothesize that unique combinations of MRGs, and not specific metabolic pathways, are upregulated in spatio-temporally restricted manner during development.

**Figure 7:**
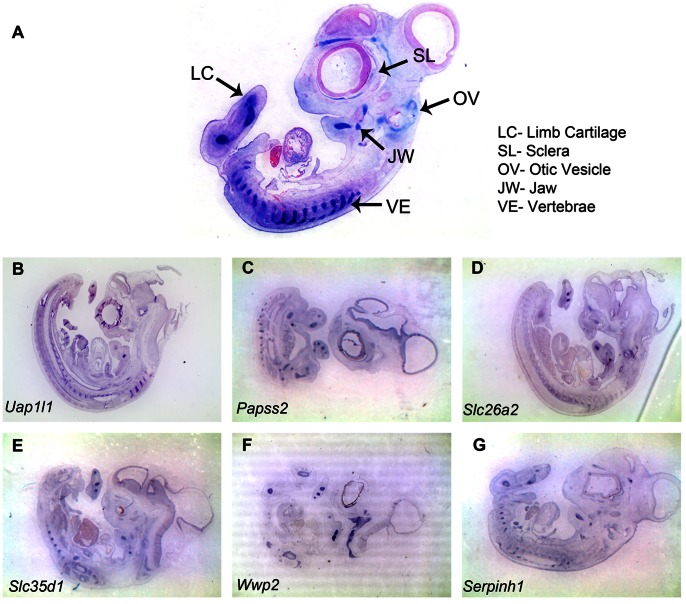
Expression patterns of cartilage specific metabolism related genes. (A) Alcian blue staining of 8 µm thick sagittal section of HH28 chicken embryo highlights various cartilaginous structures in developing embryos such as limb cartilage (LC), vertebrae (VE), jaw (JW), otic vesicle (OV), sclera (SL). Scale bar 5 mm**.** (B–G) RNA in situ hybridization of 8 µm thick Sagittal sections of HH28 chicken embryo showing expression of (B) *Uap1l1*, (C) *Papss2*, (D) *Slc26a2*, (E) *Slc35d1*, (F) *Wwp2,*(G) *Serpinh1*, genes in various cartilaginous structures. Full gene names are presented in Table S6.

All cartilaginous structures display alcian blue staining [33]. To further validate the cartilage specific MRG signature we compared longitudinal sections of HH28 chick embryo stained with alcian blue to S-ISH pattern for members of cartilage ME signature. As expected these MEs are expressed in different cartilaginous structures found within the developing embryo (Fig. 7 A–G). Of the validated members of the cartilage ME signature, UAP1L1 participates in the formation of the activated sugar donor while *Papss2* has been speculated to be involved in the formation of the activated sulphate donor [34]. SLC26A2 is likely to be involved in sulfate transport [35] while SLC35D1 transports the activated sugar precursors to the lumen of the endoplasmic reticulum [36] where glycosylation takes place. Therefore, it is possible that UAP1L1, PAPSS2, SLC26A2 and SLC35D1 collaborate for the biosynthesis of chondroitin sulfate, a major component of cartilage.

### Microarray Data-mining of KT Genes Leads to Identification of HNF4α as a Likely Regulator of Kidney Differentiation

Based on our data, we speculate that MRGs co-expressed in a given cell type are downstream effectors in a differentiation cascade. Co-expressed genes are likely to share gene regulatory mechanisms [37]. Large cohorts of such co-expressed genes, uncovered in our screen, may be used to discover novel upstream regulators of such cohorts and in turn regulators of differentiation of that tissue. One of the largest sets of co-expressed genes identified in our study is the one expressed in KT. We queried “GEO Profiles (http://www.ncbi.nlm.nih.gov/geoprofiles/)” using individual genes belonging to the kidney tubule co-expression cluster of MRGs. From the results we noted down the experiments in which the expression of a given gene was down-regulated in a loss-of-function mutation of a putative regulatory gene or the expression of a given genes was up-regulated in a gain-of-function mutation of a putative regulatory gene. Any gene that appeared in many such results was a putative candidate gene regulating the expression of genes belonging to the kidney tubule co-expression cluster of MRGs. We found HNF4α to be one such candidate gene.

Though HNF4α is known to be important for liver morphogenesis [38], so far no report of it regulating any aspect of kidney tubule development has been made. HNF4α is a transcription factor which has been earlier implicated in regulation of *Dio1* expression [39], one of the members of KT co-expression group. However, bioinformatics analysis done in a study exploring genes showing compartment specific expression in developing kidney, revealed that 22 out of 25 early proximal kidney tubule-specific genes in mouse have HNF4α binding site in their promoters [40]. We therefore investigated the expression pattern of *Hnf4α* in the chick embryonic kidney tubules between HH18-HH28 (Fig. 6K–6M). We observed that *Hnf-4 alpha* is expressed in the KT at HH18 (Fig. 6K) which precedes the expression of most of the KT co-expression group genes and the expression continues up to HH26 (Fig. 6L–6M).

## Discussion

We have carried-out a genome-wide whole-mount RNA *in situ* hybridization-based expression screen for MRGs to establish correlation between morphogenesis and concomitant acquisition of metabolic activity, as judged by expression of MRGs. To our surprise, we observed that ∼25% of the genes screened exhibited tissue restricted expression while ∼2% exhibited ubiquitous expression, suggesting that MRGs play tissue specific roles.

When we analyzed the temporal trend of acquisition of MRG expression we observed distinctive trends in the embryonic structures that we have analyzed. The well-defined trend of acquisition of MRG expression in every structure indicates that there is a surge of metabolic activity coinciding with the initiation of a cellular process such as specification, differentiation, patterning, or a combination of these, which is about to take place in these tissues. In this context, we would like to draw a parallel to the differentiation of dividing progenitor cells to non-dividing differentiated cells during embryonic development of Xenopus retina wherein there is a sudden change in metabolic profile from glycolysis to oxidative phosphorylation [41]. Also, Ozbudak et. al., reported a sharp change in expression of many genes, including members of certain metabolic pathways, between the cells of the posterior presomitic mesoderm and the anterior presomitic mesoderm on either side of the presumptive somitic determination front [28]. In absence of detailed correlative molecular studies, it is difficult to accurately predict the relationship between the surge of metabolic activity in a given structure and the cellular processes that follow. However, from existing literature**,** certain tentative correlations may be made. For example, in the forming neuronal structures, the peak of MRG expression was observed at HH22 (Fig. 4O). In chick, onset of neurogenesis in most embryonic structures takes place around this time and continues till HH27 [42]–[44]. While induction and early patterning of the gut tube in chick is completed before HH18 differentiation begins around HH30 [45]. We have observed the peak of MRG expression in the gut at HH28 (Fig. 4N), with a sharp increase in acquisition of MRG expression in this tissue taking place around HH26. The close association of the peak of MRG expression observed in the gut tube in our study (HH28) with the onset of differentiation of the gut tube suggests that the surge in metabolic activity precedes or coincides with the beginning of gut differentiation. Heart tube morphogenesis is largely accomplished by the first time point of our study i.e., HH18 [46]. In the subsequent phase of cardiac morphogenesis, several differentiated cell types are formed. We see a relative dip in MRG expression in the heart between HH18 and HH26, followed by a sharp increase at HH28. This increase in metabolic activity is likely to facilitate differentiation of multiple different cardiac structures such as the heart valves, which take place around this stage. In this context it is interesting to note that glutaminyl-peptide cyclotransferase (*Qpct*) is very specifically expressed in the forming heart valves at HH28 (Fig. 1P). In the limb, soon after HH26, cartilage, muscle, and tendon differentiation begins [24]. Commensurate with this finding, we see a sharp rise in MRG expression in the limb from HH22 to HH26 which does not decline at HH28 (Fig. S1E).

The following two lines of evidences generated from our study indicates that expression of MRGs is correlated with differentiation. First, we observed a surge in acquisition of MRG expression coincident with onset of differentiation. Second, embryonic structures with similar constituent tissue types express similar sets of MRGs. For example, NS, OV and eye contain neurons and were found to be most similar to each other in terms of the cohort of MRG expression. Similarly, limb and somites share most of the differentiating tissue types and were found to be most closely related to each other (Fig. 6A). Shared cohorts of MRGs expressed in embryonic structures sharing tissue types indicates that these cohorts of MRGs might be responsible for accomplishment of the metabolic objective necessary for differentiation of the constituent tissues of these embryonic structures. This prompted us to investigate the possible enrichment of members of individual metabolic pathways in differentiating structures. For this purpose, we used collections of MRGs that are expressed within well-defined structures e.g. KT, lens, GE etc. In absence of comprehensive information regarding pathway affiliation for many of the MRGs as well as association of the same MRG with multiple pathways, it is not possible to determine statistically significant enrichment of specific pathways in various embryonic structures. Nevertheless, our data revealed hints at possible enrichment of certain major metabolic pathways within some of the embryonic structures analyzed (Fig. 6J). For example, it appears that amino acid metabolism is the major metabolic pathway in the liver. This finding is in agreement with an earlier observation [19]. We have also observed that, as opposed to any other embryonic structure analyzed by us, in MNS lipid metabolism and glycan biosynthesis are the major metabolic pathways. This may be expected since neurons have extensive processes which require comparatively high levels of membrane lipid biosynthesis. Also, glycoproteins play major roles in axon guidance and synaptogenesis [47]. The other significant observation made in this context was the enrichment of members of the xenobiotic degradation pathways specifically in the liver, KT and GE which are the major sites of elimination of toxic substances in the body [48].

Our data strongly suggests that there is a correlation between differentiation and acquisition of metabolic activity, prompting us to investigate the enrichment of members of specific metabolic pathways within the embryonic structures mentioned above. We could not find enrichment of any specific metabolic pathway in any of the embryonic structures analyzed. There may be several technical reasons for this apparent failure to discover emergence of tissue specific metabolic pathways during differentiation such as non-saturation of the screen, use of a relatively less sensitive detection method and the absence of metabolic pathway annotation for many genes. In addition, there may be physiological reasons as to why we did not find any tissue specific metabolic pathway enrichment. However, a thorough review of literature on the roles of MRGs expressed in cartilage domain indeed showed that some of the MRGs expressed in this domain might be complementing each other towards chondroitin sulphate metabolism. Thus mapping the members onto the KEGG pathways may not necessarily provide insight regarding a group of MRGs collaborating towards the metabolic objective of a differentiating morphological structure. Detailed analysis based on known metabolic reactions that these genes participate in may be necessary for this. Our study provides the impetus to identify other such groups of collaborating enzymes within co-expression clusters.

We have observed that the same gene is expressed in multiple structures at different time points (Fig. 2; Table S3a) and the expression of different genes become apparent at different time points in a given structure (Fig. 4C). Taken together, we speculate that it is not upregualtion of one or few specific pathways, rather hierarchical action of unique combinations of MRGs which bring about the phenotypic changes associated with differentiation. Further, the developing embryo has access to a variety of metabolites deposited in the egg, which may be used as substrates by the MRGs expressed during embryogenesis. Thus in our opinion, it is not necessary that all members of a particular metabolic pathway have to be expressed in a given structure in order to achieve its metabolic objective, rather an eclectic collection of individual MRGs may be sufficient. Further, activities for most of the MRGs were discovered and characterized using directed assays under *in vitro* condition. Thus, there remains a possibility that some of these MRGs possess functions in addition to the canonical ones and in absence of the knowledge of entire range of functions for these genes it may not be possible to adequately understand the metabolic objective a cohort of MRGs represent.

Our study provides many lines of evidence suggesting that some of the genes exhibiting tissue-restricted expression may be playing roles in embryonic development using non-conventional or even non-catalytic activities. Many members of housekeeping metabolic pathways such as glycolysis, gluconeogenesis, TCA cycle, fatty acid metabolism etc., were included in our screen of which the expression for majority could not be detected while a few exhibited tissue specific expression (Table S4). There have been previous reports where members of glycolysis pathway have been reported to be differentially expressed in tissues within developing embryos [19], [49]. A significant majority of genes belonging to these housekeeping metabolic pathways, for which we could detect expression in our screen, have been previously reported to either possess non-conventional roles or be associated with genetic loci for various human diseases (Table S4). In this context it is worth mentioning that of the 410 MRGs for which we could detect expression in our screen 82 genes were found to be associated with human diseases of which the expression of 52 genes were found to be in the same tissue that is affected in the associated disease condition (Table S5). Furthermore, we found that for a significant majority of these 82 genes the tissue-restricted expression patterns observed in the chick embryos are conserved in mice and Zebrafish (Table S5). Also, many of the metabolic enzymes demonstrated to possess tissue specific roles are expressed in a tissue-restricted manner in our screen (Table S1). Thus, the database of tissue-restricted expression of MRGs created through this work may indeed serve as a filter to systematically select the ones with potential tissue-specific roles.

The unprecedented scale of this study investigating spatio-temporally dynamic expression patterns of MRGs during development allowed us to draw a correlation between differentiation and expression of MRGs. Subsequent molecular genetic analysis of MRGs, in conjunction with metabolite profiling (metabolomics) of particular embryonic structures, should elucidate the specific role(s) of MRGs during differentiation.

## Supporting Information

Figure S1 **Trends of acquisition of metabolic activity in five embryonic structures.** (A–E) Trends of acquisition of metabolic activity in (A) the liver, (B) the OV, (C) the eye, (D) the kidney and (E) the limb. The black portion of the bars at HH18, HH22, HH26 and HH28 denote the number of MRGs whose expression were detectable for the first time at HH18, HH22, HH26 and HH28, respectively (Newly added genes). The gray portions of the bars at HH22, HH26 and HH28 denote the number of MRGs whose expression was also detectable at HH18, HH22 and HH26, respectively (Pre-existing genes). *Inset*, the Venn diagrams show the number of unique genes expressed in unique combinations of stages. OV – Otic vesicle.
(TIF)

Figure S2 **Invariant pattern of expression of metabolism related genes in developing cranial ganglia and liver.** (A–D) Whole-mount RNA in situ hybridization showing expression of genes in developing cranial ganglia (asterisk) at HH18, (A) *Gmppb*, (B) *Coq2*, (C) *Ndst2* and (D) *Smpd3*. (E–T) Whole-mount RNA in situ hybridization showing expression of genes in developing liver, (E–H) at HH18, (E) *Lcat* (arrow), (F) *Uqcc* (arrow), (G) *Noxa1* (arrow), (H) *Acsf2* (arrow), (I–L) at HH22, (I) *Glul*, (J) *Gpd1*, (K) *Gmps*, (L) *Acsf2* (arrow), (M–P) at HH26, (M) *Got1*, (N) *Fh*, (O) *Gart*, (P) *B3gat2*, (Q–T) at HH28, (Q) *Dpys*, (R) *Glul*, (S) *Gmps*, (T) *Hadha.* Scale bar 5 mm
(TIF)

Figure S3 **Diverse patterns of expression of metabolism related genes in the developing somites.** (A–P), Whole-mount RNA in situ hybridization showing expression of genes in the developing somites, (A–D) at HH18, (A) *Loc425735*, (B) *Plod1*, (C) *Chst15*, (D) *Wnk2*, (E–H) at HH22, (E) *Dhfr*, (F) *Dio3*, (G) *Arsb*, (H) *Pcmt1*, (I–L) at HH26, (I) *Pdia4*, (J) *Serpinh1*, (K) *Qpct*, (L) *Ckb*, (M–P) at HH28, (M) *Tgm2*, (N) *Plb1*, (O) *Ckb*, (P) *Psma2.* Scale bar 5 mm
(TIF)

Figure S4 **Co-expression group of genes expressed in the eye lens.** (A–L), Whole-mount RNA in situ hybridization showing expression of MRGs in the eye lens at HH18. (A) *Ddah1*, (B) *Ppp2r4*, (C) *B3galt2*, (D) *Wnk2*, (E) *Nat1*, (F) *Asns*, (G) *Loc768721*, (H) *Adamts7*, (I) *Slc16a6*, (J) *Snf1lk*, (K) *Syk*, (L) *C1galt1.* Scale bar 5 mm
(TIF)

Figure S5 **Co-expression group of genes expressed in the gut tube epithelium.** (A–J), Whole-mount RNA in situ hybridization showing expression of MRGs in the gut tube epithelium (arrow) at HH28. (A) *Noxa1*, (B) *Pmm2*, (C) *Plscr1*, (D) *Xdh*, (E) *Slc40a1*, (F) *Asah1*, (G) *Entpd6,* (H) *Fh*, (I) *Plb1*, (J) *Slc27a6.* Scale bar 5 mm
(TIF)

Figure S6 **Expression patterns of muscle specific metabolism related genes.** Whole-mount RNA in situ hybridization showing expression of (A–C) *Ckb* at HH28, (A) fore limb, (B) somites, (C) heart, (D–F) *Dyrk3* at HH28, (D) fore limb, (E) somites, (F) heart, (G–I) *Adprhl1* at HH28, (G) fore limb, (H) somites, (I) heart, (J–L) *Usp13* at HH28, (J) fore limb, (K) somites, (L) heart,. Scale bar 5 mm
(TIF)

Table S1 **Previously reported tissue specific roles of metabolic enzymes – The genes shaded in green are the ones whose expression was investigated in this screen.** ¥ - There is no report of in vivo investigation of the role of vertebrate *Acsl4*. However, the Drosophila homolog of the gene, *Acsl*, has been studied in vivo.
(PDF)

Table S2 **Generation of target gene list for the expression screen of metabolism related genes.**
(PDF)

Table S3 **Many metabolism related genes are expressed in a tissue-restricted manner but not in a tissue-exclusive manner.** The number of different embryonic structures/organs a gene is expressed in (Table S3a). The total number of different patterns in which a gene is expressed (Table S3b, all different embryonic structures/organs combined).
(PDF)

Table S4 **The house-keeping genes that exhibit tissue-specific expression are likely to have specialized functions.** List of genes belonging to glycolysis/gluconeogenesis (Table S4a), TCA cycle (Table S4b) and fatty acid metabolism (Table S4c) pathways**.** The tissue in which these genes are expressed as well as the associated disease names are provided in adjacent columns.
(PDF)

Table S5 **Metabolism related genes with conserved expression domains across vertebrate species are associated with diseases wherein the affected tissue is the one expressing the gene.** Column “G” lists the embryonic structures in which expression of a gene was detected. Column “H” has the image file ID for the gene’s expression pattern in Mouse or Zebrafish. Column “H” has an entry if the expression of the gene is conserved in Zebrafish or mouse and in one of the embryonic structures whose adult counterpart is affected in a disease. Column “I” has the image file ID for the gene’s expression pattern in Mouse or Zebrafish. Column “I” has an entry if the conserved structure in which expression is observed in chick/mouse/fish is not the one associated with a disease listed in OMIM database.
(PDF)

Table S6 **List of all the Chicken EST clones and full names of the genes used in this study.**
(PDF)
