# Supplementary figures and images for: A Genome-Wide Screen Indicates Correlation between Differentiation and Expression of Metabolism Related Genes
Source: PLoS One. 2013 May 22;8(5):e63670. doi: 10.1371/journal.pone.0063670 (PMC3661535; doi:10.1371/journal.pone.0063670)

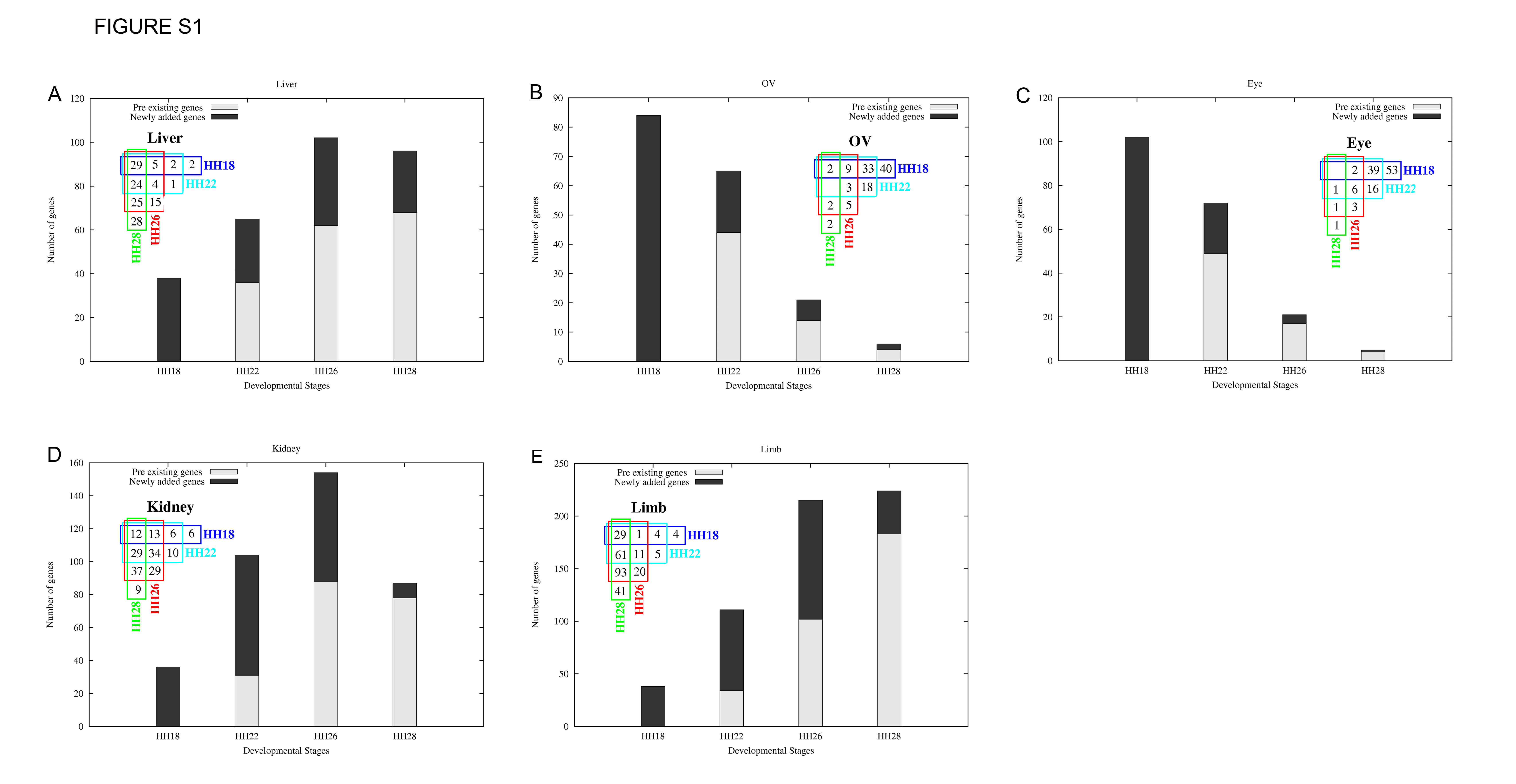

Supplement: Figure S1 — Trends of acquisition of metabolic activity in five embryonic structures. (A–E) Trends of acquisition of metabolic activity in (A) the liver, (B) the OV, (C) the eye, (D) the kidney and (E) the limb. The black portion of the bars at HH18, HH22, HH26 and HH28 denote the number of MRGs whose expression were detectable for the first time at HH18, HH22, HH26 and HH28, respectively (Newly added genes). The gray portions of the bars at HH22, HH26 and HH28 denote the number of MRGs whose expression was also detectable at HH18, HH22 and HH26, respectively (Pre-existing genes). Inset, the Venn diagrams show the number of unique genes expressed in unique combinations of stages. OV – Otic vesicle. (TIF) [file pone.0063670.s001.tif]

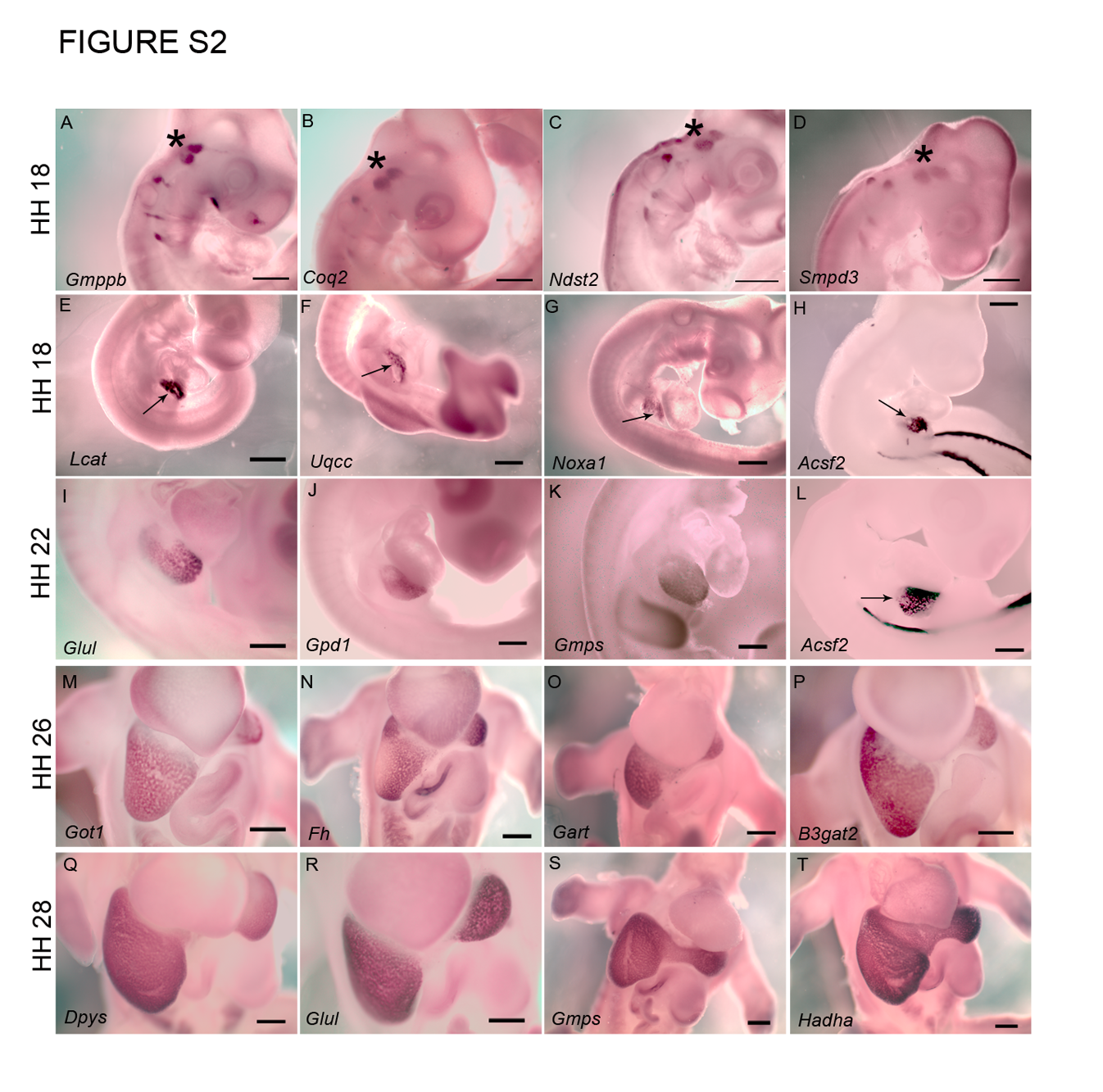

Supplement: Figure S2 — Invariant pattern of expression of metabolism related genes in developing cranial ganglia and liver. (A–D) Whole-mount RNA in situ hybridization showing expression of genes in developing cranial ganglia (asterisk) at HH18, (A) Gmppb, (B) Coq2, (C) Ndst2 and (D) Smpd3. (E–T) Whole-mount RNA in situ hybridization showing expression of genes in developing liver, (E–H) at HH18, (E) Lcat (arrow), (F) Uqcc (arrow), (G) Noxa1 (arrow), (H) Acsf2 (arrow), (I–L) at HH22, (I) Glul, (J) Gpd1, (K) Gmps, (L) Acsf2 (arrow), (M–P) at HH26, (M) Got1, (N) Fh, (O) Gart, (P) B3gat2, (Q–T) at HH28, (Q) Dpys, (R) Glul, (S) Gmps, (T) Hadha. Scale bar 5 mm (TIF) [file pone.0063670.s002.tif]

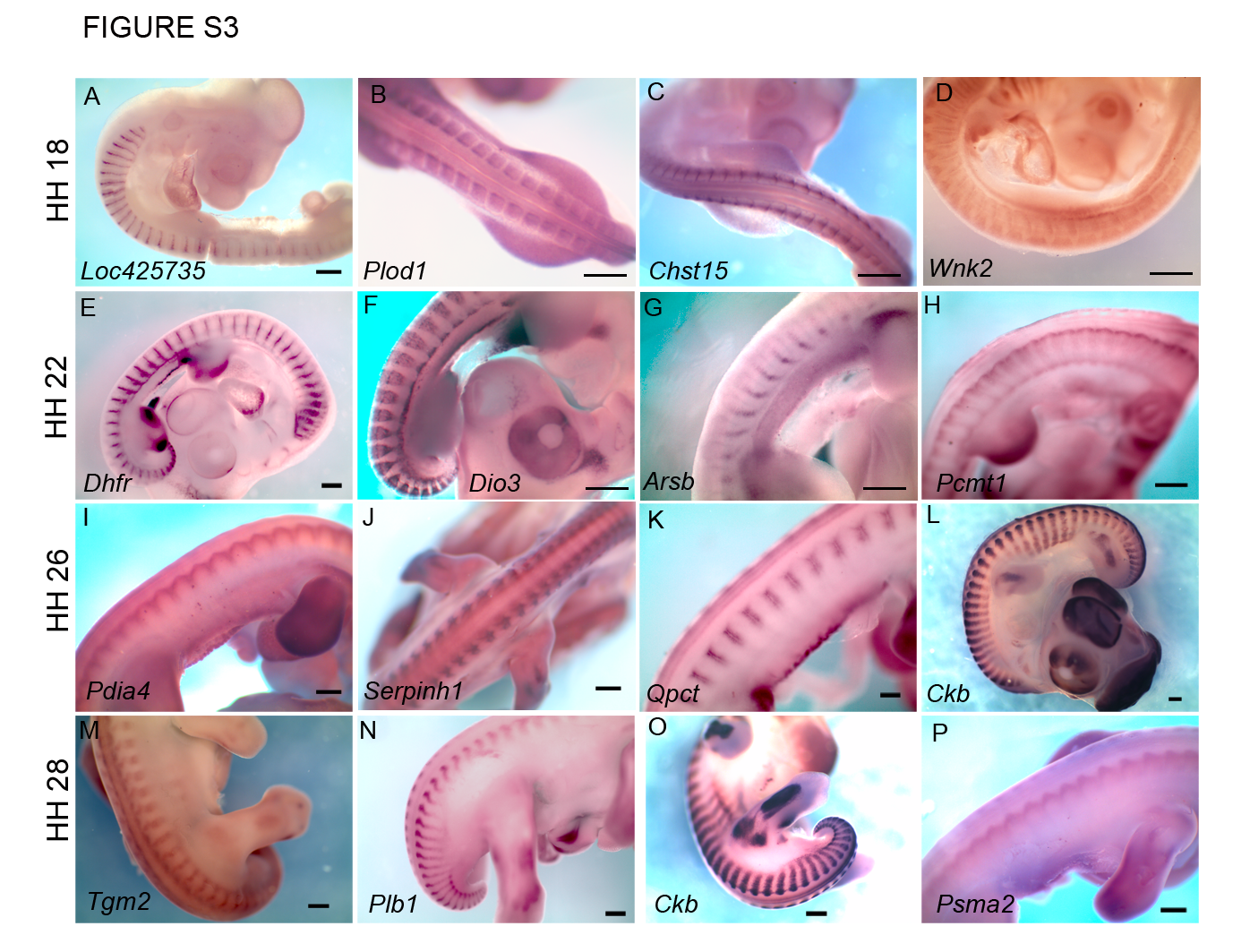

Supplement: Figure S3 — Diverse patterns of expression of metabolism related genes in the developing somites. (A–P), Whole-mount RNA in situ hybridization showing expression of genes in the developing somites, (A–D) at HH18, (A) Loc425735, (B) Plod1, (C) Chst15, (D) Wnk2, (E–H) at HH22, (E) Dhfr, (F) Dio3, (G) Arsb, (H) Pcmt1, (I–L) at HH26, (I) Pdia4, (J) Serpinh1, (K) Qpct, (L) Ckb, (M–P) at HH28, (M) Tgm2, (N) Plb1, (O) Ckb, (P) Psma2. Scale bar 5 mm (TIF) [file pone.0063670.s003.tif]

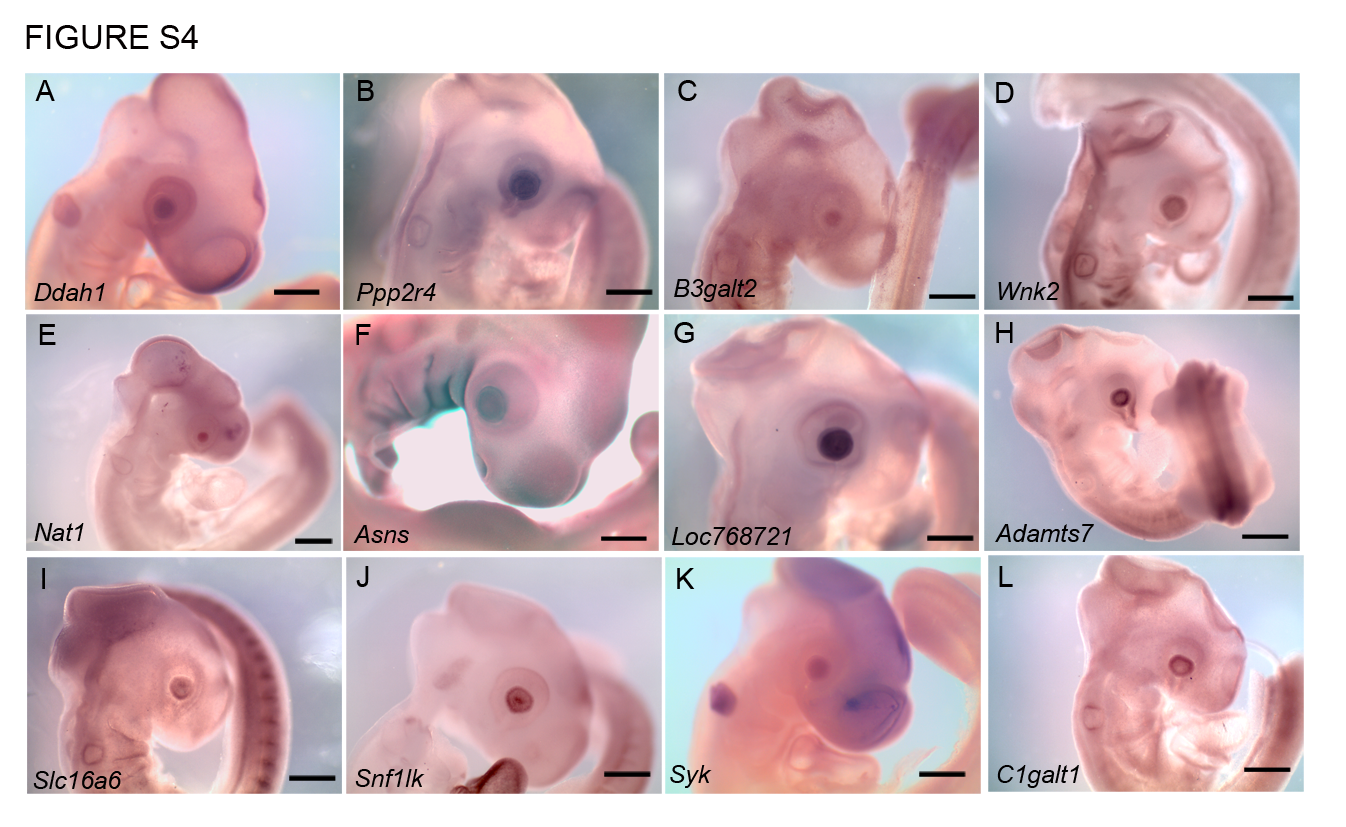

Supplement: Figure S4 — Co-expression group of genes expressed in the eye lens. (A–L), Whole-mount RNA in situ hybridization showing expression of MRGs in the eye lens at HH18. (A) Ddah1, (B) Ppp2r4, (C) B3galt2, (D) Wnk2, (E) Nat1, (F) Asns, (G) Loc768721, (H) Adamts7, (I) Slc16a6, (J) Snf1lk, (K) Syk, (L) C1galt1. Scale bar 5 mm (TIF) [file pone.0063670.s004.tif]

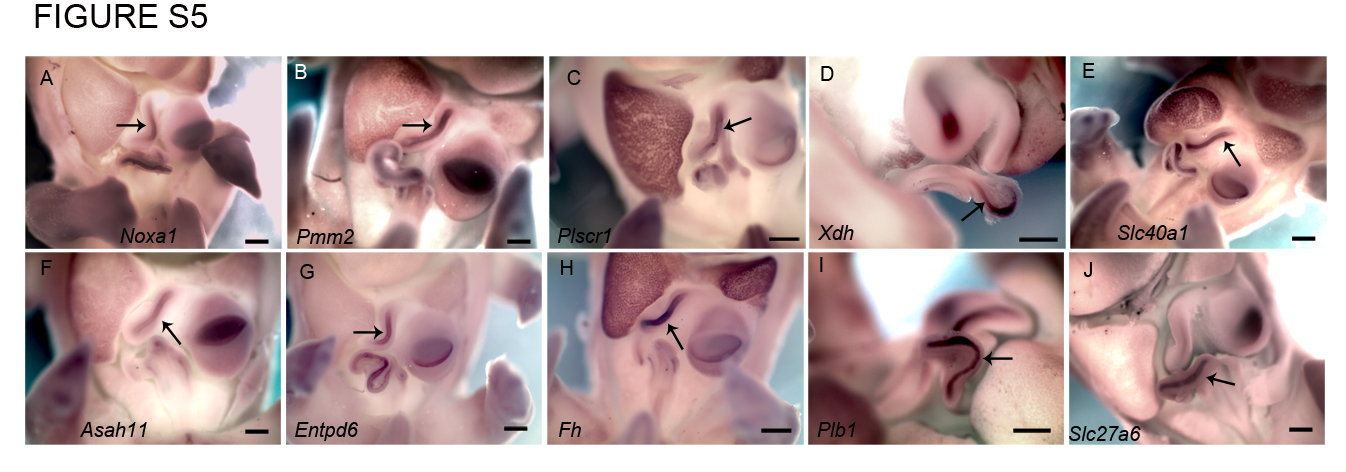

Supplement: Figure S5 — Co-expression group of genes expressed in the gut tube epithelium. (A–J), Whole-mount RNA in situ hybridization showing expression of MRGs in the gut tube epithelium (arrow) at HH28. (A) Noxa1, (B) Pmm2, (C) Plscr1, (D) Xdh, (E) Slc40a1, (F) Asah1, (G) Entpd6, (H) Fh, (I) Plb1, (J) Slc27a6. Scale bar 5 mm (TIF) [file pone.0063670.s005.tif]

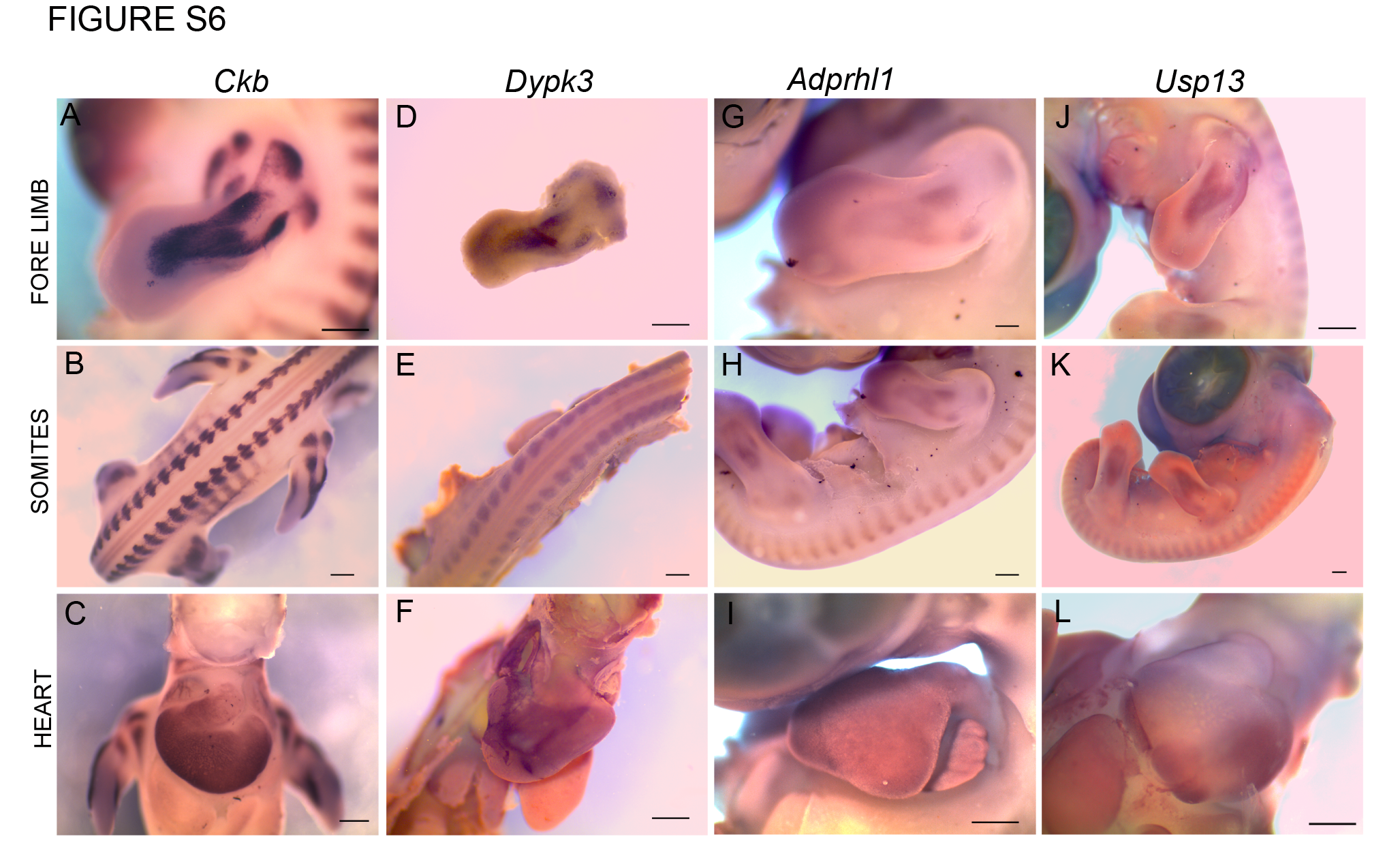

Supplement: Figure S6 — Expression patterns of muscle specific metabolism related genes. Whole-mount RNA in situ hybridization showing expression of (A–C) Ckb at HH28, (A) fore limb, (B) somites, (C) heart, (D–F) Dyrk3 at HH28, (D) fore limb, (E) somites, (F) heart, (G–I) Adprhl1 at HH28, (G) fore limb, (H) somites, (I) heart, (J–L) Usp13 at HH28, (J) fore limb, (K) somites, (L) heart,. Scale bar 5 mm (TIF) [file pone.0063670.s006.tif]
