# Supplementary material for: A Genome-Wide Screen Indicates Correlation between Differentiation and Expression of Metabolism Related Genes
Source: PLoS One. 2013 May 22;8(5):e63670. doi: 10.1371/journal.pone.0063670 (PMC3661535; doi:10.1371/journal.pone.0063670)
Supplement: Table S1 — Previously reported tissue specific roles of metabolic enzymes – The genes shaded in green are the ones whose expression was investigated in this screen. ¥ - There is no report of in vivo investigation of the role of vertebrate Acsl4. However, the Drosophila homolog of the gene, Acsl, has been studied in vivo. (PDF) [file pone.0063670.s007.pdf]

		TABLE S1-Diversity of expression patterns of metabolism related genes	
**S. No.**	**Gene name**	**Title of the paper**	**Reference**
		**DEVELOPMENT RELATED**	
1	TH	Tyrosine hydroxylase is expressed during early heart development and is required for cardiac chamber formation	Cardiovascular Research (2010) 88, 111–120
2	HSD17B 12	Hydroxysteroid (17 b) Dehydrogenase 12 Is Essential for Mouse Organogenesis and Embryonic Survival	Endocrinology, April 2010, 151(4):1893–1901
3	FUCM	Male-like sexual behavior of female mouse lacking fucose mutarotase	BMC Genetics 2010, 11:62
4	TGM2	Transglutaminase 2 regulates early chondrogenesis and glycosaminoglycan synthesis	Mechanisms of Development 1 2 8 (2 0 1 1) 2 3 4 –2 4 5
5	WWP2	The E3 ubiquitin ligase Wwp2 regulates craniofacial development through mono-ubiquitylation of Goosecoid	Nat Cell Biol. 2011 January ; 13(1): 59–65.
6	ACSL ¥	Drosophila long-chain acyl-CoA synthetase acts like a gap gene in embryonic segmentation	Dev Biol. 2011 May 15;353(2):259-65
7	CHSY1	Chondroitin sulfate synthase 1 (Chsy1) is required for bone development and digit patterning	Developmental Biology 363 (2012) 413–425
		**NOVEL CELLULAR ROLE**	
8	GAPDH	S phase activation of the histone H2B promoter by OCA-S, a coactivator complex that contains GAPDH as a key component.	Cell. 2003 Jul 25;114(2):255-66
9	GAPDH	Glyceraldehyde-3-phosphate dehydrogenase interacts with Rab2 and plays an essential role in endoplasmic reticulum to Golgi transport exclusive of its glycolytic activity.	J Biol Chem. 2004 Dec 24;279(52):54046-52
11	Arg5,6	Regulation of gene expression by a metabolic enzyme	Science. 2004 Oct 15;306(5695):482-4.
10	GAPDH	The glycolytic enzymes, glyceraldehyde-3-phosphate dehydrogenase, triose-phosphate isomerase, and pyruvate kinase are components of the K(ATP) channel macromolecular complex and regulate its function	J Biol Chem. 2005 Nov 18;280(46):38464-70
12	PKM2	Nuclear PKM2 regulates β-catenin transactivation upon EGFR activation	Nature. 2011 Dec 1;480(7375):118-22
13	IMPD	Metabolic Enzyme IMPDH Is Also a Transcription Factor Regulated by Cellular State	Mol Cell. 2012 May 31. [Epub ahead of print]
		**DISEASE RELATED**	
13	IMPDH1	Mutations in the inosine monophosphate dehydrogenase 1 gene (IMPDH1) cause the RP10 form of autosomal dominant retinitis pigmentosa	Hum Mol Genet. 2002 March 1; 11(5): 559–568
15	SMPD3	A deletion in the gene encoding sphingomyelin phosphodiesterase 3 (Smpd3) results in osteogenesis and dentinogenesis imperfecta in the mouse	Nature Genetics 37, 803 - 805 (2005)
14	SMPD3	Neutral Sphingomyelinase (SMPD3) Deficiency Causes a Novel Form of Chondrodysplasia and Dwarfism That Is Rescued by Col2A1-Driven smpd3 Transgene Expression	Am J Pathol. 2007 July; 171(1): 153–161
16	CHSY1	Loss of CHSY1, a secreted FRINGE enzyme, causes syndromic brachydactyly in humans via increased NOTCH signaling	Am J Hum Genet. 2010 Dec 10;87(6):768-78.

Note:

(green) These genes were included in the screen described in this manuscript. Expression could be detected for all such genes whose specialized roles have been described in the literature and were included in the screen.

¥ The vertebrate homolog of ACSL, ACSL4, was included in our screen. ACSL4 is expressed in the ZPA of HH22 chick limb bud.
