# Supplementary material for: A Genome-Wide Screen Indicates Correlation between Differentiation and Expression of Metabolism Related Genes
Source: PLoS One. 2013 May 22;8(5):e63670. doi: 10.1371/journal.pone.0063670 (PMC3661535; doi:10.1371/journal.pone.0063670)
Supplement: Table S2 — Generation of target gene list for the expression screen of metabolism related genes. (PDF) [file pone.0063670.s008.pdf]

Table S2 - Generation of the list of target genes for the expression screen

1. The ChEST collection from MRC GeneService, UK (now known as Source Bioscience) has 1084 plates, with each plate containing 384 different ESTs. Assuming that the chick genome encodes approximately 19,000 genes, the collection provides ~22 fold coverage of the chick genome.

2. ChEST plates #600 – #740 were ordered for this study which would provide ~2.8 fold coverage of all the genes encoded by chick genome.

3. Unigene number (http://www.ncbi.nlm.nih.gov/unigene) was used to filter the ChESTs for unique genes present in these 140 plates.

4. As most of the enzyme names end with "ase", we utilized the text filtering function of Microsoft Excel™ to select the enzymes among the procured ESTs. A detailed description of all the text filters used to generate the target gene list is provided below:

a.	"ase" as the identifier -	2175 ChESTs.
b.	"SUMO" as the identifier -	5 ChESTs
c.	"carrier" as the identifier -	169 ChESTs
d.	"transporter" as the identifier -	101ChESTs
e.	"seleno" as the identifier -	12 ChESTs
f.	"redoxin" as the identifier -	27 ChESTs
g.	"proteasome" as the identifier -	33 ChESTs
h.	"metallo" as the identifier -	16 ChESTs
i.	"selenium" as the identifier -	1 ChESTs
j.	"ROBO" as the identifier -	3 ChESTs
k.	"Thiol" as the identifier -	11 ChESTs
	**Total** -	**2553 ChESTs**

5. 160 gene names were removed from this list which contained "mitochondrial" and "polymerase" in their names.
6. Some gene names were duplicates (i.e. same gene name but two different Unigene numbers) and were eliminated.
7. Final list had **2236** metabolism related genes.
