# Supplementary material for: A Genome-Wide Screen Indicates Correlation between Differentiation and Expression of Metabolism Related Genes
Source: PLoS One. 2013 May 22;8(5):e63670. doi: 10.1371/journal.pone.0063670 (PMC3661535; doi:10.1371/journal.pone.0063670)
Supplement: Table S3 — Many metabolism related genes are expressed in a tissue-restricted manner but not in a tissue-exclusive manner. The number of different embryonic structures/organs a gene is expressed in (Table S3a). The total number of different patterns in which a gene is expressed (Table S3b, all different embryonic structures/organs combined). (PDF) [file pone.0063670.s009.pdf]

	TABLE S3a-Total number of embryonic structures expressing a metabolism related gene	
**S. No.**	**Gene Name**	**Number of embryonic structures/organs with detectable expression**
1	PGAM1	11
2	KDR	11
3	LDHA	10
4	SLC26A8	10
5	CDC42BPB	10
6	DHFR	10
7	NMT1	10
8	QPCT	10
9	YWHAE	10
10	FGGY	9
11	ALDH4A1	9
12	COMT	9
13	FRAS1	9
14	PMPCA	9
15	TAOK1	9
16	TPI1	9
17	PTPN2	9
18	ATP6V0A1	9
19	B3GAT2	9
20	AHSA1	9
21	NDUFA4	8
22	GGT5	8
23	GMPS	8
24	HELLS	8
25	HS6ST2	8
26	NUAK1	8
27	PMM2	8
28	SLC35E2	8
29	WNK2	8
30	PRNP	8
31	ATP2A2	8
32	ACAD9	8
33	ALDH1A2	8

34	G6PC2	8
35	PFKL	8
36	PDIA3	8
37	ABHD12	7
38	CPM	7
39	SLC4A4	7
40	ADSS	7
41	ALDH1A3	7
42	ATP2B1	7
43	COQ2	7
44	DAGLB	7
45	DCI	7
46	GFOD2	7
47	GMPPB	7
48	INPP4A	7
49	LOC769208	7
50	OAZ2	7
51	PPP1R12B	7
52	PTK2	7
53	SENP1	7
54	TOP2B	7
55	PCCA	7
56	GAPDH	7
57	UQCC	7
58	ALDH6A1	7
59	ALDH9A1	7
60	DIO1	7
61	SENP2	7
62	SULT1C3	7
63	ARSJ	6
64	CKB	6
65	B3GALT2	6
66	CBR4	6
67	DPYSL3	6
68	FASN	6
69	FECH	6
70	HAAO	6
71	HSD17B2	6
72	LOC430516	6
73	NKAIN4	6
74	PPP2R4	6
75	PSMD14	6

76	REXO1	6
77	SCP2	6
78	SLC35D1	6
79	SLC44A5	6
80	SQRDL	6
81	SLC7A5	6
82	LIPG	6
83	ARHGAP28	6
84	LOC769421	6
85	SNF1LK	6
86	BPGM	6
87	MAN1A1	6
88	NDUFA12	6
89	NDUFS5	6
90	PRKAB1	6
91	CDKN2B	6
92	CHDH	6
93	CHST15	6
94	GALNT14	6
95	PAFAH2	6
96	PLSCR1	6
97	SLC19A1	6
98	ADAMTS7	6
99	PPP2R2C	6
100	SLC25A25	6
101	PSMA2	6
102	RCL1	6
103	PTK7	6
104	HS3ST3A1	5
105	AASDHPPT	5
106	AGPAT5	5
107	ATAD3A	5
108	B3GNT7	5
109	BCAT1	5
110	LOC771974	5
111	MOXD1	5
112	MUSK	5
113	PDIA4	5
114	PDK3	5
115	PTGES2	5
116	RND3	5
117	SLC39A11	5

118	SLC41A1	5
119	PRCP	5
120	TEK	5
121	BDH1	5
122	ARHGAP12	5
123	Finished_cDNA_clone_ChEST 732p13	5
124	LOC776060	5
125	UST	5
126	THEM2	5
127	ATP6V0E1	5
128	ATP6V1A	5
129	CASK	5
130	GOT1	5
131	ST6GAL1	5
132	USP13	5
133	POR	5
134	TXN2	5
135	ASAH1	5
136	ASNeural Structure	5
137	FBP1	5
138	FMO4	5
139	GALNT1	5
140	HADHA	5
141	LOC768721	5
142	MAP2K1	5
143	PXK	5
144	SLC16A6	5
145	STYK1	5
146	SULT1B1	5
147	DIO3	5
148	ANAPC4	5
149	PLOD1	5
150	CCDC81	5
151	GAL3ST1	5
152	PREPL	5
153	HDAC3	5
154	JAKMIP2	5
155	ADH5	5
156	ALG3	5

	**TABLE S3b-Total number of different expression patterns for metabolism related genes**	
**S. No.**	**Gene Name**	**Total number of patterns in different embryonic structures/organs with detectable expression**
1	PGAM1	20
2	DHFR	19
3	SLC26A8	17
4	QPCT	17
5	LDHA	16
6	ATP2B1	15
7	CDC42BPB	15
8	HS6ST2	15
9	WNK2	15
10	YWHAE	15
11	FGGY	15
12	ALDH1A2	15
13	HELLS	14
14	PMPCA	14
15	TAOK1	14
16	TOP2B	14
17	UQCC	14
18	NMT1	13
19	PTK2	13
20	ARHGAP28	13
21	ABHD12	13
22	ALDH9A1	13
23	ANAPC4	13
24	ADSS	12
25	COMT	12
26	GFOD2	12
27	GGT5	12
28	GMPS	12
29	KDR	12
30	PMM2	12
31	TPI1	12
32	PTPN2	12

33	SNF1LK	12
34	ACAD9	12
35	ATP6V0A1	12
36	CASK	12
37	PFKL	12
38	ARSJ	11
39	AGPAT5	11
40	ALDH1A3	11
41	COQ2	11
42	FRAS1	11
43	GMPPB	11
44	INPP4A	11
45	NUAK1	11
46	SQRDL	11
47	ATP2A2	11
48	B3GAT2	11
49	G6PC2	11
50	ADAMTS7	11
51	SULT1C3	11
52	DAGLB	10
53	FASN	10
54	LOC430516	10
55	NKAIN4	10
56	REXO1	10
57	RND3	10
58	BDH1	10
59	MAN1A1	10
60	POR	10
61	ASAH1	10
62	DIO1	10
63	GALNT1	10
64	HADHA	10
65	PLSCR1	10
66	PPP2R2C	10
