# Supplementary material for: A Genome-Wide Screen Indicates Correlation between Differentiation and Expression of Metabolism Related Genes
Source: PLoS One. 2013 May 22;8(5):e63670. doi: 10.1371/journal.pone.0063670 (PMC3661535; doi:10.1371/journal.pone.0063670)
Supplement: Table S4 — The house-keeping genes that exhibit tissue-specific expression are likely to have specialized functions. List of genes belonging to glycolysis/gluconeogenesis (Table S4a), TCA cycle (Table S4b) and fatty acid metabolism (Table S4c) pathways. The tissue in which these genes are expressed as well as the associated disease names are provided in adjacent columns. (PDF) [file pone.0063670.s010.pdf]

		**TABLE S4a- List of metabolism related genes belonging to glycolysis/gluconeogenesis**			
**S. No.**	**Gene Name**	**In-situ done**	**Expression**	**Structure in which expression was detected**	**Disease Phenotype**
1	HK1				
2	HK2	Y			
3	HKDC1				
4	GCK				
5	GPI	Y			
6	PFKL	Y	Y	Kidney, Liver, Eye, Rhombic Lip, Somite, Otic Vesicle, Neural Tube, Heart	Hemolytic anemia due to phosphofructokinase deficiency, GLYCOGEN STORAGE DISEASE VII
7	PFKM				
8	FBP2				
9	FBP1	Y	Y	Kidney, Liver, Limb	Fructose-1,6-bidphosphatase deficiency,Fructose-bisphosphatase deficiency,
10	ALDOB	Y			
11	ALDOC				

12	TPI1	Y	Y	Eye, Branchial Arches, Heart, Liver, Kidney, Limb, Gut, Frontonasal Primordia	Hemolytic anemia due to triosephosphate isomerase deficiency,
13	GAPDH	Y	Y	Ubiquitous	
14	PGK1				
15	PGAM1	Y	Y	Gut, Kidney, Liver, Limb, Neural tube, Somite, Heart, Frontonasal Primordia	
16	ENO1	Y			
17	ENO2				
18	ENO3				
19	PKM2	Y			
20	PDHA1	Y	Y	Somites	Leigh syndrome, X-linked,pyruvate decarboxylase deficiency,Pyruvate dehydrogenase deficiency,
21	PDHB	Y			
22	DLAT				
23	DLD	Y			
24	LDHA	Y	Y	Branchial Arches, Limb, Frontonasal Primordia, Heart, Kidney, Liver, Eye, Neural Structure, Somite, Gut,	Exertional myoglobinuria due to deficiency of LDH-A,
25	LDHB	Y			
26	ADH1B				
27	ADH5	Y	Y	Somite, Liver, Kidney, Gut, Limb	
28	AKR1A1	Y			
29	ALDH3A2	Y			
30	ALDH2				

31	ALDH7A1	Y			
32	ALDH9A1				
33	ALDH3B1				
34	ALDH1A3	Y	Y	Branchial Arches, Frontonasal Primordia, Limb, Otic Vesicle, Eye, Somite, Kidney,	
35	ACSS1	Y	Y	Limb, Gut, Liver	
36	ACSS2	Y			
37	GALM				
38	PGM1	Y			
39	PGM2				
40	ADPGK				
41	BPGM	Y	Y	Heart, Eye, Otic Vesicle, Limb, Somite, Neural Structure,	Hemolytic anemia due to bisphosphoglycerate mutase deficiency,
42	PCK1				
43	PCK2				
	**25 UG/ 43 GII**	**19 UG/24 GII**	**11 UG/11 GII**		
	**Notes:**				
	**UGs**	**Unique genes**			
	**GII**	**Genes including isoforms**			

		TABLE S4b- List of metabolism related genes belonging to TCA cycle			
S. No.	Gene Name	In-situ done	Expression	Structure in which expression was detected	Disease Phenotype
1	ACLY	y			
2	ACO1				
3	ACO2				
4	CITRATE SYNTHASE				
5	DLAT				
6	DLD	y			
7	DLST				
8	FH	y	y	Notochord, Kidney	ENCEPHALOPATHY, RENAL CANCER
9	IDH1	y	y	Limb, Kidney, Gut tube	Ollier disease and Maffucci syndrome are caused by somatic mosaic mutations of IDH1 and IDH2, Cancer
10	IDH2				
11	IDH3A	y	y		
12	IDH3B				
13	MDH1	y			
14	MDH2				
15	OGDH				
16	OGDHL	y			
17	PCK1				
18	PCK2				
19	PCX				
20	PDHA1	y	y	Somite, Limb	X-linked Leigh syndrome (X-LS) [MIM:308930]

21	PDHB	y			
22	SDHA	y			
23	SDHB				
24	SDHD	y			
25	succinate dehydrogenase [ubiquinone] iron-sulfur subunit				
26	succinate dehydrogenase cytochrome b560 subunit				
27	SUCLG1	y			
28	SUCLG2	y			
	18 UGs/28 GIIs	10 UGs/13 GIIs	4UGs/4G IIs		
	Notes:				
	UGs	Unique genes			
	GII	Genes including isoforms			

		TABLE S4c- List of metabolism related genes belonging to fatty acid metabolism			
S. No.	Gene Name	In-situ done	Expression	Structure in which expression was detected	Disease Phenotype
1	ACAA1	Y	Y	Kidney	Pseudo-Zellweger syndrome
2	ACAA2				
3	ACADL	Y			
4	ACADS	Y			
5	ACADSB				
6	ACAT1				
7	ACAT2	Y			
8	ACOX1				
9	ACOX3	Y			
10	ACSBG1				
11	ACSBG2				
12	ACSL1	Y	Y	Kidney and Limb	
13	ACSL3	Y			
14	ACSL4	Y	Y	Limb	Mental retardation, X-linked 68,Mental retardation, X-linked nonspecific, 63
15	ACSL5				
16	ACSL6				
17	ADH1B				
18	ADH5	Y	Y	Somites, Kidney, Gut tube, Limb	
19	ALDH2				
20	ALDH3A2	Y			
21	ALDH7A1	Y			
22	CPT1A				
23	CPT2	Y	Y	Liver, Kidney	CPT deficiency, hepatic, type II,CPT II deficiency, lethal neonatal,Myopathy due to CPT II deficiency,

24	ECHS1				
25	ECI1				
26	ECI2				
27	EHHADH	Y			
28	YL-CoA DEHYDROGENASE				
29	HADH				
30	HADHA	Y	Y	Eye, Kidney, Liver, limb, Frontonasal Primordia, Gut	
31	HADHB	Y	Y	Limb, Heart, Somites, Kidney	
	**15 UGs/31 GIIs**	**11 UGs/15 GIIs**	**5 UGs/7 GIIs**		
	**Notes:**				
	**UGs**	**Unique genes**			
	**GII**	**Genes including isoforms**			

