# Supplementary material for: A Genome-Wide Screen Indicates Correlation between Differentiation and Expression of Metabolism Related Genes
Source: PLoS One. 2013 May 22;8(5):e63670. doi: 10.1371/journal.pone.0063670 (PMC3661535; doi:10.1371/journal.pone.0063670)
Supplement: Table S5 — Metabolism related genes with conserved expression domains across vertebrate species are associated with diseases wherein the affected tissue is the one expressing the gene. Column “G” lists the embryonic structures in which expression of a gene was detected. Column “H” has the image file ID for the gene’s expression pattern in Mouse or Zebrafish. Column “H” has an entry if the expression of the gene is conserved in Zebrafish or mouse and in one of the embryonic structures whose adult counterpart is affected in a disease. Column “I” has the image file ID for the gene’s expression pattern in Mouse or Zebrafish. Column “I” has an entry if the conserved structure in which expression is observed in chick/mouse/fish is not the one associated with a disease listed in OMIM database. (PDF) [file pone.0063670.s011.pdf]

			TABLE S5- Metabolism related genes with conserved expression domains across vertebrate species and related to disease					
Column A	Column B	Column C	Column D	Column E	Column F	Column G	Column H	Column I
**S. No.**	**Gene symbol**	**Full gene Name**	**OMIM_DISEASE**	**OMIM Phenotype ID**	**Organ/tissue(s) affected in the disease (or Clinical Features)**	**Expression Observed in our Study**	**Conservation of expression in the disease affecetd organ/tissue(s) in Zebrafish and Mouse**	**Conservation of expression in a tissue other than the disease affecetd tissue in zebrafish and mouse**
1	Ace	angiotensin I converting enzyme (peptidyl-dipeptidase A) 1	Alzheimer disease, susceptibility to,Angiotensin I-converting enzyme, benign serum increase,Diabetic nephropathy, susceptibility [illegible]	267430	Renal tubular dysgenesis	Kidney	MGI:4822915, GUDMAP:13511	
2	Acsm3	acyl-CoA synthetase medium-chain family member 3	?Hypertension, essential,		high blood pressure, low renal blood flow	Kidney		
3	Adh1b	alcohol dehydrogenase 1B (class I), beta polypeptide; alcohol dehydrogenase 1A (class I), alpha polypeptide; alcohol dehydrogenase 1C (class I), gamma polypeptide	Alcoholism, susceptibility to,Does parental expressed emotion moderate genetic effects in ADHD? An exploration using a genome wide association scan,Parkinson disease, susceptibility to,	103780	Alcohol dependence, cardiomyopathy, pancreatitis	Liver, Kidney		
4	Aldh5a1	aldehyde dehydrogenase 5 family, member A1	Succinic semialdehyde dehydrogenase deficiency,	271980	neurologic abnormalities, mild mental retardation, urinary excretion of gamma-hydroxybutyric acid	Heart, Kidney, Gut Tube, Liver		
5	Aldh6a1	aldehyde dehydrogenase 6 family, member A1	Methylmalonate semialdehyde dehydrogenase deficiency,	614105	elevated beta-alanine, 3-hydroxypropionic acid, and both isomers of 3-amino and 3-hydroxyisobutyric acids in urine organic acids	Liver, Kidney, Gut Tube, Heart, Hind limb, Fore limb	MGI:5341629, ZFIN ID: ZDB-ANAT-010921-585	
6	Alg3	asparagine-linked glycosylation 3, alpha-1,3-mannosyltransferase homolog (S. cerevisiae)	Congenital disorder of glycosylation, type Id,	601110	severe psychomotor handicap, multiple dysmorphisms including microcephaly, dysplastic ears, atrophy of the optic nerve, and coloboma of the iris, clubfeet and contractures of the hands, severe digestive issues	Gut Tube, Heart, Fore limb, Hind limb, Liver, Somite	MGI:5328586	
7	Alg6	asparagine-linked glycosylation 6, alpha-1,3-glucosyltransferase homolog (S. cerevisiae)	Congenital disorder of glycosylation, type Ic,	603147	psychomotor retardation, muscular hypotonia, distal limb defects, atrophic retinal pigmentation, gastrointestinal issues	Forelimb, Hindlimb, Somite		
8	Arsb	arylsulfatase B	Maroteaux-Lamy syndrome, several forms,Variants in TF and HFE explain approximately 40% of genetic variation in serum-transferrin levels,	253200	stiff joints, corneal clouding, cardiac abnormalities, facial dysmorphism, increased urinary excretion of chondroitin sulfate, bony destruction in the shoulders, hips, and skull, neurologic deterioration	Heart, Fore limb, Hind limb, Kidney, Somite	MGI:4509735, MGI:5329137	
9	Asah1	N-acylsphingosine amidohydrolase (acid ceramidase) 1	Farber lipogranulomatosis,	228000	motor and mental retardation, arthritis, infiltration of liver, spleen, lungs, thymus, elevated urine ceramide levels, painful swollen joints	Fore limb, Hind limb, Gut Tube, Kidney, Liver, Neural Tube	MGI:5322527, MGI:5322529, MGI:4896167	
10	Cask	calcium/calmodulin-dependent serine protein kinase (MAGUK family)	FG syndrome 4,Mental retardation and microcephaly with pontine and cerebellar hypoplasia,	300422	FG syndrome 4, Mental retardation	Forelimb, Hindlimb, Heart, Neural Tube, Gut Tube, Tail	MGI:4529333, MGI:5341543, MGI:5341550, ZDB-IMAGE-050309-828	
11	Ces1	carboxylesterase 1 (monocyte/macrophage serine esterase 1)	Carboxylesterase 1 deficiency		deficiency of drug metabolism and detoxication of harmful chemicals	Forelimb, Hindlimb, Liver, Kidney	MGI:5324525	
12	Cilp	cartilage intermediate layer protein, nucleotide pyrophosphohydrolase	Lumbar disc disease, susceptibility to,	603932	degeneration of intervertebral discs of the lumbar spine, musculoskeletal disorders	Hindlimb, Forelimb, Somite		

13	Col3a1	collagen, type III, alpha 1	Aneurysm, familial arterial,Ehlers-Danlos syndrome, type III,Ehlers-Danlos syndrome, type IV,	130020	Ehlers-Danlos syndrome, type III, Marked joint hyperextensibility without skeletal deformity, aortic root dilatation, neuromuscular symptoms, muscle weakness	Ubiquitous	MGI:3588096, MGI:4500651	
14	Comt	catechol-O-methyltransferase	Panic disorder, susceptibility to,Schizophrenia, susceptibility to,	167870	Panic disorder, panic attacks, cardiac palpitations, anxiety or depression	Branchial Arches, Eye, Heart, Otic Vesicle, Somite, Liver, Forelimb, Hindlimb, Mandible, Rhombic Lip	MGI:5323753	MGI:5335599
15	Cpt2	carnitine palmitoyltransferase 2	CPT deficiency, hepatic, type II,CPT II deficiency, lethal neonatal,Myopathy due to CPT II deficiency,	600649	CPT deficiency, hepatic, type II	Kidney, Liver	MGI:5340141, ZFIN ID: ZDB-FIG-120409-3	
16	Dpys	dihydropyrimidinase	Dihydropyrimidinuria,	222748	neurologic abnormalities, urinary dihydrouracil, club foot and hip dysplasia, liver cirrhosis	Kidney, Liver, Eye		
17	Fa2h	fatty acid 2-hydroxylase	Leukodystrophy, dysmyelinating, and spastic paraparesis with or without dystonia,	612319	neurologic abnormalities such as dystonia, optic atrophy, and seizures, increased muscle tone	Notochord		
18	Fbp1	fructose-1,6-bisphosphatase 1	Fructose-1,6-bidphosphatase deficiency,Fructose-bisphosphatase deficiency,	229700	Hepatic fructose-1,6-bisphosphatase deficiency	Kidney, Liver, Forelimb, Hindlimb, Rhombomere, Gut Tube	ZFIN ID: ZDB-FIG-090818-2	GUDMAP:9181
19	Fech	ferrochelatase (protoporphyria)	Protoporphyria, erythropoietic,Protoporphyria, erythropoietic, autosomal dominant,Protoporphyria, erythropoietic, autosomal recessive,Protoporphyria, erythropoietic, recessive, with liver failure,	177000	Chronic skin changes, severe polyneuropathy, painful photosensitivity, fatal liver damage, gallstones at a relatively young age	Branchial Arches, Heart, Otic Vesicle, Forelimb, Hindlimb, Kidney, Liver, Somite	MGI:5323664	MGI:5323665
20	Fh	fumarate hydratase	Fumarase deficiency,Leiomyomatosis and renal cell cancer,Multiple cutaneous and uterine leiomyomata,	606812	Fumarase deficiency, profound psychomotor retardation, and brain abnormalities, increased urinary excretion to a defect in renal clearance, Marked deficiency of both mitochondrial and cytosolic fumarases was found in skeletal muscle, brain, cerebellum, heart, kidney, liver,	Kidney, Liver, Eye, Gut Tube	MGI:5337094, MGI:5337087	ZFIN ID: ZDB-FIG-080225-138, MGI:5337085 , MGI:5337088
21	Fras1	Fraser syndrome 1	Fraser syndrome,	219000	middle and outer ear malformations, syndactyly, maldeveloped kidneys	Forelimb, Hindlimb, Gut Tube, Somite, Kidney	MGI:3692313	MGI:4538137
22	Fuca1	fucosidase, alpha-L- 1, tissue	Fucosidosis,	230000	psychomotor retardation, deficiency of alpha-fucosidase activity in the liver	Forebrain, Tail	MGI:5328345	
23	Hadha	hydroxyacyl-Coenzyme A dehydrogenase/3-ketoacyl-Coenzyme A thiolase/enoyl-Coenzyme A hydratase (trifunctional protein), alpha subunit	Fatty liver, acute, of pregnancy,HELLP syndrome, maternal, of pregnancy,LCHAD deficiency,Trifunctional protein deficiency,Trifunctional protein deficiency, type 1,	609016	Fatty liver, HELLP syndrome(hemolysis, elevated liver enzymes, and low platelets), hypertension, LCHAD deficiency, cardiomyopathy, hypoglycemia, neuropathy, myopathy and pigmentary retinopathy	Eye, Kidney, Forelimb, Hindlimb, Liver, Gut Tube, Nasal Primordia	MGI:5339512, MGI:5339514, ZFIN ID: ZDB-FIG-120409-3	
24	Hadhb	hydroxyacyl-Coenzyme A dehydrogenase/3-ketoacyl-Coenzyme A thiolase/enoyl-Coenzyme A hydratase (trifunctional protein), beta subunit	Trifunctional protein deficiency,Trifunctional protein deficiency, type II,	609015	Trifunctional protein deficiency, respiratory failure, poor spontaneous motility, acute cardiac failure, muscle weakness	Lateral Plate Mesoderm, Somite, Heart, Kidney, Hindlimb	MGI:2669361	
25	Hal	histidine ammonia-lyase	Histidinemia,	235800	mental retardation and speech defects, increased histidine in blood, urine, and cerebrospinal fluid, and decreased urocanic acid in blood, urine, and skin cells	Liver	MGI:5327118	
26	Idh3b	isocitrate dehydrogenase 3 (NAD+) beta	Retinitis pigmentosa-46,	612572	subnormal visual acuities, reduced muscle strength, cardiac dysrhythmias, or reduced athletic stamina	Liver, Gut Tube, Heart, Kidney, Limb	MGI:5338983, MGI:5338984	

27	Kdr	kinase insert domain receptor (a type III receptor tyrosine kinase)	Hemangioma, capillary infantile, somatic,Hemangioma, capillary infantile, susceptibility to,	602089	vascular malformations	Eye, Heart, Somite, Kidney, Fore limb, Hind limb, Gonad, Liver, Rhombic Lip, Vasculature	ZFIN ID: ZDB-FIG-090623-2 , MGI:1334303	ZFIN ID: ZDB-FIG-100503-2
28	Musk	muscle, skeletal, receptor tyrosine kinase	Myasthenic syndrome, congenital, associated with acetylcholine receptor deficiency,	608931	Ophthalmoparesis,Facial muscle weakness , Muscle cramps , Generalized muscle weakness due to defect at the neuromuscular junction Underdeveloped muscles	otic vesicle, branchial arches, somite, fore limb, whole embryo	ZFIN ID: ZDB-FIG-050810-257, MGI:4566078	
29	Ndp	Norrie disease (pseudoglioma)	Exudative vitreoretinopathy, X-linked,Norrie disease,	310600	, Intraocular retrolental masses, bilateral ('pseudoglioma'), Mental retardation, progressive (50% of patients) Dementia (later onset) Seizures (rare),	Rhombic limb, rhombomeres, neural tube		
30	Ndufv1	NADH dehydrogenase (ubiquinone) flavoprotein 1, 51kDa	Alexander disease,Leigh syndrome,Mitochondrial complex I deficiency,	252010	Macrocephaly, Muscle weakness Muscle atrophy , Developmental delay , Pale optic disks	Whole embryo, Rhombic lip, somite	MGI:5324477	
31	Ntrk2	neurotrophic tyrosine kinase, receptor, type 2	Obesity, hyperphagia, and developmental delay,	613886	severe developmental delay in motor function, speech, and language, and demonstrated a blunted response to nociceptive stimuli.	Forebrain, ganglia, Rhombic lip, liver, nasal primordia, neural tube	MGI:3830106, ZFIN ID: ZDB-FIG-080327-393	
32	Oat	ornithine aminotransferase (gyrate atrophy)	Gyrate atrophy of choroid and retina with ornithinemia, B6 responsive or unresponsive,	258870	Elevated urinary ornithine, lysine, arginine, Tubular aggregates in type 2 skeletal muscle fibers, Progressive chorioretinal degeneration	kidney, liver, gut tube, nasal primordia, limb	MGI:5323958,	MGI:5323957, MGI:5323957
33	Oxct1	3-oxoacid CoA transferase 1	Succinyl CoA:3-oxoacid CoA transferase deficiency,3-HYDROXYACYL-CoA DEHYDROGENASE DEFICIENCY	231530	Hepatic necrosis, Hypotonia , Dilated cardiomyopathy, Dicarboxylic aciduria	heart, somite, limb, kidney		ZFIN ID: ZDB-FIG-050630-4619, MGI:3784452
34	Papss2	3'-phosphoadenosine 5'-phosphosulfate synthase 2	SEMD, Pakistani type,	612847	Platyspondyly, Irregular end plates , Short and bowed lower limb, Brachydactyly, mild, Hyperandrogenism	limb, otic visicle, kidney	MGI:3689699, MGI:4508908	ZFIN ID: ZDB-FIG-050630-7920
35	Pcca	propionyl Coenzyme A carboxylase, alpha polypeptide	Propionicacidemia,	606054	Cardiomyopathy, Hepatomegaly , Pancreatitis, Acute encephalopathy , Cerebral atrophy , Anemia	eye, gut, liver, kidney, limb	**ZFIN ID:** ZDB-FIG-111117-1, MGI:5329195	MGI:5329187, ZFIN ID: ZDB-FIG-050630-5967
36	Pfkl	phosphofructokinase, liver	Hemolytic anemia due to phosphofructokinase deficiency, GLYCOGEN STORAGE DISEASE VII	232800	Gallstones due to hemolytic anemia, Jaundice , Muscle weakness, Hyperuricemia, Increased reticulocyte count, Gout due to increased uric acid	eye, heart, kidney, liver, whole embryo, limb, neural tube	MGI:2666892, MGI:5325923	MGI:5325919
37	Plod1	procollagen-lysine 1, 2-oxoglutarate 5-dioxygenase 1	Ehlers-Danlos syndrome, type VI,Nevo syndrome,	225400	Retinal detachment, Glaucoma, Depressed nasal bridge, Tooth crowding, Cardiac failure, Recurrent episodes of pneumonia,Gastrointestinal hemorrhage , Osteoporosis , Delayed motor development	eye, rhombomeres, somite, limb, neural tube, rhombic lip,	MGI:5324678,	ZFIN ID: ZDB-FIG-070326-1
38	Pmm22	phosphomannomutase 2	Carbohydrate-deficient glycoprotein syndrome, type I,	212065	Large ears, Abnormal eye movements , Flat nasal bridge , Thin upper lip , Diarrhea , Osteopenia , Hypotonia , Peripheral neuropathy	Branchial arches, eye, limb,kidney, liver, otic vesicle	**ZFIN ID:** ZDB-FIG-050630-11968	
39	Por	P450 (cytochrome) oxidoreductase	Adrenal hyperplasia, congenital, due to combined P450C17 and P450C21 deficiency,Antley-Bixler syndrome-like with disordered steroidogenesis,Disordered steroidogenesis, isolated,POR deficiency,	201750	Brachycephaly, Hearing loss, Proptosis in eyes, Depressed nasal bridge , Micropenis , Hypoplastic labia, Cloverleaf skull , Femoral fractures ,Rocker-bottom feet , Horseshoe kidney	limb, kidney, neural tube, rhombic lip		

40	Prnp	prion protein	Creutzfeldt-Jakob disease,Gerstmann-Straussler disease,Huntington disease-like 1,Insomnia, fatal familial,Prion disease with protracted course,	137440	progressive limb and truncal ataxia, dysarthria, Cerebellar atrophy	kidney, limb,gut, somite,branchial arches		GUDMAP:11175, MGI:2153603
41	Serpinh1	serpin peptidase inhibitor, clade H (heat shock protein 47), member 1, (collagen binding protein 1)	Preterm premature rupture of the membranes, OSTEOGENESIS IMPERFECTA, TYPE X	613848	Macrocephaly, Shallow orbits , Chronic lung disease , Pyloric stenosis , Bone fractures, Short limbs, Hypotonia	somite, limb, kidney	MGI:3042602	
42	Slc1a3	solute carrier family 1 (glial high affinity glutamate transporter), member 3	Episodic ataxia, type 6,	108500	Ocular abnormalities, Atrophy of cerebellar vermis, Migraine headache	Rhombomeres, somite, neural tube, rhombic lip,	MGI:3843269, ZFIN ID: ZDB-FIG-050630-1161, ZFIN ID: ZDB-FIG-100407-4	
43	Slc26a2	solute carrier family 26 (sulfate transporter), member 2	Achondrogenesis Ib,Atelosteogenesis II,De la Chapelle dysplasia,Diastrophic dysplasia,Diastrophic dysplasia, broad bone-platyspondylic variant,Epiphyseal dysplasia, multiple, 4,	222600	Hearing loss, Cleft palate, Short, thick tubular bone, Spinal cord compression ,Short finger with ulnar deviation	limb	MGI:4881818	
44	Slc35d1	solute carrier family 35 (UDP-glucuronic acid/UDP-N-acetylgalactosamine dual transporter), member D1	Schneckenbecken dysplasia,	269250	Large head, Brachydactyly , Precociously ossified carpal bones , Metaphyseal irregularities	branchial arches,eye,limb, liver, whole embryo		ZFIN ID: ZDB-FIG-080225-318
45	Slc39a13	solute carrier family 39 (zinc transporter), member 13	Spondylocheirodysplasia, Ehlers-Danlos syndrome-like,	612350	Protuberant eyes,Joint laxity, Platyspondyly , Thenar muscle atrophy	limb, somite	**ZFIN ID:** ZDB-FIG-060317-1	
46	Slc4a4	solute carrier family 4, sodium bicarbonate cotransporter, member 4	Renal tubular acidosis, proximal, with ocular abnormalities,	604278	cataract, corneal opacities, Proximal renal tubular acidosis , Mental retardation ,	hind limb, branchial arches,limb, neural tube, otic vesicle,eye, whole embryo	**ZFIN ID:** ZDB-FIG-110216-7, MGI:4580295	ZFIN ID: ZDB-FIG-091030-2
47	Tek	TEK tyrosine kinase, endothelial	Venous malformations, multiple cutaneous and mucosal,	600195	arteriovenous malformations, and capillary hemangiomas, Lesions in arms and legs, face, oral mucosa, or genitalia, vascular tumors within the stomach, liver, pancreas, gastrointestinal, renal, and brain lesions	somite,heart,kidney,forelimb, hind limb	MGI:1310020, GUDMAP:11333	
48	Tmprss6	transmembrane protease, serine 6	Iron-refractory iron deficiency anemia,Variants in TF and HFE explain approximately 40% of genetic variation in serum-transferrin levels,	206200	GI(iron malabsorption), anemia	whole embryo, liver, gut, otic vesicle		
49	Tpi1	TPI1 pseudogene; triosephosphate isomerase 1	Hemolytic anemia due to triosephosphate isomerase deficiency,	MIM no. not ther	Degenerative neurologic disorder with spasticity, Jaundice , Cardiac failure , Myopathy , Cholecystitis in gut	Branchial arches, eye, heart, limb,kidney, liver, gut	ZFIN ID: ZDB-FIG-050630-7445, MGI:3770881,	MGI:3770881
50	Tsen54	tRNA splicing endonuclease 54 homolog (S. cerevisiae)	Pontocerebellar hypoplasia type 2A,Pontocerebellar hypoplasia type 4,	2,25,753	Swallowing disturbances, Hypertonia, Delayed psychomotor development, Brainstem hypoplasia	forelimb, hindlimb,gut tube		ZFIN ID: ZDB-FIG-110425-6
51	Urod	uroporphyrinogen decarboxylase	Porphyria cutanea tarda,Porphyria, hepatoerythropoietic,	176100	Hepatic hemosiderosis , Hepatic cirrhosis ,hepatocellular carcinoma ,	limb, kidney, somite	MGI:5328431	MGI:5328432, MGI:5328442 , MGI:5328436
52	Xdh	xanthine dehydrogenase	Xanthinuria, type I,	278300	Myopathy, Hydronephrosis, Xanthinuria, Crystalline deposits in skeletal muscle	kidney, gut tube	kidney,	
53	Ywhae	similar to 14-3-3 protein epsilon (14-3-3E) (Mitochondrial import stimulation factor L subunit) (MSF L); tyrosine 3-monooxygenase/tryptophan 5-monooxygenase activation protein, epsilon polypeptide	Miller-Dieker lissencephaly,	247200	Microcephaly,Furrowing of forehead, Posteriorly rotated ears , Cataract , Congenital heart defect , Duodenal atresia , Cystic kidney , Polydactyly , Mental retardation	eye, heart, kidney, otic vesicles, branchial arches, forelimb, rhombic lip	MGI:2683200, ZFIN ID: ZDB-FIG-070314-28 , MGI:5341303	
54	Ldha	lactate dehydrogenase A	Exertional myoglobinuria due to deficiency of LDH-A,	612933	,Renal failure may occur, Muscle cramps Muscle pain Muscle stiffness	branchial arches, eye, forelimb,neural tube, heart, gut,kidney, hind limb	MGI:5341137	MGI:5341136, MGI:5341142, MGI:5341142, MGI:5341135
55	Acaa1	acetyl-Coenzyme A acyltransferase 1	Pseudo-Zellweger syndrome,			Kidney, Liver, Limb		MGI:5324737,MGI:5324733 , MGI:5324736, ZFIN ID: ZDB-FIG-080326-2

56	Acsl4	acyl-CoA synthetase long-chain family member 4	Mental retardation, X-linked 68,Mental retardation, X-linked nonspecific, 63,	300387	mental retardation	Hind Limb, Fore Limb		MGI:5342101
57	Aldh4a1	aldehyde dehydrogenase 4 family, member A1	Hyperprolinemia, type II,	239510	mild mental retardation, hyperglycinemia	Liver, Hindlimb, Forelimb, Branchial Arches, Heart, Kidney, Gut Tube, Somite		ZFIN ID: ZDB-FIG-050810-494 , MGI:5327595 , MGI:5327599
58	Alox5ap	arachidonate 5-lipoxygenase-activating protein	Myocardial infarction, susceptibility to,Stroke, susceptibility to,	601367	Myocardial infarction (Heart Attack),Stroke	Kidney		
59	Atp2b2	ATPase, Ca++ transporting, plasma membrane 2	Deafness, autosomal recessive 12, modifier of,Genetics Meets Metabolomics: A Genome-Wide Association Study of Metabolite Profiles in Human Serum,	601386	profound hearing loss	Forelimb, Hindlimb, Gut Tube		
60	Bpgm	2,3-bisphosphoglycerate mutase	Hemolytic anemia due to bisphosphoglycerate mutase deficiency,	222800	severe hemolytic anemia	Eye, Heart, Otic Vesicle, Forelimb, Hindlimb, Neural Tube, Somite		
61	Glul	glutamate-ammonia ligase (glutamine synthetase)	Glutamine deficiency, congenital,	610015	encephalopathy, lack of normal development, seizures, shortness of limbs, flexion contractures of elbows and knees, muscular hypotonia	Liver		
62	Gmps	guanine monphosphate synthetase	Leukemia, acute myelogenous,	601626	bilateral soft tissue orbital tumors in bone marrow	Forelimb, Hindlimb, Otic Vesicle, Gut Tube, Eye, Liver, Kidney		ZFIN ID: ZDB-FIG-050630-14734, MGI:5330993
63	Kel	Kell blood group, metallo-endopeptidase	Blood group, Kell,	110900	hemolytic disease of the newborn	Somite, Tail		
64	Lox	lysyl oxidase	Cutis laxa, recessive, type I,	219100	Inguinal hernia , Bladder diverticula, Recurrent respiratory infections	eye, forelimb, hind limb, kidney, gut		MGI:3693476, MGI:5344009, ZFIN ID: ZDB-FIG-080921-1
65	Ltc4s	leukotriene C4 synthase	Leukotriene C4 synthase deficiency,	614037	muscular hypotonia, psychomotor retardation, failure to thrive, and microcephaly	Branchial arches, heart		
66	Maoa	monoamine oxidase A	antisocial behavior,Brunner syndrome,	300615	Nondysmorphic mild mental retardation	Limb, somite, otic visicle, branchial arches		
67	Mpi	mannose phosphate isomerase	Carbohydrate-deficient glycoprotein syndrome, type Ib, **Congenital disorder of glycosylation, type Ib**	602579	Hepatic fibrosis, Cirrhosis , Hyperinsulinemic hypoglycemia	Somite, limb		ZFIN ID: ZDB-FIG-050630-10018,
68	Mthfd1	methylenetetrahydrofolate dehydrogenase (NADP+ dependent) 1, methenyltetrahydrofolate cyclohydrolase, formyltetrahydrofolate synthetase	Abruptio placentae, susceptibility to,Spina bifida, folate-sensitive, susceptibility to,			limb, liver, heart		ZFIN ID: ZDB-FIG-050630-6718, MGI:4502146, MGI:5328787
69	Ndufs4	NADH dehydrogenase (ubiquinone) Fe-S protein 4, 18kDa (NADH-coenzyme Q reductase)	Leigh syndrome,Mitochondrial complex I deficiency,	252010	Macrocephaly, Muscle weakness Muscle atrophy , Developmental delay , Pale optic disks	kidney, limb, interdigital mesenchyme		MGI:5322502
70	Ndufs4	NADH dehydrogenase (ubiquinone) Fe-S protein 4, 18kDa (NADH-coenzyme Q reductase)	Leigh syndrome,Mitochondrial complex I deficiency,	256000	Hypotonia in muscle,Optic atrophy , Psychomotor retardation Hypotonia Ataxia , Increased serum lactate	kidney, limb, interdigital mesenchyme		
71	Pah	phenylalanine hydroxylase	Hyperphenylalaninemia, mild,Hyperphenylalaninemia, non-PKU mild,Phenylketonuria,	261600	Cataracts, Pale pigmentation , Mental retardation, Limb posturing & Brain calcification (if left untreated), Phenylpyruvic acidemia	liver, gut, otic vesicle		ZFIN ID: ZDB-FIG-050630-12000, MGI:4468270, MGI:5324349
72	Pdha1	pyruvate dehydrogenase (lipoamide) alpha 1	Leigh syndrome, X-linked,pyruvate decarboxylase deficiency,Pyruvate dehydrogenase deficiency,	308930	Hypotonia, Neurodegeneration , Nystagmus , Lactic acidosis	somite,heart,kidney,forelimb, hind limb		ZFIN ID: ZDB-FIG-050630-2274, MGI:5323277 , MGI:5323287
73	Prepl	prolyl endopeptidase-like	Homozygous 2p16 deletion syndrome,			kidney, Rhombic lip, Somite, Ganglia, limb, Gut		MGI:4499341
74	Prkch	protein kinase C, eta	Cerebral infarction, susceptibility to,			limb		

75	Prps1	phosphoribosyl pyrophosphate synthetase 1; phosphoribosyl pyrophosphate synthetase 1-like 1	Arts syndrome,Charcot-Marie-Tooth disease, X-linked recessive, 5,Gout, PRPS-related,Phosphoribosylpyrophosphate synthetase superactivity,	614458	Hypotonia, Encephalopathic episodes, Delayed psychomotor development, Left ventricular hypertrophy	somite, forelimb,gut tube, hind limb		ZFIN ID: ZDB-FIG-050630-1505,
76	Qdpr	quinoid dihydropteridine reductase	Hyperphenylalaninemia due to dihydropteridine reductase deficiency,Hyperphenylalaninemia, BH4-deficient, C,Phenylketonuria due to dihydropteridine reductase deficiency,	261630	Hypersalivation, Mental retardation , Swallowing difficulties	kidney		MGI:5328734
77	Scp2	sterol carrier protein 2	Leukoencephalopathy with dystonia and motor neuropathy,			branchial arches, heart, limb, gut, kidney, liver		ZFIN ID: ZDB-FIG-060216-450
78	Slc37a4	solute carrier family 37 (glucose-6-phosphate transporter), member 4	Glycogen storage disease Ib,Glycogen storage disease Ic,			limb, kidney, liver		ZFIN ID: ZDB-FIG-060216-387 , MGI:4581149, MGI:5342633
79	Slc40a1	solute carrier family 40 (iron-regulated transporter), member 1	Hemochromatosis, type 4,			gut tube, kidney, liver		ZFIN ID: ZDB-FIG-050726-1, MGI:4495979, MGI:4495983
80	Sod3	superoxide dismutase 3, extracellular	Superoxide dismutase, elevated extracellular,			forelimb		MGI:4949390
81	Tat	tyrosine aminotransferase	Tyrosinemia, type II,	276600	plaques on palms and soles and linear , star-like corneal opacities, severe mental and somatic retardation, elevated tyrosine blood levels, increase in urinary p-hydroxyphenylpyruvate	otic vesicle,branchial arches, limb,beak primordia		
82	Ubiad1	UbiA prenyltransferase domain containing 1	Corneal dystrophy, crystalline, of Schnyder,Corneal dystrophy, crystalline, Schnyder,		eye	kidney,somite		
