# Supplementary material for: A Genome-Wide Screen Indicates Correlation between Differentiation and Expression of Metabolism Related Genes
Source: PLoS One. 2013 May 22;8(5):e63670. doi: 10.1371/journal.pone.0063670 (PMC3661535; doi:10.1371/journal.pone.0063670)
Supplement: Table S6 — List of all the Chicken EST clones and full names of the genes used in this study. (PDF) [file pone.0063670.s012.pdf]

	**TABLE S6-List of all the ChEST clones and full name of the genes used in this study**				
**Gene Symbol**	**Gene Name**	**ChEST clone ID**	**UniGene ID**	***In situ done***	**Expression**
AACS	Acetoacetyl-CoA synthetase	ChEST 578d7	Gga.22600		
AADACL2	Arylacetamide deacetylase-like 2	ChEST 624n11	Gga.16268	Y	
AADACL4	Arylacetamide deacetylase-like 4	ChEST 564l2	Gga.40425	Y	Y
AARS	Alanyl-tRNA synthetase	ChEST 612c24	Gga.1138	Y	
AARSD1	Alanyl-tRNA synthetase domain containing 1	ChEST 374p19	Gga.5572	Y	

AASDHPPT	Transcribed locus, strongly similar to NP_061111.1 carbohydrate (chondroitin 4) sulfotransferase 12 [Homo sapiens]	ChEST 660i16	Gga.26664	Y	
ABAT	4-aminobutyrate aminotransferase	ChEST 563o13	Gga.47502	Y	
ABHD12	Abhydrolase domain containing 12	ChEST 387d4	Gga.4670	Y	
ABHD13	Abhydrolase domain containing 13	ChEST 624g15	Gga.42705	Y	
ABHD2	Abhydrolase domain containing 2	ChEST 714j23	Gga.35328		
ABHD3	Abhydrolase domain containing 3	ChEST 628n19	Gga.31810	Y	Y
ABHD5	Abhydrolase domain containing 5	ChEST 612n15	Gga.5454	Y	

ABHD6	Abhydrolase domain containing 6	ChEST 603f18	Gga.8889	Y	
ACAA1	Acetyl-Coenzyme A acyltransferase 1 (peroxisomal 3-oxoacyl-Coenzyme A thiolase)	ChEST 533b24	Gga.7576	Y	Y
ACACA	Acetyl-Coenzyme A carboxylase alpha	ChEST 735k5	Gga.1480	Y	
ACAD10	Acyl-Coenzyme A dehydrogenase family, member 10	ChEST 635i1	Gga.27747	Y	
ACAD11	Acyl-Coenzyme A dehydrogenase family, member 11	ChEST 611k6	Gga.8980	Y	
ACAD8	Acyl-Coenzyme A dehydrogenase family, member 8	ChEST 660i22	Gga.16393	Y	
ACAD9	Acyl-Coenzyme A dehydrogenase family, member 9	ChEST 514a20	Gga.22451	Y	Y

ACADL	Acyl-Coenzyme A dehydrogenase, long chain	ChEST 648j1	Gga.6159	Y	
ACADS	Acyl-Coenzyme A dehydrogenase, C-2 to C-3 short chain	ChEST 622m4	Gga.16136		
ACAT2	Acetyl-Coenzyme A acetyltransferase 2 (acetoacetyl Coenzyme A thiolase)	ChEST 678g23	Gga.20086		
ACE	Angiotensin I converting enzyme (peptidyl-dipeptidase A) 1	ChEST 600a16	Gga.3781	Y	Y
ACLY	ATP citrate lyase	ChEST 586j19	Gga.2159	Y	
ACMSD	Aminocarboxymuconate semialdehyde decarboxylase	ChEST 563c12	Gga.37298	Y	
ACOT11	Acyl-CoA thioesterase 11	ChEST 617k3	Gga.29968		

ACOT7	Acyl-CoA thioesterase 7	ChEST 601g9	Gga.5995	Y	
ACOT8	Acyl-CoA thioesterase 8	ChEST 597b4	Gga.4688		
ACOT9	Acyl-CoA thioesterase 9	ChEST 603g21	Gga.17540	Y	
ACOX2	Acyl-Coenzyme A oxidase 2, branched chain	ChEST 653k21	Gga.29984	Y	
ACOX3	Transcribed locus, weakly similar to NP_060192.1 TRM1 tRNA methyltransferase 1 [Homo sapiens]	ChEST 564j10	Gga.31057	Y	
ACOX3	Acyl-Coenzyme A oxidase 3, pristanoyl	ChEST 691a7	Gga.31056	Y	
ACP1	Acid phosphatase 1, soluble	ChEST 629k19	Gga.23765		

ACP6	Acid phosphatase 6, lysophosphatidic	ChEST 649n4	Gga.12269	Y	
ACPL2	Acid phosphatase-like 2	ChEST 634a3	Gga.36542	Y	
ACSBG1	Acyl-CoA synthetase bubblegum family member 1	ChEST 740g23	Gga.12240	Y	
ACSF2	Acyl-CoA synthetase family member 2	ChEST 592k3	Gga.31150	Y	Y
ACSL1	Acyl-CoA synthetase long-chain family member 1	ChEST 606d23	Gga.18942	Y	Y
ACSL3	Acyl-CoA synthetase long-chain family member 3	ChEST 624m1	Gga.31297	Y	
ACSL4	Acyl-CoA synthetase long-chain family member 4	ChEST 586d16	Gga.42258	Y	Y

ACSM3	Acyl-CoA synthetase medium-chain family member 3	ChEST 731k20	Gga.7804	Y	Y
ACSM5	Acyl-CoA synthetase medium-chain family member 5	ChEST 728m24	Gga.13676	Y	
ACSS1	Acyl-CoA synthetase short-chain family member 1	ChEST 631c9	Gga.7505	Y	Y
ACSS2	Acyl-CoA synthetase short-chain family member 2	ChEST 698a17	Gga.30557	Y	
ACSS3	Acyl-CoA synthetase short-chain family member 3	ChEST 731h13	Gga.14472		
ACY1L2	Aminoacylase 1-like 2	ChEST 729l19	Gga.21465	Y	
ACYP1	Acylphosphatase 1, erythrocyte (common) type	ChEST 605h11	Gga.4647	Y	

ACYP2	Acylphosphatase 2, muscle type	ChEST 533i18	Gga.5281	Y	
ADA	Adenosine deaminase	ChEST 374i16	Gga.9054	Y	
ADA	Transcribed locus, strongly similar to XP_001516783.1 PREDICTED: similar to (N6-adenosine)-methyltransferase, partial [Ornithorhynchus anatinus]	ChEST 675k6	Gga.9056		
ADAD1	Adenosine deaminase domain containing 1 (testis-specific)	ChEST 720k10	Gga.34392	Y	
ADAM10	ADAM metallopeptidase domain 10	ChEST 603a23	Gga.1343		
ADAM17	ADAM metallopeptidase domain 17 (tumor necrosis factor, alpha, converting enzyme)	ChEST 563k10	Gga.7793	Y	Y
ADAM19	ADAM metallopeptidase domain 19 (meltrin beta)	ChEST 673n14	Gga.24894		

ADAM20	ADAM metallopeptidase domain 20	ChEST 603l16	Gga.39871	Y	
ADAM23	Transcribed locus, strongly similar to XP_419804.1 PREDICTED: similar to Prenyl (decaprenyl) diphosphate synthase, subunit 2 [Gallus gallus]	ChEST 705h20	Gga.43466	Y	
ADAM9	ADAM metallopeptidase domain 9 (meltrin gamma)	ChEST 602i24	Gga.21336		
ADAMTS1	ADAM metallopeptidase with thrombospondin type 1 motif, 1	ChEST 564h13	Gga.29367		
ADAMTS17	ADAM metallopeptidase with thrombospondin type 1 motif, 17	ChEST 652o18	Gga.18545	Y	
ADAMTS19	ADAM metallopeptidase with thrombospondin type 1 motif, 19	ChEST 643g11	Gga.25837	Y	Y
ADAMTS3	ADAM metallopeptidase with thrombospondin type 1 motif, 3	ChEST 649d21	Gga.12813		

ADAMTS6	ADAM metallopeptidase with thrombospondin type 1 motif, 6	ChEST 696c8	Gga.42296	Y	
ADAMTS7	Similar to COMPase	ChEST 719g18	Gga.20141	Y	Y
ADAMTS9	ADAM metallopeptidase with thrombospondin type 1 motif, 9	ChEST 647f11	Gga.39725		
ADAR	Adenosine deaminase, RNA-specific	ChEST 655n10	Gga.8033	Y	
ADARB1	Adenosine deaminase, RNA-specific, B1 (RED1 homolog rat)	ChEST 600e3	Gga.2137	Y	
ADAT2	Adenosine deaminase, tRNA-specific 2, TAD2 homolog (S. cerevisiae)	ChEST 601g16	Gga.12278	Y	
ADC	Arginine decarboxylase	ChEST 685l14	Gga.27603	Y	

ADCK1	AarF domain containing kinase 1	ChEST 743d15	Gga.9041		
ADCY2	Adenylate cyclase 2 (brain)	ChEST 374f13	Gga.32479	Y	
ADCY5	Transcribed locus, strongly similar to NP_001026461.1 ubiquitin specific peptidase 1 [Gallus gallus]	ChEST 630b3	Gga.44179	Y	
ADCY7	Adenylate cyclase 7	ChEST 665o15	Gga.6878	Y	
ADCY8	Adenylate cyclase 8 (brain)	ChEST 666a7	Gga.31179	Y	
ADH1B	Alcohol dehydrogenase 1B (class I), beta polypeptide	ChEST 638o10	Gga.3415	Y	Y
ADH5	Alcohol dehydrogenase 5 (class III), chi polypeptide	ChEST 651d8	Gga.5361	Y	Y

ADI1	Acireductone dioxygenase 1	ChEST 514l10	Gga.22198	Y	Y
ADK	Adenosine kinase	ChEST 638i18	Gga.5967	Y	
ADPGK	ADP-dependent glucokinase	ChEST 651m6	Gga.19334	Y	
ADPRHL1	ADP-ribosylhydrolase like 1	ChEST 644h7	Gga.9319	Y	Y
ADPRHL2	ADP-ribosylhydrolase like 2	ChEST 634m20	Gga.42574	Y	
ADRBK2	Adrenergic, beta, receptor kinase 2	ChEST 654k9	Gga.6318	Y	
ADSL	Adenylosuccinate lyase	ChEST 712o16	Gga.2721	Y	

ADSS	Adenylosuccinate synthase	ChEST 715f7	Gga.7656	Y	
ADSSL1	Adenylosuccinate synthase like 1	ChEST 629f5	Gga.44496	Y	Y
AER61	Glycosyltransferase	ChEST 696g8	Gga.2971	Y	
AFF4	Transcribed locus, moderately similar to XP_001231351.1 PREDICTED: similar to RNase H, putative [Gallus gallus]	ChEST 719m14	Gga.23922	Y	
AFG3L2	AFG3 ATPase family gene 3-like 2 (yeast)	ChEST 564e21	Gga.38348		
AFMID	Arylformamidase	ChEST 640c18	Gga.42222	Y	Y
AGA	Aspartylglucosaminidase	ChEST 799f9	Gga.17609		

AGL	Amylo-1, 6-glucosidase, 4-alpha-glucanotransferase (glycogen debranching enzyme, glycogen storage disease type III)	ChEST 645o1	Gga.10021	Y	
AGMAT	Agmatine ureohydrolase (agmatinase)	ChEST 656h24	Gga.39549	Y	
AGPAT2	1-acylglycerol-3-phosphate O-acyltransferase 2 (lysophosphatidic acid acyltransferase, beta)	ChEST 600f11	Gga.35462	Y	
AGPAT3	1-acylglycerol-3-phosphate O-acyltransferase 3	ChEST 600f5	Gga.42852		
AGPAT4	1-acylglycerol-3-phosphate O-acyltransferase 4 (lysophosphatidic acid acyltransferase, delta)	ChEST 677n6	Gga.35608		
AGPAT5	1-acylglycerol-3-phosphate O-acyltransferase 5 (lysophosphatidic acid acyltransferase, epsilon)	ChEST 711b8	Gga.12634	Y	Y
AGPAT6	1-acylglycerol-3-phosphate O-acyltransferase 6 (lysophosphatidic acid acyltransferase, zeta)	ChEST 650b14	Gga.20363	Y	Y

AGPS	Alkylglycerone phosphate synthase	ChEST 634d16	Gga.33967	Y	
AGT	Angiotensinogen (serpin peptidase inhibitor, clade A, member 8)	ChEST 564c12	Gga.12910	Y	
AGXT2	Alanine-glyoxylate aminotransferase 2	ChEST 710p13	Gga.42318	Y	
AGXT2L1	Alanine-glyoxylate aminotransferase 2-like 1	ChEST 597e21	Gga.23549	Y	
AHCY	S-adenosylhomocysteine hydrolase	ChEST 652m4	Gga.13433	Y	
AHCYL1	S-adenosylhomocysteine hydrolase-like 1	ChEST 563h16	Gga.5821	Y	
AHSA1	AHA1, activator of heat shock 90kDa protein ATPase homolog 1 (yeast)	ChEST 669p5	Gga.4814	Y	Y

AHSA2	AHA1, activator of heat shock 90kDa protein ATPase homolog 2 (yeast)	ChEST 629h7	Gga.9158	Y	Y
AK2	Adenylate kinase 2	ChEST 609d1	Gga.5472	Y	
AKAP1	A kinase (PRKA) anchor protein 1	ChEST 619m23	Gga.21848	Y	
AKAP10	A kinase (PRKA) anchor protein 10	ChEST 676h13	Gga.42445	Y	
AKAP12	A kinase (PRKA) anchor protein (gravin) 12	ChEST 667k11	Gga.12197	Y	
AKAP13	A kinase (PRKA) anchor protein 13	ChEST 662d11	Gga.15979	Y	
AKAP7	A kinase (PRKA) anchor protein 7	ChEST 700o20	Gga.10836		

AKAP8L	A kinase (PRKA) anchor protein 8-like	ChEST 629f6	Gga.22850	Y	Y
AKAP9	A kinase (PRKA) anchor protein (yotiao) 9	ChEST 620i9	Gga.9661	Y	
AKR1A1	Aldo-keto reductase family 1, member A1 (aldehyde reductase)	ChEST 684m5	Gga.22636		
AKR1B10	Aldo-keto reductase family 1, member B10 (aldose reductase)	ChEST 606d11	Gga.4869	Y	
AKR1B10	Aldo-keto reductase family 1, member B10 (aldose reductase)	ChEST 601n9	Gga.4170		
AKR1D1	Aldo-keto reductase family 1, member D1 (delta 4-3-ketosteroid-5-beta-reductase)	ChEST 709f4	Gga.4536	Y	
AKR7A2	Aldo-keto reductase family 7, member A2 (aflatoxin aldehyde reductase)	ChEST 514d24	Gga.6415	Y	Y

ALAS1	Aminolevulinate, delta-, synthase 1	ChEST 629h6	Gga.1399		
ALDH1A1	Aldehyde dehydrogenase 1 family, member A1	ChEST 592d15	Gga.4119	Y	
ALDH1A2	Aldehyde dehydrogenase 1 family, member A2	ChEST 564a12	Gga.2996	Y	Y
ALDH1A3	Aldehyde dehydrogenase 1 family, member A3	ChEST 387g12	Gga.3807	Y	Y
ALDH1L2	Aldehyde dehydrogenase 1 family, member L2	ChEST 648l21	Gga.11316	Y	
ALDH3A2	Aldehyde dehydrogenase 3 family, member A2	ChEST 654h4	Gga.4559	Y	
ALDH4A1	Aldehyde dehydrogenase 4 family, member A1	ChEST 637n9	Gga.43069	Y	Y

ALDH5A1	Aldehyde dehydrogenase 5 family, member A1 (succinate-semialdehyde dehydrogenase)	ChEST 635e12	Gga.29220	Y	Y
ALDH6A1	Aldehyde dehydrogenase 6 family, member A1	ChEST 732o7	Gga.28479	Y	Y
ALDH7A1	Aldehyde dehydrogenase 7 family, member A1	ChEST 622g8	Gga.11454	Y	
ALDH9A1	Aldehyde dehydrogenase 9 family, member A1	ChEST 622a13	Gga.19278	Y	Y
ALDOB	Aldolase B, fructose-bisphosphate	ChEST 606l5	Gga.4760		
ALG1	Asparagine-linked glycosylation 1 homolog (S. cerevisiae, beta-1,4-mannosyltransferase)	ChEST 586d12	Gga.26166	Y	
ALG11	Asparagine-linked glycosylation 11 homolog (S. cerevisiae, alpha-1,2-mannosyltransferase)	ChEST 656e13	Gga.12321	Y	

ALG12	Asparagine-linked glycosylation 12 homolog (S. cerevisiae, alpha-1,6-mannosyltransferase)	ChEST 621g6	Gga.39520	Y	
ALG3	Asparagine-linked glycosylation 3 homolog (S. cerevisiae, alpha-1,3-mannosyltransferase)	ChEST 666e24	Gga.30585	Y	Y
ALG5	Asparagine-linked glycosylation 5 homolog (S. cerevisiae, dolichyl-phosphate beta-glucosyltransferase)	ChEST 613b17	Gga.23608	Y	
ALG6	Asparagine-linked glycosylation 6 homolog (S. cerevisiae, alpha-1,3-glucosyltransferase)	ChEST 628n24	Gga.5118	Y	Y
ALG9	Asparagine-linked glycosylation 9 homolog (S. cerevisiae, alpha- 1,2-mannosyltransferase)	ChEST 586n23	Gga.17067	Y	
ALK	Anaplastic lymphoma kinase (Ki-1)	ChEST 664g24	Gga.40382	Y	
ALOX5AP	Arachidonate 5-lipoxygenase-activating protein	ChEST 644g14	Gga.1758	Y	

ALPK3	Alpha-kinase 3	ChEST 666e12	Gga.3675	Y	
ALPL	Alkaline phosphatase, liver/bone/kidney	ChEST 586p13	Gga.760	Y	
AMD1	Adenosylmethionine decarboxylase 1	ChEST 666p2	Gga.8864	Y	
AMDHD1	Amidohydrolase domain containing 1	ChEST 649p13	Gga.27414	Y	
AMDHD2	Amidohydrolase domain containing 2	ChEST 657p16	Gga.10211	Y	
AMDHD2	Amidohydrolase domain containing 2	ChEST 666p23	Gga.16366		
AMY2A	Amylase, alpha 2A; pancreatic	ChEST 612m17	Gga.16588	Y	

ANAPC1	Anaphase promoting complEX subunit 1	ChEST 621h1	Gga.30386	Y	
ANAPC10	Anaphase promoting complEX subunit 10	ChEST 600p18	Gga.21808		
ANAPC13	Anaphase promoting complEX subunit 13	ChEST 706c10	Gga.19115	Y	
ANAPC2	Anaphase promoting complEX subunit 2	ChEST 700b3	Gga.19872		
ANAPC4	Anaphase promoting complEX subunit 4	ChEST 728b11	Gga.17940	Y	Y
ANAPC5	Anaphase promoting complEX subunit 5	ChEST 724p5	Gga.22370	Y	
ANAPC7	Anaphase promoting complEX subunit 7	ChEST 689p20	Gga.21416	Y	

ANKRD43	Transcribed locus, weakly similar to NP_065161.2 solute carrier family 44, member 2 [Homo sapiens]	ChEST 624c13	Gga.27306	Y	
AOC3	Amine oxidase, copper containing 3 (vascular adhesion protein 1)	ChEST 593j24	Gga.4560	Y	
AOF2	Amine oxidase (flavin containing) domain 2	ChEST 655h8	Gga.8775	Y	
APAF1	Apoptotic peptidase activating factor 1	ChEST 630a17	Gga.34905	Y	
ARFGAP1	ADP-ribosylation factor GTPase activating protein 1	ChEST 619a9	Gga.5835	Y	
ARFGAP2	ADP-ribosylation factor GTPase activating protein 2	ChEST 662n17	Gga.8475	Y	
ARFGAP3	ADP-ribosylation factor GTPase activating protein 3	ChEST 677f21	Gga.7324		

ARFIP2	Transcribed locus, weakly similar to NP_828874.2 fatty acid desaturase domain family, member 6 [Mus musculus]	ChEST 586j23	Gga.43139	Y	
ARHGAP1	Rho GTPase activating protein 1	ChEST 655n5	Gga.11859	Y	
ARHGAP10	Rho GTPase activating protein 10	ChEST 642l12	Gga.10013	Y	
ARHGAP11A	Rho GTPase activating protein 11A	ChEST 625o15	Gga.4825	Y	
ARHGAP12	Rho GTPase activating protein 12	ChEST 630g11	Gga.30983	Y	Y
ARHGAP17	Rho GTPase activating protein 17	ChEST 606n7	Gga.5448	Y	Y
ARHGAP18	Rho GTPase activating protein 18	ChEST 699d3	Gga.29207	Y	Y

ARHGAP19	Rho GTPase activating protein 19	ChEST 641d4	Gga.7609	Y	Y
ARHGAP21	Rho GTPase activating protein 21	ChEST 629i16	Gga.30236	Y	Y
ARHGAP22	Rho GTPase activating protein 22	ChEST 578d20	Gga.25086	Y	
ARHGAP24	Rho GTPase activating protein 24	ChEST 571m19	Gga.39289		
ARHGAP25	Rho GTPase activating protein 25	ChEST 610n24	Gga.22173		
ARHGAP26	Rho GTPase activating protein 26	ChEST 705b14	Gga.2901	Y	
ARHGAP28	Rho GTPase activating protein 28	ChEST 670a23	Gga.10285	Y	Y

ARHGAP29	Rho GTPase activating protein 29	ChEST 617n6	Gga.22027	Y	
ARHGAP5	Rho GTPase activating protein 5	ChEST 514g23	Gga.29858	Y	Y
ARHGAP8	Rho GTPase activating protein 8	ChEST 597e7	Gga.31650		
ARSA	Arylsulfatase A	ChEST 578o2	Gga.29059	Y	
ARSB	Similar to Arylsulfatase B precursor (ASB) (N-acetylgalactosamine-4-sulfatase) (G4S)	ChEST 731i6	Gga.23916	Y	Y
ARSE	Arylsulfatase E (chondrodysplasia punctata 1)	ChEST 732f18	Gga.27728	Y	
ARSH	Arylsulfatase family, member H	ChEST 578g13	Gga.3339		

ARSJ	Arylsulfatase family, member J	ChEST 711o17	Gga.19935	Y	Y
AS3MT	Arsenic (+3 oxidation state) methyltransferase	ChEST 602c12	Gga.12497	Y	
ASAH1	N-acylsphingosine amidohydrolase (acid ceramidase) 1	ChEST 649j23	Gga.17521	Y	Y
ASAH3	N-acylsphingosine amidohydrolase (alkaline ceramidase) 3	ChEST 604a20	Gga.8214	Y	
ASL	Argininosuccinate lyase	ChEST 743a15	Gga.17624		
ASMTL	Acetylserotonin O-methyltransferase-like	ChEST 578j19	Gga.14188	Y	
ASNS	Asparagine synthetase	ChEST 649j21	Gga.22299	Y	Y

ASNSD1	Asparagine synthetase domain containing 1	ChEST 514g22	Gga.22269	Y	
ASPH	Aspartate beta-hydroxylase	ChEST 374m9	Gga.8808	Y	
ASPHD2	Aspartate beta-hydroxylase domain containing 2	ChEST 604f13	Gga.23357	Y	
ASRGL1	Asparaginase like 1	ChEST 646n20	Gga.36750	Y	
ATAD1	ATPase family, AAA domain containing 1	ChEST 593l22	Gga.16792	Y	
ATAD2	ATPase family, AAA domain containing 2	ChEST 718j5	Gga.30569		
ATAD2B	ATPase family, AAA domain containing 2B	ChEST 604i20	Gga.11705	Y	

ATAD3A	ATPase family, AAA domain containing 3A	ChEST 514n7	Gga.19048	Y	Y
ATE1	Arginyltransferase 1	ChEST 619i7	Gga.8240	Y	
ATHL1	ATH1, acid trehalase-like 1 (yeast)	ChEST 698j11	Gga.37280	Y	
ATIC	5-aminoimidazole-4-carboxamide ribonucleotide formyltransferase/IMP cyclohydrolase	ChEST 387g5	Gga.4556	Y	
ATP10D	ATPase, class V, type 10D	ChEST 735j24	Gga.9127	Y	Y
ATP11A	ATPase, class VI, type 11A	ChEST 666p11	Gga.31112	Y	
ATP11B	ATPase, class VI, type 11B	ChEST 634n6	Gga.12881	Y	

ATP11C	ATPase, class VI, type 11C	ChEST 727e16	Gga.15933	Y	
ATP13A1	ATPase type 13A1	ChEST 617b23	Gga.3161	Y	
ATP1A1	ATPase, Na+/K+ transporting, alpha 1 polypeptide	ChEST 608a20	Gga.2155	Y	
ATP1B1	ATPase, Na+/K+ transporting, beta 1 polypeptide	ChEST 608e11	Gga.3301	Y	
ATP1B3	ATPase, Na+/K+ transporting, beta 3 polypeptide	ChEST 387p9	Gga.42003	Y	
ATP1B4	ATPase, (Na+)/K+ transporting, beta 4 polypeptide	ChEST 563o15	Gga.40342	Y	
ATP2A2	ATPase, Ca++ transporting, cardiac muscle, slow twitch 2	ChEST 600l7	Gga.42608	Y	Y

ATP2B1	ATPase, Ca++ transporting, plasma membrane 1	ChEST 709l8	Gga.30308	Y	Y
ATP2B2	ATPase, Ca++ transporting, plasma membrane 2	ChEST 610d11	Gga.23886	Y	Y
ATP2B4	ATPase, Ca++ transporting, plasma membrane 4	ChEST 620n18	Gga.43222		
ATP2C1	ATPase, Ca++ transporting, type 2C, member 1	ChEST 603l18	Gga.34891	Y	
ATP5A1W	ATP synthase subunit alpha	ChEST 650o8	Gga.39680	Y	Y
ATP6AP1	ATPase, H+ transporting, lysosomal accessory protein 1	ChEST 609e23	Gga.23512		
ATP6V0A1	ATPase, H+ transporting, lysosomal V0 subunit a1	ChEST 606d21	Gga.43082	Y	Y

ATP6V0A2	ATPase, H+ transporting, lysosomal V0 subunit A2	ChEST 637e16	Gga.259	Y	
ATP6V0B	ATPase, H+ transporting, lysosomal 21kDa, V0 subunit b	ChEST 374n14	Gga.4592	Y	
ATP6V0D1	ATPase, H+ transporting, lysosomal 38kDa, V0 subunit d1	ChEST 675g24	Gga.7507	Y	
ATP6V0E1	ATPase, H+ transporting, lysosomal 9kDa, V0 subunit e1	ChEST 612l24	Gga.9402	Y	
ATP6V0E2	ATPase, H+ transporting V0 subunit e2	ChEST 578l19	Gga.12238	Y	Y
ATP6V1A	ATPase, H+ transporting, lysosomal 70kDa, V1 subunit A	ChEST 635o14	Gga.1712	Y	Y
ATP6V1B2	ATPase, H+ transporting, lysosomal 56/58kDa, V1 subunit B2	ChEST 639h17	Gga.3876	Y	Y

ATP6V1C1	ATPase, H+ transporting, lysosomal 42kDa, V1 subunit C1	ChEST 600o10	Gga.11586	Y	
ATP6V1C2	ATPase, H+ transporting, lysosomal 42kDa, V1 subunit C2	ChEST 593j16	Gga.45481	Y	Y
ATP6V1D	ATPase, H+ transporting, lysosomal 34kDa, V1 subunit D	ChEST 661a15	Gga.8201	Y	
ATP6V1E1	ATPase, H+ transporting, lysosomal 31kDa, V1 subunit E1	ChEST 612d22	Gga.4861	Y	
ATP6V1G1	ATPase, H+ transporting, lysosomal 13kDa, V1 subunit G1	ChEST 610j1	Gga.4824	Y	
ATP7A	ATPase, Cu++ transporting, alpha polypeptide (Menkes syndrome)	ChEST 387a10	Gga.24950	Y	
ATP7B	ATPase, Cu++ transporting, beta polypeptide	ChEST 724c13	Gga.31312		

ATP8A1	ATPase, aminophospholipid transporter (APLT), class I, type 8A, member 1	ChEST 606e12	Gga.21972	Y	
ATP8A2	ATPase, aminophospholipid transporter-like, class I, type 8A, member 2	ChEST 617n21	Gga.30877	Y	Y
ATP9B	ATPase, class II, type 9B	ChEST 663l11	Gga.22236		
ATXN2	Transcribed locus, strongly similar to NP_036845.2 protein kinase C, beta [Rattus norvegicus]	ChEST 735b17	Gga.41859		
AVEN	Apoptosis, caspase activation inhibitor	ChEST 713b20	Gga.9342		
AYTL1	Acyltransferase like 1	ChEST 643a3	Gga.21018	Y	
AYTL2	Acyltransferase like 2	ChEST 563d19	Gga.16935	Y	

B3GALNT2	Beta-1,3-N-acetylgalactosaminyltransferase 2	ChEST 571a4	Gga.39722	Y	
B3GALT2	UDP-Gal:betaGlcNAc beta 1,3-galactosyltransferase, polypeptide 2	ChEST 564o24	Gga.10401	Y	Y
B3GALTL	Beta 1,3-galactosyltransferase-like	ChEST 600d21	Gga.12863	Y	
B3GAT2	Beta-1,3-glucuronyltransferase 2 (glucuronosyltransferase S)	ChEST 709i1	Gga.43613	Y	Y
B3GNT2	UDP-GlcNAc:betaGal beta-1,3-N-acetylglucosaminyltransferase 2	ChEST 564l13	Gga.2149		
B3GNT4	UDP-GlcNAc:betaGal beta-1,3-N-acetylglucosaminyltransferase 4	ChEST 600j15	Gga.30773	Y	
B3GNT5	UDP-GlcNAc:betaGal beta-1,3-N-acetylglucosaminyltransferase 5	ChEST 655l12	Gga.28486	Y	

B3GNT7	UDP-GlcNAc:betaGal beta-1,3-N-acetylglucosaminyltransferase 7	ChEST 634p4	Gga.9567	Y	Y
B3GNTL1	UDP-GlcNAc:betaGal beta-1,3-N-acetylglucosaminyltransferase-like 1	ChEST 656m3	Gga.39996	Y	
B4GALNT4	Beta-1,4-N-acetyl-galactosaminyl transferase 4	ChEST 659l6	Gga.29606	Y	
B4GALT1	UDP-Gal:betaGlcNAc beta 1,4-galactosyltransferase, polypeptide 1	ChEST 713i9	Gga.2840	Y	
B4GALT4	UDP-Gal:betaGlcNAc beta 1,4-galactosyltransferase, polypeptide 4	ChEST 586p12	Gga.13390	Y	
B4GALT5	UDP-Gal:betaGlcNAc beta 1,4-galactosyltransferase, polypeptide 5	ChEST 716h5	Gga.27509	Y	
B4GALT6	UDP-Gal:betaGlcNAc beta 1,4-galactosyltransferase, polypeptide 6	ChEST 737h1	Gga.14725	Y	

B4GALT7	Xylosylprotein beta 1,4-galactosyltransferase, polypeptide 7 (galactosyltransferase I)	ChEST 697j3	Gga.33756	Y	
BAAT	Bile acid Coenzyme A: amino acid N-acyltransferase (glycine N-choloyltransferase)	ChEST 736g21	Gga.35265	Y	
BAP1	BRCA1 associated protein-1 (ubiquitin carboxy-terminal hydrolase)	ChEST 593j13	Gga.5871	Y	
BCAT1	Branched chain aminotransferase 1, cytosolic	ChEST 651b1	Gga.6577	Y	Y
BCDO2	Beta-carotene dioxygenase 2	ChEST 725h4	Gga.28262	Y	
BCKDHB	Branched chain keto acid dehydrogenase E1, beta polypeptide (maple syrup urine disease)	ChEST 514k9	Gga.3355	Y	
BCL2	Transcribed locus, moderately similar to NP_113770.2 amine oxidase, copper containing 3 [Rattus norvegicus]	ChEST 637j11	Gga.43255	Y	

BDH1	3-hydroxybutyrate dehydrogenase, type 1	ChEST 514n19	Gga.5444	Y	Y
BDH2	3-hydroxybutyrate dehydrogenase, type 2	ChEST 593f18	Gga.2925	Y	
BHMT	Betaine-homocysteine methyltransferase	ChEST 651e14	Gga.23580	Y	
BLK	B lymphoid tyrosine kinase	ChEST 731p15	Gga.44582	Y	
BLMH	Bleomycin hydrolase	ChEST 643g3	Gga.4171	Y	Y
BLVRA	Biliverdin reductase A	ChEST 644m1	Gga.6017	Y	
BMP2K	BMP2 inducible kinase	ChEST 601f15	Gga.42728		

BMPR2	Bone morphogenetic protein receptor, type II (serine/threonine kinase)	ChEST 622g11	Gga.105	Y	
BPGM	2,3-bisphosphoglycerate mutase	ChEST 563c24	Gga.22606		Y
BPHL	Biphenyl hydrolase-like (serine hydrolase; breast epithelial mucin-associated antigen)	ChEST 601k11	Gga.12024		
BRIP1	BRCA1 interacting protein C-terminal helicase 1	ChEST 705c14	Gga.17801	Y	
BTD	Biotinidase	ChEST 735j21	Gga.11847	Y	
BTK	Bruton agammaglobulinemia tyrosine kinase	ChEST 710o11	Gga.1305	Y	
C1GALT1	Core 1 synthase, glycoprotein-N-acetylgalactosamine 3-beta-galactosyltransferase, 1	ChEST 601g6	Gga.1326	Y	Y

C5orf22	Transcribed locus, strongly similar to NP_071545.2 BRbeta B-regulatory subunit of protein phosphatase 2A [Rattus norvegicus]	ChEST 664c8	Gga.29292		
CA10	Carbonic anhydrase X	ChEST 652b10	Gga.42943	Y	
CA10	Transcribed locus, strongly similar to XP_420314.2 PREDICTED: similar to p21-activated kinase 3 [Gallus gallus]	ChEST 709b12	Gga.42959		
CA10	Transcribed locus, moderately similar to NP_056372.1 chromodomain helicase DNA binding protein 5 [Homo sapiens]	ChEST 374p4	Gga.42960		
CA2	Carbonic anhydrase II	ChEST 674c9	Gga.3986	Y	
CA4	Carbonic anhydrase IV	ChEST 520m7	Gga.6513	Y	
CA6	Carbonic anhydrase VI	ChEST 593n22	Gga.11019	Y	

CA8	Carbonic anhydrase VIII	ChEST 645f2	Gga.2072		
CACYBP	Transcribed locus, moderately similar to NP_001995.1 farnesyl diphosphate synthase [Homo sapiens]	ChEST 611a19	Gga.11604	Y	
CALM3	Calmodulin 3 (phosphorylase kinase, delta)	ChEST 644l17	Gga.31374	Y	
CAMK1G	Calcium/calmodulin-dependent protein kinase IG	ChEST 610j10	Gga.43525	Y	
CAMK2A	Calcium/calmodulin-dependent protein kinase (CaM kinase) II alpha	ChEST 675m3	Gga.9623	Y	
CAMK2G	Calcium/calmodulin-dependent protein kinase (CaM kinase) II gamma	ChEST 658l24	Gga.3324	Y	
CAMKK2	Calcium/calmodulin-dependent protein kinase kinase 2, beta	ChEST 658p15	Gga.30132	Y	Y

CANT1	Calcium activated nucleotidase 1	ChEST 622d10	Gga.22494	Y	
CAP2	CAP, adenylate cyclase-associated protein, 2 (yeast)	ChEST 578m20	Gga.5445	Y	
CARD10	Caspase recruitment domain family, member 10	ChEST 592i3	Gga.25961	Y	Y
CARD11	Caspase recruitment domain family, member 11	ChEST 563p5	Gga.42269	Y	
CARD9	Caspase recruitment domain family, member 9	ChEST 624d15	Gga.17565	Y	
CARKL	Carbohydrate kinase-like	ChEST 689f14	Gga.10883	Y	
CARS	Cysteinyl-tRNA synthetase	ChEST 571l16	Gga.22936	Y	Y

CASK	Calcium/calmodulin-dependent serine protein kinase (MAGUK family)	ChEST 609b21	Gga.7689	Y	Y
CASP2	Caspase 2, apoptosis-related cysteine peptidase (neural precursor cell EXpressed, developmentally down-regulated 2)	ChEST 601i8	Gga.504	Y	
CASP3	Caspase 3, apoptosis-related cysteine peptidase	ChEST 669o21	Gga.4346		
CASP6	Caspase 6, apoptosis-related cysteine peptidase	ChEST 650h18	Gga.2960	Y	
CASP8	Caspase 8, apoptosis-related cysteine peptidase	ChEST 620e3	Gga.2451	Y	
CASP9	Caspase 9, apoptosis-related cysteine peptidase	ChEST 654j22	Gga.4116	Y	
CBR1	Carbonyl reductase 1	ChEST 620f16	Gga.2896	Y	

CBR4	Carbonyl reductase 4	ChEST 729m3	Gga.9673	Y	Y
CCBL1	Cysteine conjugate-beta lyase; cytoplasmic (glutamine transaminase K, kyneurenine aminotransferase)	ChEST 586c2	Gga.34909	Y	
CCBL2	Cysteine conjugate-beta lyase 2	ChEST 644n11	Gga.29199	Y	Y
CCDC81	Transcribed locus, strongly similar to NP_032865.1 phosphatidylinositol 3-kinase, catalytic, alpha polypeptide [Mus musculus]	ChEST 635o21	Gga.44414	Y	Y
CCDC94	Casein kinase II beta subunit	ChEST 652o14	Gga.34034	Y	
CDA	Cytidine deaminase	ChEST 387g16	Gga.6600	Y	
CDA	Cytidine deaminase	ChEST 387g16	Gga.6600		

CDADC1	Cytidine and dCMP deaminase domain containing 1	ChEST 564m11	Gga.10629		
CDAN1	Transcribed locus, strongly similar to XP_683534.1 PREDICTED: similar to Phosphoinositide-3-kinase, regulatory subunit, polypeptide 3 (p55, gamma) isoform 1 [Danio rerio]	ChEST 601j3	Gga.44012		
CDC2L5	Cell division cycle 2-like 5 (cholinesterase-related cell division controller)	ChEST 630k22	Gga.20556	Y	Y
CDC42BPB	CDC42 binding protein kinase beta (DMPK-like)	ChEST 605i9	Gga.16259	Y	Y
CDC42EP3	CDC42 effector protein (Rho GTPase binding) 3	ChEST 641n18	Gga.11749	Y	
CDC42EP4	CDC42 effector protein (Rho GTPase binding) 4	ChEST 604n8	Gga.2643	Y	
CDD	Cytidine deaminase	ChEST 714p8	Gga.458	Y	

CDH13	Transcribed locus, strongly similar to NP_001022583.1 MAP Kinase family member (mpk-1) [Caenorhabditis elegans]	ChEST 678j6	Gga.24275	Y	
CDK10	Cyclin-dependent kinase 10	ChEST 661l1	Gga.12919	Y	
CDK8	Cyclin-dependent kinase 8	ChEST 734o11	Gga.18915	Y	
CDK9	Cyclin-dependent kinase 9	ChEST 651c6	Gga.2483		
CDKL1	Cyclin-dependent kinase-like 1 (CDC2-related kinase)	ChEST 661l13	Gga.31322	Y	
CDKL2	Cyclin-dependent kinase-like 2 (CDC2-related kinase)	ChEST 605h16	Gga.12315	Y	
CDKN2B	Cyclin-dependent kinase inhibitor 2B (p15, inhibits CDK4)	ChEST 620a9	Gga.5007	Y	Y

CDKN2B	Cyclin-dependent kinase inhibitor 2B (p15, inhibits CDK4)	ChEST 620a9	Gga.5007		
CDKN3	Cyclin-dependent kinase inhibitor 3 (CDK2-associated dual specificity phosphatase)	ChEST 634e13	Gga.6343	Y	
CDO1	Cysteine dioxygenase, type I	ChEST 622g17	Gga.6921		
CDS2	CDP-diacylglycerol synthase (phosphatidate cytidylyltransferase) 2	ChEST 624b15	Gga.7937	Y	
CEL	Carboxyl ester lipase (bile salt-stimulated lipase)	ChEST 615a20	Gga.6379		
CEPT1	Choline/ethanolamine phosphotransferase 1	ChEST 639e6	Gga.2534	Y	
CERK	Ceramide kinase	ChEST 667j3	Gga.16530	Y	

CES1	Similar to thioesterase B	ChEST 730m19	Gga.42199	Y	
CH25H	Cholesterol 25-hydroxylase	ChEST 739l4	Gga.10928		
CHD1	Chromodomain helicase DNA binding protein 1	ChEST 604e6	Gga.4301	Y	
CHD1L	Chromodomain helicase DNA binding protein 1-like	ChEST 657k13	Gga.13260	Y	
CHD2	Chromodomain helicase DNA binding protein 2	ChEST 563a19	Gga.21117	Y	Y
CHD7	Chromodomain helicase DNA binding protein 7	ChEST 605j13	Gga.20865		
CHDH	Choline dehydrogenase	ChEST 663l5	Gga.24748	Y	Y

CHID1	Chitinase domain containing 1	ChEST 586k3	Gga.10703	Y	
CHPT1	Choline phosphotransferase 1	ChEST 578o9	Gga.15627	Y	
CHRNA5	Transcribed locus, strongly similar to XP_414081.2 PREDICTED: similar to Dipeptidase 2 [Gallus gallus]	ChEST 604c19	Gga.29570	Y	
CHST10	Carbohydrate sulfotransferase 10	ChEST 628e12	Gga.19459	Y	Y
CHST15	N-acetylgalactosamine 4-sulfate 6-O-sulfotransferase	ChEST 593i9	Gga.3676	Y	Y
CHST6	Carbohydrate (N-acetylglucosamine 6-O) sulfotransferase 6	ChEST 644a20	Gga.20134	Y	
CHST9	Carbohydrate (N-acetylgalactosamine 4-0) sulfotransferase 9	ChEST 374m2	Gga.30426	Y	

CILP	Cartilage intermediate layer protein, nucleotide pyrophosphohydrolase	ChEST 658p2	Gga.15814	Y	Y
CINP	Cyclin-dependent kinase 2-interacting protein	ChEST 628k12	Gga.12284	Y	Y
CIT	Citron (rho-interacting, serine/threonine kinase 21)	ChEST 720b6	Gga.27615	Y	
CKB	Creatine kinase, brain	ChEST 606f22	Gga.2722	Y	Y
CKS1B	CDC28 protein kinase regulatory subunit 1B	ChEST 683j9	Gga.7132	Y	Y
CKS2	CDC28 protein kinase regulatory subunit 2	ChEST 691i3	Gga.1958		
CLASP1	Transcribed locus, strongly similar to NP_033010.1 protein tyrosine phosphatase, receptor type, M [Mus musculus]	ChEST 671l24	Gga.43658	Y	

CLCN5	Chloride channel 5 (nephrolithiasis 2, X-linked, Dent disease)	ChEST 637m7	Gga.39350	Y	
CLYBL	Citrate lyase beta like	ChEST 670l6	Gga.20464	Y	Y
CMAS	Cytidine monophosphate N-acetylneuraminic acid synthetase	ChEST 593m6	Gga.19097	Y	
CMPK	Cytidylate kinase	ChEST 629c5	Gga.19231	Y	
CNDP1	Carnosine dipeptidase 1 (metallopeptidase M20 family)	ChEST 681p9	Gga.10874	Y	
CNDP2	CNDP dipeptidase 2 (metallopeptidase M20 family)	ChEST 609n18	Gga.22514		
CNKSR2	Connector enhancer of kinase suppressor of Ras 2	ChEST 713i22	Gga.23535	Y	Y

CNP	2',3'-cyclic nucleotide 3' phosphodiesterase	ChEST 593c6	Gga.4098	Y	
COASY	Coenzyme A synthase	ChEST 696m12	Gga.45779		
Collagen, type II	Transcribed locus, weakly similar to XP_001476675.1 PREDICTED: similar to Collagen-like tail subunit (single strand of homotrimer) of asymmetric acetylcholinesterase isoform 3 [Mus	ChEST 606c3	Gga.47292	Y	Y
COMT	Catechol-O-methyltransferase	ChEST 677d14	Gga.7199	Y	Y
COMTD1	Catechol-O-methyltransferase domain containing 1	ChEST 602e16	Gga.28865	Y	
COQ2	Coenzyme Q2 homolog, prenyltransferase (yeast)	ChEST 645f5	Gga.2317	Y	Y
COQ3	Coenzyme Q3 homolog, methyltransferase (S. cerevisiae)	ChEST 637g19	Gga.11388	Y	

COQ5	Coenzyme Q5 homolog, methyltransferase (S. cerevisiae)	ChEST 374n20	Gga.9227	Y	
COQ6	Coenzyme Q6 homolog, monooxygenase (S. cerevisiae)	ChEST 601h14	Gga.27254	Y	
COX10	COX10 homolog, cytochrome c oxidase assembly protein, heme A: farnesyltransferase (yeast)	ChEST 601e7	Gga.20356	Y	
COX11	COX11 homolog, cytochrome c oxidase assembly protein (yeast)	ChEST 642o18	Gga.23618	Y	
COX15	COX15 homolog, cytochrome c oxidase assembly protein (yeast)	ChEST 631e5	Gga.39991	Y	Y
COX19	COX19 cytochrome c oxidase assembly homolog (S. cerevisiae)	ChEST 603m21	Gga.9157		
COX4I1	Cytochrome c oxidase subunit IV isoform 1	ChEST 564d6	Gga.4877		

COX6A1	Cytochrome c oxidase subunit VIa polypeptide 1	ChEST 593c10	Gga.39155	Y	
COX6C	Cytochrome c oxidase subunit VIc	ChEST 603f10	Gga.5304	Y	
COX7A2L	Cytochrome c oxidase subunit VIIa polypeptide 2 like	ChEST 661m1	Gga.5926	Y	
COX7C	Cytochrome c oxidase subunit VIIc	ChEST 669l14	Gga.6171	Y	
CP	Ceruloplasmin (ferroxidase)	ChEST 640n12	Gga.36425	Y	
CPA1	Carboxypeptidase A1 (pancreatic)	ChEST 615i21	Gga.9498	Y	
CPA2	Carboxypeptidase A2 (pancreatic)	ChEST 615a1	Gga.5946		

CPA5	Carboxypeptidase A5	ChEST 615l14	Gga.45925	Y	
CPA6	Carboxypeptidase A6	ChEST 658p5	Gga.13803	Y	Y
CPB1	Carboxypeptidase B1 (tissue)	ChEST 615a11	Gga.6059		
CPD	Carboxypeptidase D	ChEST 374d19	Gga.7594	Y	
CPE	Carboxypeptidase E	ChEST 605i12	Gga.5494	Y	
CPM	Carboxypeptidase M	ChEST 533b18	Gga.11927	Y	Y
CPO	Carboxypeptidase O	ChEST 639i19	Gga.29836	Y	

CPOX	Coproporphyrinogen oxidase	ChEST 630e3	Gga.39565	Y	
CPT2	Carnitine palmitoyltransferase II	ChEST 624o2	Gga.22581	Y	Y
CRIP2	Transcribed locus, strongly similar to XP_413889.2 PREDICTED: similar to Carbohydrate (chondroitin) synthase 1 [Gallus gallus]	ChEST 655b7	Gga.42634		
CRLS1	Cardiolipin synthase 1	ChEST 387o7	Gga.11346	Y	
CROT	Carnitine O-octanoyltransferase	ChEST 630e17	Gga.9995	Y	Y
CRY1	Cryptochrome 1 (photolyase-like)	ChEST 604m9	Gga.4107	Y	
CRY2	Cryptochrome 2 (photolyase-like)	ChEST 610m6	Gga.20033		

CRYZ	Crystallin, zeta (quinone reductase)	ChEST 597c18	Gga.3621		
CRYZL1	Crystallin, zeta (quinone reductase)-like 1	ChEST 646p1	Gga.1923		
CRYZL1	Transcribed locus, moderately similar to NP_766578.1 pantothenate kinase 4 [Mus musculus]	ChEST 696h20	Gga.1924		
CSGALNACT1	Chondroitin sulfate N-acetylgalactosaminyltransferase 1	ChEST 647n6	Gga.44592		
CSGALNACT2	Chondroitin sulfate N-acetylgalactosaminyltransferase 2	ChEST 709e9	Gga.30577	Y	
CSK	C-src tyrosine kinase	ChEST 696i10	Gga.2691	Y	
CSNK1A1	Casein kinase 1, alpha 1	ChEST 619o2	Gga.3287	Y	

CSNK1D	Casein kinase 1, delta	ChEST 605c4	Gga.34543	Y	
CSNK1E	Casein kinase 1, epsilon	ChEST 630j10	Gga.2087	Y	Y
CSNK1G1	Casein kinase 1, gamma 1	ChEST 643e22	Gga.15908		
CSNK2A1	Casein kinase 2, alpha 1 polypeptide	ChEST 714f10	Gga.3243	Y	
CTBS	Chitobiase, di-N-acetyl-	ChEST 670m17	Gga.11457		
CTH	Cystathionase (cystathionine gamma-lyase)	ChEST 677j12	Gga.2872	Y	
CTTN	Transcribed locus, strongly similar to XP_415856.2 PREDICTED: similar to A kinase (PRKA) anchor protein 10 [Gallus gallus]	ChEST 643h4	Gga.39286	Y	

CYB5R	NADH-cytochrome b5 reductase	ChEST 676o13	Gga.4373	Y	
CYB5R4	Cytochrome b5 reductase 4	ChEST 701e23	Gga.22221	Y	
CYP1A4	Transcribed locus, moderately similar to NP_004712.1 mitogen-activated protein kinase kinase kinase 13 [Homo sapiens]	ChEST 604f7	Gga.43125	Y	
DAGLB	Diacylglycerol lipase, beta	ChEST 641p18	Gga.5540	Y	Y
DAK	Dihydroxyacetone kinase 2 homolog (S. cerevisiae)	ChEST 619d9	Gga.42653	Y	
DARS	Aspartyl-tRNA synthetase	ChEST 627b22	Gga.7544	Y	
DBT	Dihydrolipoamide branched chain transacylase E2	ChEST 653f3	Gga.187	Y	

DCI	Dodecenoyl-Coenzyme A delta isomerase (3,2 trans-enoyl-Coenzyme A isomerase)	ChEST 520i7	Gga.11641	Y	Y
DCK	Deoxycytidine kinase	ChEST 604c14	Gga.16670		
DCTD	DCMP deaminase	ChEST 627e2	Gga.22181	Y	
DCXR	Dicarbonyl/L-xylulose reductase	ChEST 514i13	Gga.2276		
DDAH1	Dimethylarginine dimethylaminohydrolase 1	ChEST 709o20	Gga.13255	Y	Y
DDC	Dopa decarboxylase (aromatic L-amino acid decarboxylase)	ChEST 634g2	Gga.31021		
DDO	D-aspartate oxidase	ChEST 724c17	Gga.34964	Y	

DDOST	Dolichyl-diphosphooligosaccharide-protein glycosyltransferase	ChEST 520g23	Gga.13565	Y	Y
DDT	D-dopachrome tautomerase	ChEST 644m4	Gga.7005	Y	Y
DDX11	DEAD/H (Asp-Glu-Ala-Asp/His) box polypeptide 11 (CHL1-like helicase homolog, S. cerevisiae)	ChEST 746p11	Gga.30003	Y	
DEGS1	Degenerative spermatocyte homolog 1, lipid desaturase (Drosophila)	ChEST 520c11	Gga.8162	Y	
DGAT2	Diacylglycerol O-acyltransferase homolog 2 (mouse)	ChEST 676n10	Gga.37973	Y	
DGKB	Diacylglycerol kinase, beta 90kDa	ChEST 746d19	Gga.40533	Y	Y
DGKD	Diacylglycerol kinase, delta 130kDa	ChEST 663c18	Gga.42141		

DGKQ	Diacylglycerol kinase, theta 110kDa	ChEST 732i22	Gga.25332		
DGKQ	Diacylglycerol kinase, theta 110kDa	ChEST 732i22	Gga.25332		
DGKZ	Diacylglycerol kinase, zeta 104kDa	ChEST 628g1	Gga.5646	Y	
DHCR24	24-dehydrocholesterol reductase	ChEST 701n17	Gga.7345		
DHCR7	7-dehydrocholesterol reductase	ChEST 631o10	Gga.14081	Y	Y
DHDDS	Dehydrodolichyl diphosphate synthase	ChEST 603a9	Gga.31084	Y	
DHDDS	Dehydrodolichyl diphosphate synthase	ChEST 603a9	Gga.31084		

DHFR	Dihydrofolate reductase	ChEST 667c19	Gga.2883	Y	Y
DHODH	Dihydroorotate dehydrogenase	ChEST 691i11	Gga.4705		
DHRS2	Dehydrogenase/reductase (SDR family) member 2	ChEST 600e22	Gga.35194	Y	
DHRS3	Dehydrogenase/reductase (SDR family) member 3	ChEST 374c6	Gga.7235	Y	
DHRS7	Dehydrogenase/reductase (SDR family) member 7	ChEST 606k20	Gga.5401		
DHRS7B	Dehydrogenase/reductase (SDR family) member 7B	ChEST 638j2	Gga.7346	Y	
DHRS7C	Dehydrogenase/reductase (SDR family) member 7C	ChEST 514d23	Gga.11882	Y	Y

DHRSX	Dehydrogenase/reductase (SDR family) X-linked	ChEST 578o22	Gga.12183	Y	
DHTKD1	Dehydrogenase E1 and transketolase domain containing 1	ChEST 611j9	Gga.31755		
DIMT1L	DIM1 dimethyladenosine transferase 1-like (S. cerevisiae)	ChEST 571f23	Gga.17588	Y	
DIO1	Deiodinase, iodothyronine, type I	ChEST 732b23	Gga.553	Y	Y
DIO2	Deiodinase, iodothyronine, type II	ChEST 709g8	Gga.1819		
DLD	Dihydrolipoamide dehydrogenase	ChEST 978i4	Gga.4909	Y	
DNA2	DNA replication helicase 2 homolog (yeast)	ChEST 665p15	Gga.42409	Y	

DNASE1L3	Deoxyribonuclease I-like 3	ChEST 635i22	Gga.11830	Y	
DNASE2B	Deoxyribonuclease II beta	ChEST 622f24	Gga.16501	Y	
DNPEP	Aspartyl aminopeptidase	ChEST 514g11	Gga.8509	Y	Y
DNTTIP1	Deoxynucleotidyltransferase, terminal, interacting protein 1	ChEST 667k1	Gga.12491	Y	
DNTTIP2	Deoxynucleotidyltransferase, terminal, interacting protein 2	ChEST 699g9	Gga.43580	Y	
DOHH	Deoxyhypusine hydroxylase/monooxygenase	ChEST 709m15	Gga.7510	Y	
DOLPP1	Dolichyl pyrophosphate phosphatase 1	ChEST 619c10	Gga.5388		

DPAGT1	Dolichyl-phosphate (UDP-N-acetylglucosamine) N-acetylglucosaminephosphotransferase 1 (GlcNAc-1-P transferase)	ChEST 586b3	Gga.9701		
DPEP2	Dipeptidase 2	ChEST 715h8	Gga.40845	Y	
DPM1	Dolichyl-phosphate mannosyltransferase polypeptide 1, catalytic subunit	ChEST 630j17	Gga.1475	Y	
DPP10	Dipeptidyl-peptidase 10	ChEST 665p2	Gga.22042	Y	
DPP4	Dipeptidyl-peptidase 4 (CD26, adenosine deaminase complEXing protein 2)	ChEST 662m18	Gga.8886	Y	
DPP7	Dipeptidyl-peptidase 7	ChEST 627k5	Gga.5356		
DPP9	Dipeptidyl-peptidase 9	ChEST 670e4	Gga.2079	Y	

DPY19L1	Transcribed locus, moderately similar to XP_001506269.1 PREDICTED: similar to Procollagen-lysine, 2-oxoglutarate 5-dioxygenase 2 [Ornithorhynchus anatinus]	ChEST 824j6	Gga.31105	Y	
DPYD	Dihydropyrimidine dehydrogenase	ChEST 641k8	Gga.20542		
DPYS	Dihydropyrimidinase	ChEST 586a7	Gga.9647	Y	Y
DPYSL2	Dihydropyrimidinase-like 2	ChEST 662e22	Gga.3569		
DPYSL3	Dihydropyrimidinase-like 3	ChEST 533a21	Gga.9493	Y	Y
DPYSL4	Dihydropyrimidinase-like 4	ChEST 663g2	Gga.1428		
DSE	Dermatan sulfate epimerase	ChEST 703l22	Gga.11086	Y	

DTD1	D-tyrosyl-tRNA deacylase 1 homolog (S. cerevisiae)	ChEST 674l13	Gga.9939		
DUS1L	Dihydrouridine synthase 1-like (S. cerevisiae)	ChEST 593o21	Gga.5501	Y	
DUS2L	Dihydrouridine synthase 2-like, SMM1 homolog (S. cerevisiae)	ChEST 654f1	Gga.6313	Y	
DUS3L	Dihydrouridine synthase 3-like (S. cerevisiae)	ChEST 734f19	Gga.15947	Y	
DUS4L	Dihydrouridine synthase 4-like (S. cerevisiae)	ChEST 700a10	Gga.12453	Y	
DUSP1	Dual specificity phosphatase 1	ChEST 620c22	Gga.4120	Y	
DUSP10	Dual specificity phosphatase 10	ChEST 609b22	Gga.16879		

DUSP13	Dual specificity phosphatase 13	ChEST 736b20	Gga.10263	Y	
DUSP14	Dual specificity phosphatase 14	ChEST 660l19	Gga.6368	Y	
DUSP16	Dual specificity phosphatase 16	ChEST 641f19	Gga.30699	Y	
DUSP19	Dual specificity phosphatase 19	ChEST 629g15	Gga.23324	Y	
DUSP26	Dual specificity phosphatase 26 (putative)	ChEST 664b4	Gga.12480	Y	
DUSP27	Dual specificity phosphatase 27 (putative)	ChEST 647b2	Gga.10994		
DUSP28	Dual specificity phosphatase 28	ChEST 738k21	Gga.26396	Y	

DUSP5	Dual specificity phosphatase 5	ChEST 630i17	Gga.28858	Y	Y
DUSP6	Dual specificity phosphatase 6	ChEST 650l18	Gga.8445	Y	
DUT	DUTP pyrophosphatase	ChEST 638g2	Gga.5602	Y	
DYRK2	Dual-specificity tyrosine-(Y)-phosphorylation regulated kinase 2	ChEST 602c4	Gga.14403	Y	
DYRK3	Dual-specificity tyrosine-(Y)-phosphorylation regulated kinase 3	ChEST 668b12	Gga.7340	Y	Y
ECHDC1	Enoyl Coenzyme A hydratase domain containing 1	ChEST 630k14	Gga.10129	Y	
ECHDC2	Enoyl Coenzyme A hydratase domain containing 2	ChEST 514b14	Gga.9702		

ECHDC3	Enoyl Coenzyme A hydratase domain containing 3	ChEST 620k2	Gga.6284	Y	
EDEM1	ER degradation enhancer, mannosidase alpha-like 1	ChEST 601k6	Gga.22434	Y	
EDEM2	ER degradation enhancer, mannosidase alpha-like 2	ChEST 592m19	Gga.22328	Y	
EDEM3	ER degradation enhancer, mannosidase alpha-like 3	ChEST 586o22	Gga.3480	Y	
EEPD1	Endonuclease/EXonuclease/phosphatase family domain containing 1	ChEST 606a19	Gga.33557	Y	
EHHADH	Enoyl-Coenzyme A, hydratase/3-hydroxyacyl Coenzyme A dehydrogenase	ChEST 669l4	Gga.7542	Y	
EHMT1	Euchromatic histone-lysine N-methyltransferase 1	ChEST 611h21	Gga.1248	Y	

EIF2AK1	Eukaryotic translation initiation factor 2-alpha kinase 1	ChEST 631f10	Gga.3983	Y	Y
EIF2AK2	Eukaryotic translation initiation factor 2-alpha kinase 2	ChEST 639k1	Gga.9292	Y	
EIF2AK3	Eukaryotic translation initiation factor 2-alpha kinase 3	ChEST 625i15	Gga.33867	Y	
ELA1	Elastase 1, pancreatic	ChEST 615d8	Gga.6129		
ELA2A	Elastase 2A	ChEST 615a12	Gga.34337		
EME1	Essential meiotic endonuclease 1 homolog 1 (S. pombe)	ChEST 604b19	Gga.15843	Y	
ENDOG	Endonuclease G	ChEST 697j22	Gga.6307	Y	

ENDOGL1	Endonuclease G-like 1	ChEST 600l20	Gga.21356	Y	
ENO2	Enolase 2 (gamma, neuronal)	ChEST 387b20	Gga.4132	Y	
ENOPH1	Enolase-phosphatase 1	ChEST 387d15	Gga.5415	Y	
ENOX2	Ecto-NOX disulfide-thiol EXchanger 2	ChEST 721m22	Gga.7033	Y	
ENPEP	Glutamyl aminopeptidase (aminopeptidase A)	ChEST 617b6	Gga.31294	Y	
ENPP1	Ectonucleotide pyrophosphatase/phosphodiesterase 1	ChEST 597c8	Gga.29447		
ENPP2	Ectonucleotide pyrophosphatase/phosphodiesterase 2 (autotaxin)	ChEST 520a5	Gga.11463	Y	Y

ENPP4	Ectonucleotide pyrophosphatase/phosphodiesterase 4 (putative function)	ChEST 593l23	Gga.21309	Y	
ENPP6	Ectonucleotide pyrophosphatase/phosphodiesterase 6	ChEST 592m4	Gga.8153	Y	
ENTPD1	Transcribed locus, strongly similar to NP_001012925.1 ectonucleoside triphosphate diphosphohydrolase 1 [Gallus gallus]	ChEST 732f5	Gga.1884	Y	Y
ENTPD1	Ectonucleoside triphosphate diphosphohydrolase 1	ChEST 638h18	Gga.40345	Y	
ENTPD2	Ectonucleoside triphosphate diphosphohydrolase 2	ChEST 639f14	Gga.44860	Y	
ENTPD4	Ectonucleoside triphosphate diphosphohydrolase 4	ChEST 621e16	Gga.22086	Y	
ENTPD5	Ectonucleoside triphosphate diphosphohydrolase 5	ChEST 593p9	Gga.9865	Y	

ENTPD6	Ectonucleoside triphosphate diphosphohydrolase 6 (putative function)	ChEST 514b2	Gga.22218	Y	
ENTPD8	Ectonucleoside triphosphate diphosphohydrolase 8	ChEST 600p8	Gga.144	Y	
EPHX1	Epoxide hydrolase 1, microsomal (xenobiotic)	ChEST 638f15	Gga.39997	Y	
EPHX2	Epoxide hydrolase 2, cytoplasmic	ChEST 601i5	Gga.39534		
EPRS	Glutamyl-prolyl-tRNA synthetase	ChEST 641k22	Gga.8167	Y	
ERNI	Transcribed locus, moderately similar to XP_870563.2 PREDICTED: similar to hEXokinase II [Bos taurus]	ChEST 648j20	Gga.46942	Y	
ESF1	Transcribed locus, strongly similar to NP_003626.1 N-deacetylase/N-sulfotransferase (heparan glucosaminyl) 2 [Homo sapiens]	ChEST 710j15	Gga.43771		

ETFDH	Electron-transferring-flavoprotein dehydrogenase	ChEST 563g14	Gga.22907	Y	
EXDL2	EXonuclease 3'-5' domain-like 2	ChEST 705c5	Gga.34613	Y	
FA2H	Fatty acid 2-hydroxylase	ChEST 701g6	Gga.8879	Y	
FAAH	Fatty acid amide hydrolase	ChEST 592d18	Gga.7143	Y	
FADS1	Fatty acid desaturase 1	ChEST 610l11	Gga.13371		
FADS2	Fatty acid desaturase 2	ChEST 578d23	Gga.1384		
FAHD1	Fumarylacetoacetate hydrolase domain containing 1	ChEST 635o13	Gga.5301	Y	

FAHD2A	Transcribed locus, strongly similar to NP_058028.2 cytidine 5'-triphosphate synthase [Mus musculus]	ChEST 656j23	Gga.39198	Y	
FAM5B	Transcribed locus, moderately similar to NP_956960.2 ilvB (bacterial acetolactate synthase)-like [Danio rerio]	ChEST 627l2	Gga.30623	Y	
FARSB	Phenylalanyl-tRNA synthetase, beta subunit	ChEST 705j18	Gga.6269		
FASN	Fatty acid synthase	ChEST 606i20	Gga.33829	Y	Y
FASTKD1	FAST kinase domains 1	ChEST 605n14	Gga.22679	Y	
FASTKD5	FAST kinase domains 5	ChEST 668m20	Gga.9846		
FBP1	Fructose-1,6-bisphosphatase 1	ChEST 641a20	Gga.5139	Y	Y

FBXO18	F-box protein, helicase, 18	ChEST 592i22	Gga.29201	Y	
FBXO30	Transcribed locus, strongly similar to NP_058735.1 protein phosphatase 2a, catalytic subunit, alpha isoform [Rattus norvegicus]	ChEST 736m4	Gga.44524	Y	
FDFT1	Farnesyl-diphosphate farnesyltransferase 1	ChEST 586h8	Gga.8817	Y	
FDPS	Farnesyl diphosphate synthase (farnesyl pyrophosphate synthetase, dimethylallyltranstransferase, geranyltranstransferase)	ChEST 563c17	Gga.42725		
FDX1	Ferredoxin 1	ChEST 593i22	Gga.891		
FDXR	Ferredoxin reductase	ChEST 720a7	Gga.23299	Y	
FECH	Ferrochelatase (protoporphyria)	ChEST 645a20	Gga.166	Y	Y

FGD5	Transcribed locus, moderately similar to XP_001515174.1 PREDICTED: similar to Methionine-tRNA synthetase, partial [Ornithorhynchus anatinus]	ChEST 586f23	Gga.12996	Y	Y
FGGY	FGGY carbohydrate kinase domain containing	ChEST 627o5	Gga.11829	Y	
FH	Fumarate hydratase	ChEST 514m22	Gga.16465	Y	
Finished cDNA,	Carboxylesterase 1 (monocyte/macrophage serine esterase 1)	ChEST 732p13	Gga.41904	Y	Y
FLT4	Fms-related tyrosine kinase 4	ChEST 612n21	Gga.439	Y	
FMO4	Transcribed locus, weakly similar to XP_001233942.1 PREDICTED: similar to flavin-containing monooxygenase 3 [Gallus gallus]	ChEST 723b23	Gga.11989	Y	Y
FMO6	Flavin containing monooxygenase 6	ChEST 608o5	Gga.4564	Y	

FNTA	Farnesyltransferase, CAAX box, alpha	ChEST 592l10	Gga.16685	Y	
FOXRED1	FAD-dependent oxidoreductase domain containing 1	ChEST 603i1	Gga.18113		
FRAS1	Fraser syndrome 1	ChEST 643l15	Gga.14594	Y	Y
FRK	Transcribed locus, moderately similar to NP_077211.3 solute carrier family 38, member 10 [Mus musculus]	ChEST 641c8	Gga.27337	Y	
FRK	Fyn-related kinase	ChEST 604d18	Gga.27336	Y	
FSTL1	Transcribed locus, strongly similar to NP_852079.2 phosphoinositide-3-kinase, class 3 [Mus musculus]	ChEST 621j7	Gga.43213		
FTCD	Formiminotransferase cyclodeaminase	ChEST 725l4	Gga.2513	Y	

FUCA1	Fucosidase, alpha-L- 1, tissue	ChEST 729h3	Gga.16198	Y	Y
FUCA2	Fucosidase, alpha-L- 2, plasma	ChEST 617i1	Gga.39580	Y	
FUCA2	Fucosidase, alpha-L- 2, plasma	ChEST 617i1	Gga.39580		
FUK	Fucokinase	ChEST 644d1	Gga.31152		
FUT10	Fucosyltransferase 10 (alpha (1,3) fucosyltransferase)	ChEST 619k8	Gga.5114	Y	
FUT11	Fucosyltransferase 11 (alpha (1,3) fucosyltransferase)	ChEST 648m19	Gga.4652		
FUT8	Fucosyltransferase 8 (alpha (1,6) fucosyltransferase)	ChEST 597c16	Gga.21415	Y	

FUT8	Fucosyltransferase 8 (alpha (1,6) fucosyltransferase)	ChEST 597c16	Gga.21415		
FUT9	Fucosyltransferase 9 (alpha (1,3) fucosyltransferase)	ChEST 620g7	Gga.29666		
G3BP1	GTPase activating protein (SH3 domain) binding protein 1	ChEST 717k8	Gga.4912	Y	
G3BP2	GTPase activating protein (SH3 domain) binding protein 2	ChEST 675j12	Gga.10005	Y	
G6PC2	Glucose-6-phosphatase, catalytic, 2	ChEST 650i19	Gga.10996	Y	Y
GAD1	Glutamate decarboxylase 1 (brain, 67kDa)	ChEST 747o14	Gga.441	Y	
GAK	Cyclin G associated kinase	ChEST 674h11	Gga.24354		

GAL3ST1	Galactose-3-O-sulfotransferase 1	ChEST 578h3	Gga.17318	Y	Y
GALC	Galactosylceramidase	ChEST 695e8	Gga.40695		
GALE	UDP-galactose-4-epimerase	ChEST 604b23	Gga.9722	Y	
GALNS	Galactosamine (N-acetyl)-6-sulfate sulfatase (Morquio syndrome, mucopolysaccharidosis type IVA)	ChEST 597d7	Gga.7601		
GALNT1	UDP-N-acetyl-alpha-D-galactosamine:polypeptide N-acetylgalactosaminyltransferase 1 (GalNAc-T1)	ChEST 617o24	Gga.21315	Y	Y
GALNT11	UDP-N-acetyl-alpha-D-galactosamine:polypeptide N-acetylgalactosaminyltransferase 11 (GalNAc-T11)	ChEST 620g5	Gga.7832	Y	Y
GALNT12	UDP-N-acetyl-alpha-D-galactosamine:polypeptide N-acetylgalactosaminyltransferase 12 (GalNAc-T12)	ChEST 715h18	Gga.10147		

GALNT13	UDP-N-acetyl-alpha-D-galactosamine:polypeptide N-acetylgalactosaminyltransferase 13 (GalNAc-T13)	ChEST 698c6	Gga.11047	Y	
GALNT14	UDP-N-acetyl-alpha-D-galactosamine:polypeptide N-acetylgalactosaminyltransferase 14 (GalNAc-T14)	ChEST 586n14	Gga.25956	Y	Y
GALNT2	UDP-N-acetyl-alpha-D-galactosamine:polypeptide N-acetylgalactosaminyltransferase 2 (GalNAc-T2)	ChEST 387c24	Gga.24283	Y	Y
GALNT3	UDP-N-acetyl-alpha-D-galactosamine:polypeptide N-acetylgalactosaminyltransferase 3 (GalNAc-T3)	ChEST 605f11	Gga.14608		
GALNT7	UDP-N-acetyl-alpha-D-galactosamine:polypeptide N-acetylgalactosaminyltransferase 7 (GalNAc-T7)	ChEST 563d17	Gga.36734	Y	
GALNT9	UDP-N-acetyl-alpha-D-galactosamine:polypeptide N-acetylgalactosaminyltransferase 9 (GalNAc-T9)	ChEST 668p18	Gga.20452		
GALNTL4	UDP-N-acetyl-alpha-D-galactosamine:polypeptide N-acetylgalactosaminyltransferase-like 4	ChEST 698h18	Gga.11756	Y	

GALT	Galactose-1-phosphate uridylyltransferase	ChEST 745l18	Gga.44032	Y	
GANC	Glucosidase, alpha; neutral C	ChEST 624a6	Gga.16880	Y	
GAPDH	Glyceraldehyde-3-phosphate dehydrogenase	ChEST 520o24	Gga.6383	Y	Y
GAPVD1	GTPase activating protein and VPS9 domains 1	ChEST 374j15	Gga.35476	Y	
GARNL1	GTPase activating Rap/RanGAP domain-like 1	ChEST 520e20	Gga.14842		
GARNL3	GTPase activating Rap/RanGAP domain-like 3	ChEST 643m22	Gga.15450	Y	
GARS	Glycyl-tRNA synthetase	ChEST 387i4	Gga.9376		

GART	Phosphoribosylglycinamide formyltransferase, phosphoribosylglycinamide synthetase, phosphoribosylaminoimidazole synthetase	ChEST 662f18	Gga.4704	Y	Y
GATC	Glutamyl-tRNA(Gln) amidotransferase, subunit C homolog (bacterial)	ChEST 387f15	Gga.5155		
GATM	Glycine amidinotransferase (L-arginine:glycine amidinotransferase)	ChEST 713d16	Gga.1167	Y	
GBA	Glucosidase, beta; acid (includes glucosylceramidase)	ChEST 660p1	Gga.39834	Y	
GBE1	Glucan (1,4-alpha-), branching enzyme 1 (glycogen branching enzyme, Andersen disease, glycogen storage disease type IV)	ChEST 514e1	Gga.16784	Y	
GCAT	Glycine C-acetyltransferase (2-amino-3-ketobutyrate coenzyme A ligase)	ChEST 675c3	Gga.38247	Y	
GCH1	GTP cyclohydrolase 1 (dopa-responsive dystonia)	ChEST 629n24	Gga.674	Y	

GCLC	Glutamate-cysteine ligase, catalytic subunit	ChEST 374k2	Gga.10006	Y	
GCLM	Glutamate-cysteine ligase, modifier subunit	ChEST 597b16	Gga.2525		
GCNT1	Glucosaminyl (N-acetyl) transferase 1, core 2 (beta-1,6-N-acetylglucosaminyltransferase)	ChEST 716j24	Gga.24378	Y	
GCSH	Glycine cleavage system protein H (aminomethyl carrier)	ChEST 606m3	Gga.3298		
GDE1	Glycerophosphodiester phosphodiesterase 1	ChEST 675f2	Gga.8868	Y	
GDPD1	Glycerophosphodiester phosphodiesterase domain containing 1	ChEST 637d21	Gga.36571	Y	
GDPD2	Glycerophosphodiester phosphodiesterase domain containing 2	ChEST 622i18	Gga.30017		

GDPD4	Glycerophosphodiester phosphodiesterase domain containing 4	ChEST 669p19	Gga.12107	Y	
GFOD1	Glucose-fructose oxidoreductase domain containing 1	ChEST 708m20	Gga.31287		
GFOD2	Glucose-fructose oxidoreductase domain containing 2	ChEST 605p19	Gga.19149	Y	Y
GFPT1	Glutamine-fructose-6-phosphate transaminase 1	ChEST 622h1	Gga.13536	Y	Y
GGPS1	Geranylgeranyl diphosphate synthase 1	ChEST 685f21	Gga.22036	Y	
GGT1	Gamma-glutamyltransferase 1	ChEST 593o14	Gga.9387	Y	Y
GGT5	Gamma-glutamyltransferase 5	ChEST 713l12	Gga.36578	Y	Y

GGT7	Gamma-glutamyltransferase 7	ChEST 671c20	Gga.7588	Y	
GIMAP1	GTPase, IMAP family member 1	ChEST 603i12	Gga.31831		
GIMAP5	GTPase, IMAP family member 5	ChEST 622b6	Gga.30006	Y	
GK	Glycerol kinase	ChEST 604n13	Gga.11825		
GKAP1	G kinase anchoring protein 1	ChEST 608f1	Gga.5218		
GLA	Galactosidase, alpha	ChEST 719i6	Gga.43141		
GLDC	Glycine dehydrogenase (decarboxylating)	ChEST 645n8	Gga.3393	Y	

GLRX2	Glutaredoxin 2	ChEST 374b12	Gga.39542	Y	
GLRX3	Glutaredoxin 3	ChEST 657o2	Gga.1498	Y	
GLRX5	Glutaredoxin 5	ChEST 592k13	Gga.21412	Y	
GLS	Glutaminase	ChEST 593p11	Gga.43009	Y	
GLT25D2	Glycosyltransferase 25 domain containing 2	ChEST 678l24	Gga.3249	Y	
GLT8D1	Glycosyltransferase 8 domain containing 1	ChEST 514d16	Gga.5247	Y	
GLT8D2	Glycosyltransferase 8 domain containing 2	ChEST 387n8	Gga.11247	Y	

GLT8D4	Glycosyltransferase 8 domain containing 4	ChEST 658m17	Gga.11809	Y	
GLUL	Glutamate-ammonia ligase (glutamine synthetase)	ChEST 564d19	Gga.2464	Y	Y
GLYCTK	Glycerate kinase	ChEST 617h21	Gga.35373		
GMDS	GDP-mannose 4,6-dehydratase	ChEST 666l11	Gga.5698	Y	
GMPPB	Transcribed locus, moderately similar to NP_038813.2 inositol hEXaphosphate kinase 1 [Mus musculus]	ChEST 722d9	Gga.9810	Y	Y
GMPPB	GDP-mannose pyrophosphorylase B	ChEST 625b24	Gga.9810	Y	
GMPS	Guanine monphosphate synthetase	ChEST 586b5	Gga.7671	Y	Y

GNE	Glucosamine (UDP-N-acetyl)-2-epimerase/N-acetylmannosamine kinase	ChEST 635h15	Gga.9568	Y	
GNMT	Glycine N-methyltransferase	ChEST 648c20	Gga.6256	Y	
GNPAT	Glyceronephosphate O-acyltransferase	ChEST 739e7	Gga.26211		
GNPDA1	Glucosamine-6-phosphate deaminase 1	ChEST 611d20	Gga.8913	Y	
GNPNAT1	Glucosamine-phosphate N-acetyltransferase 1	ChEST 637e4	Gga.10583		
GNS	Glucosamine (N-acetyl)-6-sulfatase (Sanfilippo disease IIID)	ChEST 650o3	Gga.24203	Y	
GOT1	Glutamic-oxaloacetic transaminase 1, soluble (aspartate aminotransferase 1)	ChEST 608o19	Gga.730	Y	Y

GPD1	Glycerol-3-phosphate dehydrogenase 1 (soluble)	ChEST 601b23	Gga.34629	Y	Y
GPI	Glucose phosphate isomerase	ChEST 606c19	Gga.4883	Y	
GPT2	Glutamic pyruvate transaminase (alanine aminotransferase) 2	ChEST 734g14	Gga.42761	Y	Y
GPX4	Glutathione peroxidase 4 (phospholipid hydroperoxidase)	ChEST 520i11	Gga.107		
GPX7	Glutathione peroxidase 7	ChEST 613g3	Gga.37228		
GRB2	Transcribed locus, strongly similar to NP_990201.1 Janus kinase 1 (a protein tyrosine kinase) [Gallus gallus]	ChEST 612n14	Gga.41900		
GRHPR	Glyoxylate reductase/hydroxypyruvate reductase	ChEST 689c20	Gga.35242	Y	

GRK5	G protein-coupled receptor kinase 5	ChEST 715h2	Gga.24472	Y	Y
GSK3B	Glycogen synthase kinase 3 beta	ChEST 601a8	Gga.7650	Y	
GSK3B	Glycogen synthase kinase 3 beta	ChEST 601a8	Gga.7650		
GSPT1	G1 to S phase transition 1	ChEST 612l22	Gga.39395	Y	
GSR	Glutathione reductase	ChEST 578b5	Gga.34900	Y	
GSS	Glutathione synthetase	ChEST 592n6	Gga.29690	Y	
GSTA	Glutathione S-transferase class-alpha	ChEST 656p20	Gga.39241	Y	

GSTA1	Glutathione S-transferase A1	ChEST 604d13	Gga.2533		
GSTCD	Glutathione S-transferase, C-terminal domain containing	ChEST 638f5	Gga.31591	Y	Y
GSTCD	Transcribed locus, weakly similar to NP_766433.1 a disintegrin-like and metalloprotease (reprolysin type) with thrombospondin type 1 motif, 4 [Mus musculus]	ChEST 678d1	Gga.31596	Y	Y
GSTK1	Glutathione S-transferase kappa 1	ChEST 514l23	Gga.5689	Y	Y
GSTO1	Glutathione S-transferase omega 1	ChEST 638k17	Gga.10968	Y	
GSTT1	Glutathione S-transferase theta 1	ChEST 611d16	Gga.2437	Y	Y
GSTT1	Similar to Glutathione S-transferase theta 1	ChEST 608e10	Gga.6265	Y	Y

GSTZ1	Glutathione transferase zeta 1 (maleylacetoacetate isomerase)	ChEST 639i22	Gga.1022	Y	Y
GTF2E1	Transcribed locus, moderately similar to XP_534796.2 PREDICTED: similar to Serine/threonine-protein kinase receptor R3 precursor (SKR3) (Activin receptor-like kinase 1) (ALK-1) (TGF-B	ChEST 648c6	Gga.29949	Y	
GUCA1B	Guanylate cyclase activator 1B (retina)	ChEST 708e20	Gga.624	Y	
GUCY1B3	Guanylate cyclase 1, soluble, beta 3	ChEST 714p9	Gga.36939	Y	
GUCY2C	Guanylate cyclase 2C (heat stable enterotoxin receptor)	ChEST 597g3	Gga.29752	Y	
GUK1	Guanylate kinase 1	ChEST 638g8	Gga.11124		
H6PD	HEXose-6-phosphate dehydrogenase (glucose 1-dehydrogenase)	ChEST 619b10	Gga.44427	Y	

HAAO	3-hydroxyanthranilate 3,4-dioxygenase	ChEST 734k10	Gga.9514	Y	Y
HACE1	HECT domain and ankyrin repeat containing, E3 ubiquitin protein ligase 1	ChEST 740l18	Gga.22685	Y	
HACL1	2-hydroxyacyl-CoA lyase 1	ChEST 654k4	Gga.24400	Y	
HADHA	Hydroxyacyl-Coenzyme A dehydrogenase/3-ketoacyl-Coenzyme A thiolase/enoyl-Coenzyme A hydratase (trifunctional protein), alpha subunit	ChEST 653b10	Gga.8616	Y	Y
HADHB	Hydroxyacyl-Coenzyme A dehydrogenase/3-ketoacyl-Coenzyme A thiolase/enoyl-Coenzyme A hydratase (trifunctional protein), beta subunit	ChEST 514l7	Gga.6352	Y	Y
HAGH	Hydroxyacylglutathione hydrolase	ChEST 514d2	Gga.8337	Y	Y
HAGHL	Hydroxyacylglutathione hydrolase-like	ChEST 732p20	Gga.6006		

HAL	Histidine ammonia-lyase	ChEST 737l4	Gga.8448	Y	Y
HARS	Histidyl-tRNA synthetase	ChEST 720c22	Gga.22462	Y	
HAT1	Histone acetyltransferase 1	ChEST 696k9	Gga.2476	Y	
HCK	Hemopoietic cell kinase	ChEST 730p21	Gga.43170	Y	
HDAC1	Histone deacetylase 1	ChEST 605h10	Gga.14557	Y	Y
HDAC10	Histone deacetylase 10	ChEST 605f1	Gga.27678		
HDAC11	Histone deacetylase 11	ChEST 722n2	Gga.11485		

HDAC2	Histone deacetylase 2	ChEST 578a20	Gga.2951		
HDAC3	Histone deacetylase 3	ChEST 586j20	Gga.42569	Y	Y
HDAC4	Histone deacetylase 4	ChEST 604d2	Gga.3689	Y	
HDAC7A	Histone deacetylase 7A	ChEST 601m24	Gga.22946		
HDAC7A	Histone deacetylase 7A	ChEST 601m24	Gga.22946		
HDAC8	Histone deacetylase 8	ChEST 649p21	Gga.11652	Y	
HDHD1A	Haloacid dehalogenase-like hydrolase domain containing 1A	ChEST 635e11	Gga.11419	Y	

HDHD2	Haloacid dehalogenase-like hydrolase domain containing 2	ChEST 656i13	Gga.12875	Y	
HDHD3	Haloacid dehalogenase-like hydrolase domain containing 3	ChEST 592m1	Gga.5250	Y	
HECW2	HECT, C2 and WW domain containing E3 ubiquitin protein ligase 2	ChEST 716l15	Gga.20444	Y	
HELB	Helicase (DNA) B	ChEST 667k16	Gga.33886	Y	
HELLS	Helicase, lymphoid-specific	ChEST 533o4	Gga.16540	Y	Y
HELZ	Helicase with zinc finger	ChEST 635n17	Gga.41934	Y	
HEXB	HEXosaminidase B (beta polypeptide)	ChEST 578d3	Gga.39748		

HGD	Homogentisate 1,2-dioxygenase (homogentisate oxidase)	ChEST 662c9	Gga.10831	Y	
HGF	Transcribed locus, strongly similar to XP_426495.2 PREDICTED: similar to GTPase-like protein [Gallus gallus]	ChEST 600i24	Gga.28386	Y	
HGS	Hepatocyte growth factor-regulated tyrosine kinase substrate	ChEST 660n14	Gga.7570	Y	
HGSNAT	Heparan-alpha-glucosaminide N-acetyltransferase	ChEST 619g18	Gga.26859	Y	
HIBADH	3-hydroxyisobutyrate dehydrogenase	ChEST 605d14	Gga.22571	Y	
HIBCH	3-hydroxyisobutyryl-Coenzyme A hydrolase	ChEST 533k23	Gga.9512	Y	
HIPK1	Homeodomain interacting protein kinase 1	ChEST 651b14	Gga.43481		

HIPK2	Homeodomain interacting protein kinase 2	ChEST 605c17	Gga.12888		
HIPK3	Homeodomain interacting protein kinase 3	ChEST 628h11	Gga.34207	Y	Y
HISPPD1	Histidine acid phosphatase domain containing 1	ChEST 651i15	Gga.24904	Y	
HISPPD2A	Histidine acid phosphatase domain containing 2A	ChEST 639f16	Gga.35692	Y	
HK2	HEXokinase 2	ChEST 654i21	Gga.1055	Y	
HLCS	Holocarboxylase synthetase (biotin-(proprionyl-Coenzyme A-carboxylase (ATP-hydrolysing)) ligase)	ChEST 603d9	Gga.13968	Y	
HMBS	Hydroxymethylbilane synthase	ChEST 620f2	Gga.8480	Y	

HMGCL	3-hydroxymethyl-3-methylglutaryl-Coenzyme A lyase	ChEST 533i17	Gga.2537		
HMGCLL1	3-hydroxymethyl-3-methylglutaryl-Coenzyme A lyase-like 1	ChEST 681a20	Gga.10461	Y	
HMGCR	3-hydroxy-3-methylglutaryl-Coenzyme A reductase	ChEST 578l21	Gga.2785	Y	
HMGCS1	3-hydroxy-3-methylglutaryl-Coenzyme A synthase 1 (soluble)	ChEST 514p6	Gga.42190	Y	Y
HMOX1	Heme oxygenase (decycling) 1	ChEST 677g24	Gga.2039	Y	
HMOX2	Heme oxygenase (decycling) 2	ChEST 592a11	Gga.38023	Y	
HNMT	Histamine N-methyltransferase	ChEST 629i9	Gga.10841	Y	

HPD	4-hydroxyphenylpyruvate dioxygenase	ChEST 610g23	Gga.1573		
HPDL	4-hydroxyphenylpyruvate dioxygenase-like	ChEST 695h16	Gga.24927		
HPGD	Hydroxyprostaglandin dehydrogenase 15-(NAD)	ChEST 631d24	Gga.9639	Y	Y
HPRT1	Hypoxanthine phosphoribosyltransferase 1 (Lesch-Nyhan syndrome)	ChEST 621h7	Gga.3545	Y	
HPSE	Heparanase	ChEST 625a23	Gga.950	Y	
HS2ST1	Heparan sulfate 2-O-sulfotransferase 1	ChEST 571p24	Gga.9290		
HS3ST1	Heparan sulfate (glucosamine) 3-O-sulfotransferase 1	ChEST 643d10	Gga.7526	Y	Y

HS3ST3A1	Heparan sulfate (glucosamine) 3-O-sulfotransferase 3A1	ChEST 676m4	Gga.31125	Y	Y
HS6ST1	Heparan sulfate 6-O-sulfotransferase 1	ChEST 606o9	Gga.9316	Y	Y
HS6ST2	Heparan sulfate 6-O-sulfotransferase 2	ChEST 729l8	Gga.2632	Y	Y
HSD11B1	Hydroxysteroid (11-beta) dehydrogenase 1	ChEST 640g23	Gga.35210	Y	Y
HSD17B10	Hydroxysteroid (17-beta) dehydrogenase 10	ChEST 649m3	Gga.25889	Y	
HSD17B11	Hydroxysteroid (17-beta) dehydrogenase 11	ChEST 698c13	Gga.39476	Y	
HSD17B12	Hydroxysteroid (17-beta) dehydrogenase 12	ChEST 683c6	Gga.43065	Y	

HSD17B13	Hydroxysteroid (17-beta) dehydrogenase 13	ChEST 657c10	Gga.5242		
HSD17B2	Hydroxysteroid (17-beta) dehydrogenase 2	ChEST 643c11	Gga.40121	Y	Y
HSD17B4	Hydroxysteroid (17-beta) dehydrogenase 4	ChEST 617f5	Gga.3546		
HSD17B7	Hydroxysteroid (17-beta) dehydrogenase 7	ChEST 387i1	Gga.7215	Y	
HSD3B1	Hydroxy-delta-5-steroid dehydrogenase, 3 beta- and steroid delta-isomerase 1	ChEST 704l19	Gga.596	Y	Y
HSD3B7	Hydroxy-delta-5-steroid dehydrogenase, 3 beta- and steroid delta-isomerase 7	ChEST 533g6	Gga.12018	Y	
HSDL1	Hydroxysteroid dehydrogenase like 1	ChEST 745j19	Gga.1854		

HTRA3	HtrA serine peptidase 3	ChEST 625h5	Gga.5710		
IAH1	Isoamyl acetate-hydrolyzing esterase 1 homolog (S. cerevisiae)	ChEST 634c19	Gga.12549	Y	
IARS	Isoleucyl-tRNA synthetase	ChEST 649m23	Gga.20183	Y	
ICK	Intestinal cell (MAK-like) kinase	ChEST 641p14	Gga.12148	Y	
ICMT	Isoprenylcysteine carboxyl methyltransferase	ChEST 600o18	Gga.16529	Y	
IDH1	Isocitrate dehydrogenase 1 (NADP+), soluble	ChEST 606d16	Gga.1861	Y	Y
IDH3A	Isocitrate dehydrogenase 3 (NAD+) alpha	ChEST 592m14	Gga.19046	Y	Y

IDH3B	Isocitrate dehydrogenase 3 (NAD+) beta	ChEST 592d24	Gga.5623	Y	Y
IDI1	Isopentenyl-diphosphate delta isomerase 1	ChEST 592k24	Gga.8851	Y	
IDUA	Iduronidase, alpha-L-	ChEST 592k1	Gga.19650	Y	
IFIH1	Interferon induced with helicase C domain 1	ChEST 699a13	Gga.16457		
ILK	Integrin-linked kinase	ChEST 643l19	Gga.3404	Y	
ILKAP	Integrin-linked kinase-associated serine/threonine phosphatase 2C	ChEST 645p13	Gga.12903	Y	Y
IMPA1	Inositol(myo)-1(or 4)-monophosphatase 1	ChEST 627g1	Gga.9803	Y	

IMPA2	Inositol(myo)-1(or 4)-monophosphatase 2	ChEST 612a1	Gga.42028	Y	
IMPA2	Transcribed locus, strongly similar to XP_001235322.1 PREDICTED: similar to Alanyl-tRNA synthetase domain containing 1 [Gallus gallus]	ChEST 571n15	Gga.42029		
IMPAD1	Inositol monophosphatase domain containing 1	ChEST 666h23	Gga.12302	Y	
INPP4A	Inositol polyphosphate-4-phosphatase, type I, 107kDa	ChEST 746j9	Gga.30265	Y	Y
INPP5A	Transcribed locus, strongly similar to NP_001013951.1 vacuolar ATPase subunit H [Rattus norvegicus]	ChEST 628f17	Gga.43445	Y	Y
INPP5A	Inositol polyphosphate-5-phosphatase, 40kDa	ChEST 387h17	Gga.43437		Y
INPP5B	Inositol polyphosphate-5-phosphatase, 75kDa	ChEST 605c7	Gga.12952	Y	

INPP5D	Inositol polyphosphate-5-phosphatase, 145kDa	ChEST 621b3	Gga.42297	Y	
INPP5E	Inositol polyphosphate-5-phosphatase, 72 kDa	ChEST 514j20	Gga.39364	Y	Y
INPP5F	Inositol polyphosphate-5-phosphatase F	ChEST 710n2	Gga.6172		
IPMK	Inositol polyphosphate multikinase	ChEST 600c6	Gga.39592	Y	
IQGAP1	IQ motif containing GTPase activating protein 1	ChEST 586h17	Gga.24296	Y	
IQGAP2	Similar to Ras GTPase-activating-like protein IQGAP2	ChEST 625f10	Gga.14355	Y	Y
IRAK2	Interleukin-1 receptor-associated kinase 2	ChEST 606d13	Gga.7865	Y	

IRAK4	Interleukin-1 receptor-associated kinase 4	ChEST 651l23	Gga.22347		
ISG20L1	Interferon stimulated EXonuclease gene 20kDa-like 1	ChEST 668e4	Gga.27940	Y	Y
ISG20L2	Transcribed locus, strongly similar to NP_990276.1 smooth muscle protein phosphatase type 1-binding subunit [Gallus gallus]	ChEST 672h8	Gga.1337		
ITCH	Itchy homolog E3 ubiquitin protein ligase (mouse)	ChEST 689g20	Gga.10549	Y	
ITGA6	Transcribed locus, strongly similar to XP_416566.2 PREDICTED: similar to Cdc42 GTPase-activating protein [Gallus gallus]	ChEST 714m18	Gga.44591		
ITPA	Inosine triphosphatase (nucleoside triphosphate pyrophosphatase)	ChEST 563a11	Gga.11897	Y	
ITPK1	Inositol 1,3,4-triphosphate 5/6 kinase	ChEST 597g1	Gga.18974	Y	

IVD	Isovaleryl Coenzyme A dehydrogenase	ChEST 649k24	Gga.31605	Y	
JAK2	Janus kinase 2 (a protein tyrosine kinase)	ChEST 639i6	Gga.954	Y	Y
JAKMIP2	Janus kinase and microtubule interacting protein 2	ChEST 719g10	Gga.17987	Y	Y
JAKMIP3	Janus kinase and microtubule interacting protein 3	ChEST 661h5	Gga.39898	Y	Y
KALRN	Kalirin, RhoGEF kinase	ChEST 605a5	Gga.24692	Y	
KARS	Lysyl-tRNA synthetase	ChEST 685b21	Gga.4487	Y	
KATNA1	Katanin p60 (ATPase-containing) subunit A 1	ChEST 374o5	Gga.12204	Y	

KAZALD1	Kazal-type serine peptidase inhibitor domain 1	ChEST 703k14	Gga.5651	Y	
KDR	Kinase insert domain receptor (a type III receptor tyrosine kinase)	ChEST 642o7	Gga.7339	Y	Y
KEL	Kell blood group, metallo-endopeptidase	ChEST 799k10	Gga.40397	Y	Y
KIF1A	Transcribed locus, strongly similar to XP_421130.2 PREDICTED: similar to Serine peptidase inhibitor, Kunitz type 1 [Gallus gallus]	ChEST 619m10	Gga.43390	Y	Y
KRTCAP3	Transcribed locus, weakly similar to NP_001016671.1 hydroxysteroid (17-beta) dehydrogenase 8 [Xenopus tropicalis]	ChEST 600c22	Gga.7770		
KSR1	Kinase suppressor of ras 1	ChEST 631n1	Gga.5633	Y	Y
KYNU	Kynureninase (L-kynurenine hydrolase)	ChEST 628a10	Gga.7801	Y	

LACTB	Lactamase, beta	ChEST 630n23	Gga.22380	Y	
LACTB2	Lactamase, beta 2	ChEST 578b19	Gga.9690		
LANCL1	LanC lantibiotic synthetase component C-like 1 (bacterial)	ChEST 514f16	Gga.19205	Y	
LAO	L-amino-acid oxidase precursor	ChEST 714m10	Gga.47067	Y	
LAP3	Leucine aminopeptidase 3	ChEST 652h12	Gga.2324	Y	
LARGE	Like-glycosyltransferase	ChEST 578m3	Gga.15229		
LARS	Leucyl-tRNA synthetase	ChEST 646e10	Gga.7312	Y	

LASS4	LAG1 homolog, ceramide synthase 4	ChEST 651i20	Gga.26944	Y	
LASS5	LAG1 homolog, ceramide synthase 5	ChEST 619l12	Gga.16178	Y	
LCAT	Lecithin-cholesterol acyltransferase	ChEST 729j5	Gga.3257	Y	Y
LCMT1	Leucine carboxyl methyltransferase 1	ChEST 592p15	Gga.22389	Y	
LCMT2	Leucine carboxyl methyltransferase 2	ChEST 689g16	Gga.3188	Y	
LDHA	Lactate dehydrogenase A	ChEST 648k20	Gga.4398	Y	Y
LDHB	Lactate dehydrogenase B	ChEST 520c20	Gga.4149	Y	

LFNG	LFNG O-fucosylpeptide 3-beta-N-acetylglucosaminyltransferase	ChEST 374j8	Gga.3180	Y	Y
LIAS	Lipoic acid synthetase	ChEST 622h2	Gga.9006	Y	Y
LIG3	Ligase III, DNA, ATP-dependent	ChEST 601h1	Gga.1973		
LIG4	Ligase IV, DNA, ATP-dependent	ChEST 571j4	Gga.1352	Y	
LIMK2	LIM domain kinase 2	ChEST 601b15	Gga.3222	Y	
LIPA	Lipase A, lysosomal acid, cholesterol esterase (Wolman disease)	ChEST 597i2	Gga.9879	Y	
LIPG	Lipase, endothelial	ChEST 603m20	Gga.5627	Y	Y

LIPT1	Lipoyltransferase 1	ChEST 622l16	Gga.11145		
LMF1	Lipase maturation factor 1	ChEST 695f4	Gga.5600		
LMTK2	Lemur tyrosine kinase 2	ChEST 642k14	Gga.21627	Y	
LOC395407	Fibroblast growth factor receptor-like embryonic kinase	ChEST 640c24	Gga.43782		
LOC395611	Glutathione S-transferase class-alpha	ChEST 611p18	Gga.12514	Y	
LOC395787	Smooth muscle protein phosphatase type 1-binding subunit	ChEST 620m20	Gga.41940	Y	
LOC395892	Transcribed locus, moderately similar to NP_001689.1 AU RNA-binding protein/enoyl-Coenzyme A hydratase precursor [Homo sapiens]	ChEST 571k19	Gga.44934	Y	

LOC395933	Sulfotransferase	ChEST 725j24	Gga.4124	Y	Y
LOC395990	Serine protease	ChEST 642k12	Gga.3766	Y	
LOC396300	Transcribed locus, strongly similar to NP_989962.1 adenylate cyclase 5 [Gallus gallus]	ChEST 374g20	Gga.42519	Y	
LOC396380	Glutathione transferase	ChEST 593e1	Gga.788	Y	
LOC396473	Myristoylated alanine-rich C kinase substrate (MARCKS)	ChEST 660n10	Gga.4058	Y	
LOC408038	Transcribed locus, moderately similar to NP_001013855.1 protein kinase, cGMP-dependent, type I alpha isoform [Mus musculus]	ChEST 645g11	Gga.5591	Y	
LOC415444	Similar to Solute carrier family 30 (zinc transporter), member 4	ChEST 631m3	Gga.3192	Y	Y

LOC415531	Similar to organic solute transporter beta	ChEST 600i22	Gga.11827	Y	
LOC415566	Similar to heparin/heparan sulfate:glucuronic acid C5 epimerase	ChEST 660i7	Gga.2821	Y	
LOC415611	Similar to mannosidase, alpha, class 2A, member 1	ChEST 586p16	Gga.17667	Y	
LOC415661	Transcribed locus, moderately similar to NP_006479.1 ketohEXokinase isoform b [Homo sapiens]	ChEST 625n21	Gga.43113		
LOC415663	Transcribed locus, weakly similar to NP_001015061.1 all-trans-13,14-dihydroretinol saturase [Danio rerio]	ChEST 722a7	Gga.43110		
LOC415704	Transcribed locus, moderately similar to XP_542191.2 PREDICTED: similar to Histone-lysine N-methyltransferase, H3 lysine-79 specific (Histone H3-K79 methyltransferase) (H3-K79-HMTase)	ChEST 671h19	Gga.44030	Y	
LOC415806	Similar to NAD(P) dependent steroid dehydrogenase-like	ChEST 737e22	Gga.10919	Y	

LOC415814	Similar to putative secretory pathway Ca-ATPase SPCA2	ChEST 712p6	Gga.5226	Y	
LOC415842	Similar to dipeptidase	ChEST 638l4	Gga.8230	Y	
LOC415922	Transcribed locus, moderately similar to NP_001026405.1 5'-nucleotidase, cytosolic II [Gallus gallus]	ChEST 735j23	Gga.43046	Y	Y
LOC415985	Similar to mitogen-activated protein kinase kinase kinase	ChEST 650p8	Gga.19399	Y	
LOC416083	Similar to membrane associated guanylate kinase, WW and PDZ domain containing 1	ChEST 605b7	Gga.3019		
LOC416103	Similar to tRNA-nucleotidyltransferase	ChEST 605m16	Gga.11861		
LOC416370	Similar to dimethylglycine dehydrogenase	ChEST 603i22	Gga.11193		

LOC416522	Similar to solute carrier family 5 (sodium/glucose cotransporter), member 10	ChEST 592b20	Gga.26052	Y	
LOC416580	Similar to ubiquitin-specific proteinase 31	ChEST 709d5	Gga.17579		
LOC416605	Similar to PI-3-kinase-related kinase SMG-1	ChEST 647m20	Gga.28667		
LOC416992	Similar to DEAD box RNA helicase DP97	ChEST 653d3	Gga.7050	Y	
LOC416998	Transcribed locus, weakly similar to NP_031378.1 elastase 3B preproprotein [Homo sapiens]	ChEST 615c15	Gga.46102	Y	Y
LOC416998	Transcribed locus, weakly similar to NP_031378.1 elastase 3B preproprotein [Homo sapiens]	ChEST 615c15	Gga.46102		
LOC417010	Transcribed locus, weakly similar to XP_001371109.1 PREDICTED: similar to cyclin-dependent kinase 12 [Monodelphis domestica]	ChEST 665e13	Gga.26006		

LOC417113	Similar to nitric oxide synthase 1 (neuronal) adaptor protein	ChEST 713f16	Gga.38669	Y	
LOC417209	Similar to serine/threonine kinase 7; protein kinase C-like 2	ChEST 684h3	Gga.13990	Y	
LOC417253	Similar to glutamine synthetase	ChEST 704o22	Gga.27175	Y	
LOC417281	Similar to ecto-ATP-diphosphohydrolase	ChEST 737p15	Gga.6369	Y	Y
LOC417445	Similar to MAP kinase kinase 6b	ChEST 673e19	Gga.13002	Y	
LOC418170	Similar to aldose reductase	ChEST 724i17	Gga.7815	Y	
LOC418278	Similar to Chromodomain helicase DNA binding protein 4	ChEST 620i13	Gga.11742	Y	

LOC418318	Transcribed locus, weakly similar to NP_001002700.1 fatty acid amide hydrolase 2a [Danio rerio]	ChEST 600n22	Gga.29919	Y	
LOC418344	Similar to Cdc42 GTPase-activating protein	ChEST 609f3	Gga.14005	Y	Y
LOC418356	Similar to Group X secretory phospholipase A2 precursor (Phosphatidylcholine 2-acylhydrolase GX) (GX sPLA2) (sPLA2-X)	ChEST 637o4	Gga.11447		
LOC418468	Similar to ATP-binding cassette transporter 13	ChEST 706m15	Gga.16333	Y	Y
LOC418493	Similar to hormonally upregulated Neu-associated kinase	ChEST 630c8	Gga.35805	Y	Y
LOC418549	Similar to pyridoxal kinase	ChEST 600n4	Gga.12574		
LOC418549	Similar to pyridoxal kinase	ChEST 600n4	Gga.12574		

LOC418701	Similar to Lysozyme g (1,4-beta-N-acetylmuramidase) (Goose-type lysozyme)	ChEST 691m7	Gga.31272	Y	
LOC418786	Similar to UDP-glucose ceramide glucosyltransferase-like 1	ChEST 643p21	Gga.19418	Y	
LOC418856	Similar to esterase D	ChEST 662e23	Gga.4643	Y	
LOC418878	Similar to short chain dehydrogenase/reductase	ChEST 727g5	Gga.10255	Y	
LOC418957	Similar to M-phase phosphoprotein, mpp8	ChEST 674d20	Gga.13182	Y	
LOC419011	Similar to folylpoly-gamma-glutamate carboxypeptidase	ChEST 978o15	Gga.25240	Y	
LOC419136	Similar to alpha2,3-sialyltransferase	ChEST 630i12	Gga.2239	Y	

LOC419144	Similar to mannosidase, beta A, lysosomal-like	ChEST 644o9	Gga.14436	Y	Y
LOC419425	Transcribed locus, strongly similar to NP_001025898.1 dihydrolipoamide dehydrogenase [Gallus gallus]	ChEST 701h13	Gga.44568	Y	
LOC419508	Similar to Phosphopantothenoylcysteine synthetase	ChEST 597c17	Gga.5327	Y	
LOC419508	Similar to Phosphopantothenoylcysteine synthetase	ChEST 597c17	Gga.5327		
LOC419511	Similar to Dual specificity phosphatase 11 (RNA/RNP complEX 1-interacting)	ChEST 645m23	Gga.34747	Y	Y
LOC419561	Similar to CTP synthase	ChEST 716p24	Gga.46875	Y	
LOC419565	Transcribed locus, strongly similar to XP_001235180.1 PREDICTED: similar to Cytochrome P450 4A2 precursor (CYPIVA2) (Lauric acid omega-hydroxylase) (P450-LA-omega 2) (P450	ChEST 747c9	Gga.28405	Y	Y

LOC419680	Similar to CAP, adenylate cyclase-associated protein 1 (yeast)	ChEST 592d21	Gga.42143	Y	
LOC419976	Transcribed locus, weakly similar to NP_956520.2 ribonuclease H2, subunit A [Danio rerio]	ChEST 703i3	Gga.7304	Y	Y
LOC420078	Transcribed locus, moderately similar to NP_033858.2 ATPase, class V, type 10A [Mus musculus]	ChEST 563b19	Gga.43396	Y	
LOC420078	Transcribed locus, strongly similar to XP_420085.2 PREDICTED: similar to acid alpha glucosidase, partial [Gallus gallus]	ChEST 593f5	Gga.43402	Y	
LOC420233	Similar to putative phytoene synthase	ChEST 387h21	Gga.9523		
LOC420238	Plasma glutamate carboxypeptidase	ChEST 639l17	Gga.12387	Y	Y
LOC420491	Similar to putative selenoprotein O	ChEST 645k11	Gga.7334		

LOC420553	Similar to 1-aminocyclopropane-1-carboxylate synthase	ChEST 602e13	Gga.11309	Y	
LOC420707	Transcribed locus, moderately similar to XP_001236803.1 PREDICTED: similar to RNase H, putative [Gallus gallus]	ChEST 612e14	Gga.7764	Y	
LOC421049	Similar to protein tyrosine phosphatase, receptor type, M	ChEST 729d8	Gga.25452	Y	
LOC421120	Similar to Chain A, Crystal Structure Of The Human Acyl Protein Thioesterase 1 At 1.5 A Resolution	ChEST 514m4	Gga.39257	Y	
LOC421935	Transcribed locus, moderately similar to XP_685586.2 PREDICTED: similar to phospholipase C gamma 1, partial [Danio rerio]	ChEST 666h11	Gga.23297	Y	Y
LOC421986	Similar to Protein phosphatase 1G (formerly 2C), magnesium-dependent, gamma	ChEST 563l17	Gga.34001	Y	
LOC422013	Similar to type III adenylyl cyclase	ChEST 663a11	Gga.35983		

LOC422075	Similar to GalNAc alpha 2,6-sialyltransferase	ChEST 638i12	Gga.26883	Y	
LOC422082	Similar to acid alpha glucosidase	ChEST 722f4	Gga.27713		
LOC422171	Similar to Rho GTPase activating protein 20	ChEST 698f18	Gga.19645		
LOC422179	Transcribed locus, moderately similar to NP_002602.2 pyruvate dehydrogenase kinase, isozyme 2 [Homo sapiens]	ChEST 706o9	Gga.30152	Y	
LOC422181	Similar to Phosphorylase b kinase regulatory subunit alpha, skeletal muscle isoform (Phosphorylase kinase alpha M subunit)	ChEST 697g18	Gga.30669	Y	
LOC422276	Similar to Sodium/hydrogen EXchanger 2 (Na(+)/H(+) EXchanger 2) (NHE-2) (Solute carrier family 9 member 2)	ChEST 708c3	Gga.12826	Y	Y
LOC422284	Similar to Rho-GTPase-activating protein 6 (Rho-type GTPase-activating protein RhoGAPX-1)	ChEST 514i6	Gga.48192	Y	Y

LOC422316	Similar to receptor tyrosine kinase flk-1/VEGFR-2	ChEST 608e7	Gga.43385		
LOC422448	Similar to UDP-GlcNAc:a-1,3-D -mannoside b-1,4-N-Acetylglucosaminyltransfe rase IV	ChEST 622p11	Gga.9839	Y	
LOC422497	Similar to Polo-like kinase 4 (Drosophila)	ChEST 649g12	Gga.26946	Y	
LOC422515	Transcribed locus, moderately similar to XP_001139943.1 PREDICTED: similar to aldose reductase-like peptide isoform 1 [Pan troglodytes]	ChEST 683g14	Gga.43777	Y	
LOC422527	Similar to L-3-hydroxyacyl-Coenzyme A dehydrogenase, short chain	ChEST 387l5	Gga.20511		
LOC423141	Transcribed locus, moderately similar to NP_990272.1 chromodomain helicase DNA binding protein 1 [Gallus gallus]	ChEST 676b20	Gga.37680	Y	
LOC423222	Similar to Peroxisomal acyl-coenzyme A thioester hydrolase 2a (Peroxisomal long-chain acyl-coA thioesterase 2) (ZAP128)	ChEST 592i11	Gga.27752	Y	

LOC423228	Similar to phospholipase A2, group IVB (cytosolic)	ChEST 704f18	Gga.16434	Y	
LOC423272	Similar to arginase 1	ChEST 681n19	Gga.8117	Y	
LOC423449	Similar to Cytochrome p450 46A1 (Cholesterol 24-hydroxylase)	ChEST 622o13	Gga.18483	Y	
LOC423682	Transcribed locus, weakly similar to NP_001030266.1 abhydrolase domain containing 1 [Bos taurus]	ChEST 612g19	Gga.23467		
LOC423793	Similar to M-phase phosphoprotein 1	ChEST 604g12	Gga.24014		
LOC423818	Similar to Dual specificity phosphatase 11 (RNA/RNP complEX 1-interacting)	ChEST 578g2	Gga.29457	Y	
LOC423941	Similar to serine protease	ChEST 611b2	Gga.5393		

LOC423976	Similar to pyrroline-5-carboxylate synthase	ChEST 685f14	Gga.5158	Y	
LOC424014	Similar to methyltransferase 24 (37.8 kD) (3D495)	ChEST 651a20	Gga.6263	Y	
LOC424054	Similar to GPI inositol-deacylase PGAP1	ChEST 639g12	Gga.37231	Y	
LOC424072	Aldehyde oxidase 2	ChEST 675a12	Gga.33731	Y	
LOC424109	Similar to alpha-aspartyl dipeptidase	ChEST 604h10	Gga.5219		
LOC424368	Similar to UDP-N-acteylglucosamine pyrophosphorylase 1	ChEST 648l24	Gga.25523	Y	
LOC424383	Similar to phosphatidic acid phosphatase type 2 domain containing 2	ChEST 635c14	Gga.5984	Y	

LOC424727	Transcribed locus, moderately similar to XP_001508974.1 PREDICTED: similar to gamma-carboxylase [Ornithorhynchus anatinus]	ChEST 672a11	Gga.31823		
LOC424916	Similar to organic solute transporter alpha	ChEST 729k11	Gga.35182	Y	Y
LOC424919	Similar to photolyase	ChEST 674o17	Gga.12346	Y	
LOC425015	Similar to caspase recruitment domain protein 7	ChEST 628l10	Gga.16015	Y	Y
LOC425199	Similar to cAMP phosphodiesterase PDE7	ChEST 681a2	Gga.23842		
LOC425224	Similar to pancreas-enriched phospholipase C	ChEST 677j7	Gga.5560	Y	Y
LOC425441	Similar to Sodium bicarbonate cotransporter 3 (Solute carrier family 4 member 7)	ChEST 674o7	Gga.8684		

LOC425567	Similar to Ribonucleases P/MRP protein subunit POP1 (hPOP1)	ChEST 664l7	Gga.11935	Y	
LOC425606	Similar to glutaminyl-peptide cyclotransferase-like	ChEST 609j23	Gga.28894	Y	
LOC425679	Similar to N-acetylglucosamine-6-sulfatase	ChEST 674n8	Gga.43972		
LOC425735	Similar to phosphodiesterase 4D interacting protein	ChEST 656p12	Gga.2630	Y	Y
LOC426257	Similar to cytidine deaminase	ChEST 604l13	Gga.19479	Y	
LOC426312	Similar to N-acetylglucosamine-6-sulfatase	ChEST 617i16	Gga.8948		
LOC426354	Transcribed locus, weakly similar to NP_001003978.1 G1 to S phase transition 1 [Rattus norvegicus]	ChEST 621a1	Gga.45132		

LOC426545	Similar to ADP-ribosylarginine hydrolase (ADP-ribose-L-arginine cleaving enzyme)	ChEST 746j16	Gga.11406	Y	
LOC426600	Similar to prosucrose-isomaltase	ChEST 615b9	Gga.9496		
LOC426880	Transcribed locus, strongly similar to XP_001234055.1 PREDICTED: similar to kalirin, RhoGEF kinase [Gallus gallus]	ChEST 674n24	Gga.43819	Y	
LOC426893	Similar to phospholipase A2 inhibitor gamma subunit B	ChEST 689e17	Gga.7788	Y	
LOC426988	Similar to cytokine-like nuclear factor n-pac; 3-hydroxyisobutyrate dehydrogenase-like	ChEST 628d21	Gga.41977	Y	Y
LOC427000	Similar to EXonuclease domain containing 1	ChEST 645l5	Gga.39349	Y	
LOC427123	Similar to bumetanide-sensitive Na-K-Cl cotransporter	ChEST 685k16	Gga.30091	Y	

LOC427170	Similar to Microtubule-associated serine/threonine-protein kinase 4	ChEST 670k7	Gga.25955	Y	Y
LOC427324	Similar to Vacuolar ATP synthase subunit S1 precursor (V-ATPase S1 subunit) (V-ATPase S1 accessory protein) (V-ATPase Ac45 subunit) (C7-1 protein)	ChEST 712i5	Gga.13124	Y	
LOC427414	Similar to human phosphotyrosine phosphatase kappa	ChEST 705i1	Gga.7774	Y	
LOC427469	Similar to putative repair and recombination helicase RAD26L	ChEST 689c9	Gga.39444	Y	
LOC427632	Transcribed locus, strongly similar to NP_989633.1 presenilin 2 (Alzheimer disease 4) [Gallus gallus]	ChEST 630m21	Gga.45089	Y	
LOC427638	Similar to Glutathione peroxidase 3 precursor (GSHPx-3) (GPx-3) (Plasma glutathione peroxidase) (GSHPx-P)	ChEST 520e6	Gga.4896		
LOC428279	Similar to Phospholipase C, delta 3	ChEST 634i1	Gga.28188	Y	Y

LOC428508	Transcribed locus, strongly similar to XP_419527.2 PREDICTED: similar to glutaminyl cyclase [Gallus gallus]	ChEST 659g4	Gga.43857	Y	
LOC428508	Transcribed locus, moderately similar to NP_877437.2 sulfatase modifying factor 1 [Homo sapiens]	ChEST 663o6	Gga.43849		
LOC428510	Transcribed locus, weakly similar to NP_055898.1 Cdc2-related kinase, arginine/serine-rich isoform 2 [Homo sapiens]	ChEST 602e10	Gga.43925	Y	
LOC428659	Similar to carbamoyl-phosphate synthetase 2, aspartate transcarbamylase, and dihydroorotase	ChEST 655d16	Gga.30393	Y	
LOC428660	Similar to very large inducible GTPase-1	ChEST 605i15	Gga.7800	Y	
LOC428843	Similar to Liprin-beta 2 (Protein tyrosine phosphatase receptor type f polypeptide-interacting protein-binding protein 2) (PTPRF-interacting protein-binding protein 2)	ChEST 676m17	Gga.31070	Y	
LOC428851	Similar to Fatty acid desaturase 1	ChEST 669a5	Gga.25497	Y	

LOC429096	Transcribed locus, strongly similar to XP_001236566.1 PREDICTED: similar to DNA primase (subunit p48), partial [Gallus gallus]	ChEST 374l5	Gga.43811	Y	
LOC429557	Similar to S6 kinase 2	ChEST 600n19	Gga.31767	Y	
LOC429566	Similar to nitrilase 1	ChEST 601l9	Gga.7871	Y	
LOC429567	Similar to protease	ChEST 689j4	Gga.10818	Y	
LOC429770	Transcribed locus, strongly similar to NP_001006184.1 acetoacetyl-CoA synthetase [Gallus gallus]	ChEST 724f22	Gga.44507	Y	
LOC430227	Similar to ADP-ribosyltransferase 5	ChEST 617k22	Gga.40310		
LOC430516	Similar to ligase I	ChEST 645f22	Gga.29933	Y	Y

LOC768251	Hepatic amylase pseudogene	ChEST 638m8	Gga.18470	Y	
LOC768537	Transcribed locus, moderately similar to NP_001101475.1 microtubule associated serine/threonine kinase 2 [Rattus norvegicus]	ChEST 678a5	Gga.24287	Y	
LOC768721	Transcribed locus, moderately similar to NP_001080692.1 Guanidinoacetate methyltransferase [Xenopus laevis]	ChEST 696m6	Gga.47429	Y	Y
LOC768871	Transcribed locus, moderately similar to NP_113770.2 amine oxidase, copper containing 3 [Rattus norvegicus]	ChEST 592l12	Gga.43968	Y	
LOC769030	Similar to multisynthetase complEX p38 auxiliary component	ChEST 656h13	Gga.4759	Y	
LOC769205	Transcribed locus, weakly similar to XP_001478138.1 PREDICTED: similar to Pro-Pol-dUTPase polyprotein; RNaseH; dUTPase; integrase; protease; reverse transcriptase [Mus musculus]	ChEST 683d6	Gga.44116	Y	Y
LOC769208	Similar to RNase H, putative	ChEST 643c22	Gga.40115	Y	Y

LOC769421	Similar to heparan sulfate D-glucosaminyl 3-O-sulfotransferase-3B	ChEST 387n18	Gga.40081	Y	Y
LOC769659	Similar to ornithine aminotransferase (gyrate atrophy)	ChEST 612m21	Gga.39875	Y	
LOC769674	Transcribed locus, weakly similar to XP_001512268.1 PREDICTED: similar to 11 beta-hydroxysteroid dehydrogenase type 2 [Ornithorhynchus anatinus]	ChEST 374h4	Gga.44818	Y	
LOC769713	Similar to Pro-Pol-dUTPase polyprotein; RNaseH; dUTPase; integrase; protease; reverse transcriptase	ChEST 678o7	Gga.45237	Y	
LOC769737	Similar to NAD(P)H dehydrogenase, quinone 1	ChEST 533l2	Gga.7254	Y	
LOC769755	Transcribed locus, strongly similar to XP_419672.2 PREDICTED: similar to C1-tetrahydrofolate synthase [Gallus gallus]	ChEST 593g23	Gga.44423	Y	
LOC769766	Similar to carbonic anhydrase VII	ChEST 601f3	Gga.40333	Y	

LOC769787	Similar to steroid dehydrogenase	ChEST 799e15	Gga.40097	Y	
LOC769806	Transcribed locus, weakly similar to XP_001653599.1 protein kinase c [Aedes aegypti]	ChEST 644e13	Gga.44679		
LOC769860	Similar to cAMP-specific phosphodiesterase PDE4D6	ChEST 514p11	Gga.9824	Y	
LOC769908	Transcribed locus, strongly similar to NP_990648.1 carbonic anhydrase II [Gallus gallus]	ChEST 635c20	Gga.45068	Y	
LOC769988	Similar to Methylcrotonoyl-Coenzyme A carboxylase 2 (beta)	ChEST 621g14	Gga.38005	Y	
LOC770004	Similar to carbonic anhydrase 9	ChEST 672k12	Gga.4901	Y	
LOC770129	Similar to Pro-Pol-dUTPase polyprotein; RNaseH; dUTPase; integrase; protease; reverse transcriptase	ChEST 374m24	Gga.39835	Y	

LOC770137	Transcribed locus, moderately similar to NP_001004630.1 sterol-C5-desaturase (fungal ERG3, delta-5-desaturase) homolog [Danio rerio]	ChEST 652i22	Gga.34860	Y	
LOC770137	Similar to protein phosphatase PP2A0 B subunit gamma	ChEST 635l13	Gga.34849		
LOC770340	Transcribed locus, strongly similar to XP_422126.2 PREDICTED: similar to dipeptidylpeptidase 10 [Gallus gallus]	ChEST 374e14	Gga.43420	Y	
LOC770373	Similar to inositol polyphosphate 4-phosphatase type II-alpha	ChEST 719j1	Gga.40758	Y	
LOC770392	Similar to cytosolic nucleotidase I	ChEST 620j4	Gga.30024	Y	
LOC770539	Transcribed locus, weakly similar to NP_001099909.1 solute carrier family 27 (fatty acid transporter), member 3 [Rattus norvegicus]	ChEST 374f15	Gga.23363	Y	
LOC770633	Similar to protein geranylgeranyltransferase type I, beta subunit	ChEST 571b13	Gga.8340	Y	

LOC770685	Transcribed locus, weakly similar to NP_002472.1 protein phosphatase 1, regulatory (inhibitor) subunit 12B isoform a [Homo sapiens]	ChEST 605g20	Gga.45121	Y	
LOC770738	Similar to Pro-Pol-dUTPase polyprotein; RNaseH; dUTPase; integrase; protease; reverse transcriptase	ChEST 670p23	Gga.39907		
LOC770937	Similar to ubiquinol-cytochrome c reductase complEX (7.2 kD) (ucrc)	ChEST 578k13	Gga.4600	Y	Y
LOC771012	Similar to Coagulation factor X precursor (Stuart factor) (Virus-activating protease) (VAP)	ChEST 696l23	Gga.28433	Y	
LOC771098	Similar to glutaryl-Coenzyme A dehydrogenase	ChEST 640l6	Gga.43079		
LOC771109	Similar to nuclear DNA helicase II	ChEST 593f6	Gga.26014	Y	
LOC771207	Similar to Pyridoxal (pyridoxine, vitamin B6) phosphatase	ChEST 670j3	Gga.30270		

LOC771293	Similar to urate oxidase (EC 1.7.3.3)	ChEST 720j24	Gga.40846	Y	
LOC771432	Transcribed locus, weakly similar to XP_001511896.1 PREDICTED: similar to Pancreatic triacylglycerol lipase precursor (Pancreatic lipase) (PL) [Ornithorhynchus anatinus]	ChEST 615d15	Gga.45911	Y	
LOC771702	Similar to aldolase C	ChEST 672b9	Gga.43936	Y	
LOC771753	Similar to Acyl-CoA thioesterase 2	ChEST 564m14	Gga.11254	Y	
LOC771784	Similar to phosphoseryl-tRNA kinase	ChEST 641c15	Gga.6182		
LOC771860	Transcribed locus, strongly similar to XP_416357.2 PREDICTED: similar to diacylglycerol kinase iota [Gallus gallus]	ChEST 683f18	Gga.24226	Y	
LOC771864	Similar to protein kinase BRPK	ChEST 634l24	Gga.39320	Y	

LOC771939	Similar to Ubiquinol-cytochrome c reductase hinge protein	ChEST 671o22	Gga.4648		
LOC771947	Similar to cytochrome c oxidase subunit VIIb	ChEST 605l2	Gga.16111		
LOC771974	Similar to Cytochrome P450 4A2 precursor (CYPIVA2) (Lauric acid omega-hydroxylase) (P450-LA-omega 2) (P450 K-5) (P-450 K-2)	ChEST 641h10	Gga.47408	Y	Y
LOC772047	Transcribed locus, strongly similar to XP_420537.2 PREDICTED: similar to BMP-2-inducible protein kinase (BIKe) [Gallus gallus]	ChEST 657l21	Gga.31142	Y	
LOC772062	Similar to Solute carrier family 2 (facilitated glucose transporter), member 6	ChEST 701g7	Gga.40610	Y	
LOC776060	Transcribed locus, moderately similar to NP_003636.1 solute carrier family 27 (fatty acid transporter), member 2 [Homo sapiens]	ChEST 387k20	Gga.39518	Y	Y
LOC776198	Transcribed locus, moderately similar to NP_002504.2 nucleoside-diphosphate kinase 3 [Homo sapiens]	ChEST 720d2	Gga.45828	Y	

LOC776223	Similar to Sphingomyelin phosphodiesterase, acid-like 3A	ChEST 610o2	Gga.22533	Y	
LOC776395	Similar to chromodomain helicase DNA binding protein 8	ChEST 655p5	Gga.11397	Y	
LOC776420	Transcribed locus, strongly similar to NP_001034364.1 solute carrier family 9 (sodium/hydrogen EXchanger), member 8 [Gallus gallus]	ChEST 563i13	Gga.43711	Y	
LOC776420	Transcribed locus, strongly similar to NP_001034364.1 solute carrier family 9 (sodium/hydrogen EXchanger), member 8 [Gallus gallus]	ChEST 563i13	Gga.43711		
LOC776472	Similar to Maltase-glucoamylase (alpha-glucosidase)	ChEST 615e12	Gga.45917	Y	
LOC776472	Transcribed locus, weakly similar to XP_001236721.1 PREDICTED: similar to Sucrase-isomaltase, intestinal, partial [Gallus gallus]	ChEST 615c11	Gga.45920		
LOC776574	Transcribed locus, strongly similar to NP_058797.1 peptidylprolyl isomerase A [Rattus norvegicus]	ChEST 665k17	Gga.41923	Y	Y

LOC776634	Similar to DNA-dependent protein kinase catalytic subunit	ChEST 629i1	Gga.34650	Y	
LOC776788	Transcribed locus, strongly similar to NP_005804.1 protein kinase D3 [Homo sapiens]	ChEST 707d21	Gga.24372	Y	
LOC776892	Transcribed locus, strongly similar to NP_002586.2 PCTAIRE protein kinase 2 [Homo sapiens]	ChEST 651k17	Gga.39238	Y	
LOC777042	Similar to beta-carotene 15,15-dioxygenase	ChEST 707g19	Gga.40739		
LOC777045	Transcribed locus, strongly similar to XP_427097.2 PREDICTED: similar to protein tyrosine phosphatase, partial [Gallus gallus]	ChEST 649j15	Gga.44630	Y	
LOC777136	Transcribed locus, weakly similar to NP_001083102.1 alpha2,3-sialyltransferase [Xenopus laevis]	ChEST 675m9	Gga.46918	Y	
LOC777251	Similar to Sucrase-isomaltase, intestinal	ChEST 638k23	Gga.46956	Y	

LOC777272	Similar to endothelial nitric oxide synthase	ChEST 691o23	Gga.47436	Y	
LOC777516	Transcribed locus, moderately similar to NP_001101959.1 IQ motif containing GTPase activating protein 1 [Rattus norvegicus]	ChEST 624o7	Gga.46933	Y	
LOC791224	GTP-ase like	ChEST 619o16	Gga.1075	Y	
LONP2	Lon peptidase 2, peroxisomal	ChEST 514p12	Gga.12947	Y	
LONRF1	LON peptidase N-terminal domain and ring finger 1	ChEST 564g21	Gga.40071	Y	
LONRF2	LON peptidase N-terminal domain and ring finger 2	ChEST 597g10	Gga.16883	Y	
LONRF3	LON peptidase N-terminal domain and ring finger 3	ChEST 732a24	Gga.17046		

LOX	Lysyl oxidase	ChEST 374f21	Gga.4303	Y	Y
LOXL3	Lysyl oxidase-like 3	ChEST 660b9	Gga.25372	Y	
LPGAT1	Lysophosphatidylglycerol acyltransferase 1	ChEST 611p6	Gga.22330	Y	
LPL	Lipoprotein lipase	ChEST 606n10	Gga.1152	Y	Y
LRAT	Lecithin retinol acyltransferase (phosphatidylcholine--retinol O-acyltransferase)	ChEST 617c4	Gga.12057	Y	
LRRN1	Transcribed locus, strongly similar to XP_416565.2 PREDICTED: similar to beta3-glucuronyltransferase [Gallus gallus]	ChEST 719k6	Gga.44967	Y	Y
LSG1	Large subunit GTPase 1 homolog (S. cerevisiae)	ChEST 601d9	Gga.22395		

LSM10	Transcribed locus, weakly similar to XP_001092136.1 PREDICTED: similar to solute carrier family 39 (zinc transporter), member 12 isoform 2 [Macaca mulatta]	ChEST 683f24	Gga.24982	Y	
LSS	Lanosterol synthase (2,3-oxidosqualene-lanosterol cyclase)	ChEST 625b15	Gga.42628	Y	
LTA4H	Leukotriene A4 hydrolase	ChEST 707n12	Gga.11612	Y	
LTB4DH	Leukotriene B4 12-hydroxydehydrogenase	ChEST 533k6	Gga.7516	Y	
LTC4S	Leukotriene C4 synthase	ChEST 728o5	Gga.10333	Y	Y
LYCAT	Lysocardiolipin acyltransferase	ChEST 592b6	Gga.7898	Y	
LYPLA2P1	Lysophospholipase II pseudogene 1	ChEST 597p5	Gga.43610	Y	

LYPLA3	Lysophospholipase 3 (lysosomal phospholipase A2)	ChEST 701f7	Gga.36887	Y	
LYPLAL1	Lysophospholipase-like 1	ChEST 601e24	Gga.11895	Y	
MADD	MAP-kinase activating death domain	ChEST 640f22	Gga.18988	Y	Y
MAGOH	Transcribed locus, strongly similar to XP_420470.2 PREDICTED: similar to Fraser syndrome 1 [Gallus gallus]	ChEST 734n8	Gga.44513	Y	
MAGOH	Transcribed locus, strongly similar to XP_516923.2 PREDICTED: similar to leucine zipper bearing kinase, partial [Pan troglodytes]	ChEST 640j3	Gga.44518	Y	
MAGT1	Magnesium transporter 1	ChEST 612k18	Gga.12427		
MAK10	MAK10 homolog, amino-acid N-acetyltransferase subunit, (S. cerevisiae)	ChEST 709h13	Gga.8909		

MAN1A1	Mannosidase, alpha, class 1A, member 1	ChEST 619m24	Gga.20070	Y	Y
MAN1A2	Mannosidase, alpha, class 1A, member 2	ChEST 713a20	Gga.35406		
MAN1B1	Mannosidase, alpha, class 1B, member 1	ChEST 630e22	Gga.31461		
MAN1C1	Mannosidase, alpha, class 1C, member 1	ChEST 665i12	Gga.21324	Y	
MAN2A2	Mannosidase, alpha, class 2A, member 2	ChEST 676l4	Gga.33766	Y	
MAN2B2	Mannosidase, alpha, class 2B, member 2	ChEST 610a10	Gga.21923		
MANBA	Mannosidase, beta A, lysosomal	ChEST 642c12	Gga.15982	Y	

MANEA	Mannosidase, endo-alpha	ChEST 578m21	Gga.13162	Y	
MAOA	Monoamine oxidase A	ChEST 606k1	Gga.7708	Y	Y
MAP2K1	Mitogen-activated protein kinase kinase 1	ChEST 743i7	Gga.1443	Y	Y
MAP2K1IP1	Mitogen-activated protein kinase kinase 1 interacting protein 1	ChEST 710h17	Gga.4355		
MAP2K2	Mitogen-activated protein kinase kinase 2	ChEST 602b16	Gga.2391	Y	
MAP3K1	Mitogen-activated protein kinase kinase kinase 1	ChEST 724n7	Gga.13126	Y	Y
MAP3K14	Mitogen-activated protein kinase kinase kinase 14	ChEST 612f21	Gga.8278	Y	

MAP3K2	Mitogen-activated protein kinase kinase kinase 2	ChEST 664h6	Gga.16016	Y	
MAP3K3	Mitogen-activated protein kinase kinase kinase 3	ChEST 659n16	Gga.16532	Y	
MAP3K4	Mitogen-activated protein kinase kinase kinase 4	ChEST 652e24	Gga.5490		
MAP3K5	Mitogen-activated protein kinase kinase kinase 5	ChEST 613f11	Gga.19808	Y	
MAP3K7	Mitogen-activated protein kinase kinase kinase 7	ChEST 601o13	Gga.12434		
MAP3K7IP2	Mitogen-activated protein kinase kinase kinase 7 interacting protein 2	ChEST 597h1	Gga.10217	Y	
MAP3K7IP3	Mitogen-activated protein kinase kinase kinase 7 interacting protein 3	ChEST 647l2	Gga.39939		

MAP4K4	Mitogen-activated protein kinase kinase kinase kinase 4	ChEST 709l15	Gga.22836		
MAP4K5	Mitogen-activated protein kinase kinase kinase kinase 5	ChEST 717n21	Gga.25804		
MAPK11	Mitogen-activated protein kinase 11	ChEST 635n22	Gga.7491	Y	
MAPK14	Mitogen-activated protein kinase 14	ChEST 606n4	Gga.22642	Y	Y
MAPK8	Mitogen-activated protein kinase 8	ChEST 644j20	Gga.1147	Y	Y
MAPK8IP1	Mitogen-activated protein kinase 8 interacting protein 1	ChEST 668n4	Gga.39601	Y	
MAPK8IP3	Mitogen-activated protein kinase 8 interacting protein 3	ChEST 664a13	Gga.16066		

MAPKAP1	Mitogen-activated protein kinase associated protein 1	ChEST 631a2	Gga.1093		
MAPKAPK3	Mitogen-activated protein kinase-activated protein kinase 3	ChEST 634b16	Gga.29974	Y	
MAPKAPK5	Mitogen-activated protein kinase-activated protein kinase 5	ChEST 640b16	Gga.4024	Y	
MARK1	MAP/microtubule affinity-regulating kinase 1	ChEST 629c1	Gga.12905	Y	Y
MARK3	MAP/microtubule affinity-regulating kinase 3	ChEST 621e13	Gga.17127	Y	
MASP1	Mannan-binding lectin serine peptidase 1 (C4/C2 activating component of Ra-reactive factor)	ChEST 619j18	Gga.7840	Y	
MASTL	Microtubule associated serine/threonine kinase-like	ChEST 715m22	Gga.33851	Y	

MAT1A	Methionine adenosyltransferase I, alpha	ChEST 651o2	Gga.2550		
MAT2B	Methionine adenosyltransferase II, beta	ChEST 603i18	Gga.8793	Y	
MATK	Megakaryocyte-associated tyrosine kinase	ChEST 629k18	Gga.37229	Y	Y
MBOAT5	Membrane bound O-acyltransferase domain containing 5	ChEST 710j16	Gga.19112		
MCCC1	Methylcrotonoyl-Coenzyme A carboxylase 1 (alpha)	ChEST 533p9	Gga.16102		
MCEE	Methylmalonyl CoA epimerase	ChEST 726g11	Gga.6114	Y	
MDH1	Malate dehydrogenase 1, NAD (soluble)	ChEST 611m8	Gga.1141	Y	

MERTK	C-mer proto-oncogene tyrosine kinase	ChEST 664k5	Gga.2893	Y	
METAP1	Methionyl aminopeptidase 1	ChEST 650i9	Gga.22381	Y	
METAP2	Methionyl aminopeptidase 2	ChEST 658o21	Gga.22557	Y	
METT10D	Methyltransferase 10 domain containing	ChEST 578o10	Gga.22532	Y	
METTL10	Methyltransferase like 10	ChEST 637i8	Gga.13083	Y	
METTL2B	Methyltransferase like 2B	ChEST 634j17	Gga.6308	Y	
METTL4	Methyltransferase like 4	ChEST 653l16	Gga.11304	Y	

METTL5	Methyltransferase like 5	ChEST 637n10	Gga.43955	Y	
METTL6	Methyltransferase like 6	ChEST 628j15	Gga.9726	Y	
METTL7A	Methyltransferase like 7A	ChEST 592n9	Gga.1527	Y	
METTL8	Methyltransferase like 8	ChEST 678j10	Gga.11028	Y	
METTL9	Methyltransferase like 9	ChEST 563o21	Gga.7118		
MGAT3	Mannosyl (beta-1,4-)-glycoprotein beta-1,4-N-acetylglucosaminyltransferase	ChEST 635n21	Gga.1768	Y	
MGAT4B	Mannosyl (alpha-1,3-)-glycoprotein beta-1,4-N-acetylglucosaminyltransferase, isozyme B	ChEST 605i20	Gga.8069	Y	Y

MGAT5	Mannosyl (alpha-1,6-)-glycoprotein beta-1,6-N-acetyl-glucosaminyltransferase	ChEST 699m12	Gga.14693	Y	
MGLL	Monoglyceride lipase	ChEST 514c19	Gga.14590	Y	
MGMT	O-6-methylguanine-DNA methyltransferase	ChEST 564a22	Gga.6353	Y	
MGST1	Microsomal glutathione S-transferase 1	ChEST 610g12	Gga.38109		
MGST3	Microsomal glutathione S-transferase 3	ChEST 647j3	Gga.39569	Y	
MKNK1	MAP kinase interacting serine/threonine kinase 1	ChEST 707d3	Gga.30333	Y	
MLYCD	Malonyl-CoA decarboxylase	ChEST 615b15	Gga.16747		

MME	Membrane metallo-endopeptidase	ChEST 592h24	Gga.42635	Y	
MMP16	Matrix metallopeptidase 16 (membrane-inserted)	ChEST 675o4	Gga.2098	Y	
MMP17	Matrix metallopeptidase 17 (membrane-inserted)	ChEST 586e12	Gga.39767	Y	Y
MMP2	Matrix metallopeptidase 2 (gelatinase A, 72kDa gelatinase, 72kDa type IV collagenase)	ChEST 612i1	Gga.3199	Y	
MMP23B	Matrix metallopeptidase 23B	ChEST 641d1	Gga.12185	Y	
MMP24	Matrix metallopeptidase 24 (membrane-inserted)	ChEST 718c13	Gga.26865	Y	
MMP3	Matrix metallopeptidase 3 (stromelysin 1, progelatinase)	ChEST 605a18	Gga.12800	Y	

MMP9	Matrix metallopeptidase 9 (gelatinase B, 92kDa gelatinase, 92kDa type IV collagenase)	ChEST 681k8	Gga.198		
MOGAT2	Monoacylglycerol O-acyltransferase 2	ChEST 638d20	Gga.29687	Y	Y
MOSC2	MOCO sulphurase C-terminal domain containing 2	ChEST 605i13	Gga.4936	Y	Y
MOXD1	Monooxygenase, DBH-like 1	ChEST 514j24	Gga.969	Y	Y
MPHOSPH10	M-phase phosphoprotein 10 (U3 small nucleolar ribonucleoprotein)	ChEST 593i20	Gga.9219	Y	
MPHOSPH6	M-phase phosphoprotein 6	ChEST 638g7	Gga.9575	Y	
MPHOSPH9	M-phase phosphoprotein 9	ChEST 699j14	Gga.12537	Y	

MPI	Mannose phosphate isomerase	ChEST 619b11	Gga.4708	Y	Y
MPO	Myeloperoxidase	ChEST 697e11	Gga.23215	Y	
MPPE1	Metallophosphoesterase 1	ChEST 662m6	Gga.27147	Y	
MPPED1	Metallophosphoesterase domain containing 1	ChEST 643h5	Gga.15829	Y	Y
MPST	Mercaptopyruvate sulfurtransferase	ChEST 615a5	Gga.11582		
M-RIP	Myosin phosphatase-Rho interacting protein	ChEST 659b13	Gga.13435	Y	
MSI2	Transcribed locus, strongly similar to NP_001015006.1 DNA methyltransferase 1-associated protein 1 [Rattus norvegicus]	ChEST 738m18	Gga.29156		

MSRA	Methionine sulfoxide reductase A	ChEST 622g18	Gga.44234	Y	Y
MSRB3	Methionine sulfoxide reductase B3	ChEST 514h15	Gga.10617	Y	Y
MST1R	Macrophage stimulating 1 receptor (c-met-related tyrosine kinase)	ChEST 631e2	Gga.29675		
MT3	Metallothionein 3	ChEST 696e4	Gga.20311	Y	
MTHFD1	Methylenetetrahydrofolate dehydrogenase (NADP+ dependent) 1, methenyltetrahydrofolate cyclohydrolase, formyltetrahydrofolate synthetase	ChEST 645d1	Gga.1080	Y	Y
MTHFD1L	Methylenetetrahydrofolate dehydrogenase (NADP+ dependent) 1-like	ChEST 664i13	Gga.43463	Y	
MTHFR	5,10-methylenetetrahydrofolate reductase (NADPH)	ChEST 651e3	Gga.29978	Y	

MTHFS	5,10-methenyltetrahydrofolate synthetase (5-formyltetrahydrofolate cyclo-ligase)	ChEST 727j20	Gga.7827		
MTHFSD	Methenyltetrahydrofolate synthetase domain containing	ChEST 514b12	Gga.12382	Y	
MTRR	5-methyltetrahydrofolate-homocysteine methyltransferase reductase	ChEST 664m23	Gga.10838	Y	
MUSK	Muscle, skeletal, receptor tyrosine kinase	ChEST 799i6	Gga.123	Y	Y
MUT	Methylmalonyl Coenzyme A mutase	ChEST 638g20	Gga.33981	Y	
MVD	Mevalonate (diphospho) decarboxylase	ChEST 703o7	Gga.35377		
MVK	Mevalonate kinase (mevalonic aciduria)	ChEST 624e13	Gga.30821	Y	

MYO3B	Transcribed locus, strongly similar to NP_004068.2 citrate synthase precursor, isoform a [Homo sapiens]	ChEST 602b21	Gga.34275	Y	
MYST3	MYST histone acetyltransferase (monocytic leukemia) 3	ChEST 592p6	Gga.39044	Y	
MYST4	MYST histone acetyltransferase (monocytic leukemia) 4	ChEST 374o4	Gga.12116	Y	
N6AMT1	N-6 adenine-specific DNA methyltransferase 1 (putative)	ChEST 603c11	Gga.1485		
N6AMT2	N-6 adenine-specific DNA methyltransferase 2 (putative)	ChEST 533d19	Gga.1623	Y	Y
NADK	NAD kinase	ChEST 604b20	Gga.14395		
NADSYN1	NAD synthetase 1	ChEST 645h22	Gga.22360	Y	

NAGA	N-acetylgalactosaminidase, alpha-	ChEST 593i15	Gga.4369		
NAGLU	N-acetylglucosaminidase, alpha- (Sanfilippo disease IIIB)	ChEST 715e20	Gga.28095	Y	
NAGPA	N-acetylglucosamine-1-phosphodiester alpha-N-acetylglucosaminidase	ChEST 627i19	Gga.23279	Y	
NANS	N-acetylneuraminic acid synthase (sialic acid synthase)	ChEST 635a24	Gga.8814	Y	
NAPRT1	Nicotinate phosphoribosyltransferase domain containing 1	ChEST 673i10	Gga.11834	Y	
NARS	Asparaginyl-tRNA synthetase	ChEST 646k14	Gga.10023	Y	
NAT1	N-acetyltransferase 1 (arylamine N-acetyltransferase)	ChEST 799p19	Gga.746	Y	Y

NAT10	N-acetyltransferase 10	ChEST 716j15	Gga.15786	Y	Y
NAT12	N-acetyltransferase 12	ChEST 711l21	Gga.13904	Y	
NAT13	N-acetyltransferase 13	ChEST 563i1	Gga.8828	Y	
NAT5	N-acetyltransferase 5	ChEST 564m21	Gga.34928	Y	
NAT8L	N-acetyltransferase 8-like	ChEST 699d5	Gga.12727	Y	
NAT9	N-acetyltransferase 9	ChEST 634b1	Gga.34581	Y	
NBR1	Transcribed locus, moderately similar to XP_001521647.1 PREDICTED: similar to putative protein kinase NY-REN-64 antigen, partial [Ornithorhynchus anatinus]	ChEST 715h19	Gga.42780		

NCF2	Neutrophil cytosolic factor 2 (65kDa, chronic granulomatous disease, autosomal 2)	ChEST 678i15	Gga.34552	Y	
NDOR1	NADPH dependent diflavin oxidoreductase 1	ChEST 564p22	Gga.29755	Y	
NDP	Norrie disease (pseudoglioma)	ChEST 533p17	Gga.11766	Y	Y
NDST1	N-deacetylase/N-sulfotransferase (heparan glucosaminyl) 1	ChEST 387n11	Gga.27246	Y	
NDST2	N-deacetylase/N-sulfotransferase (heparan glucosaminyl) 2	ChEST 597e12	Gga.8268	Y	Y
NDST2	N-deacetylase/N-sulfotransferase (heparan glucosaminyl) 2	ChEST 597e12	Gga.8268	Y	Y
NDST3	N-deacetylase/N-sulfotransferase (heparan glucosaminyl) 3	ChEST 659n6	Gga.15685	Y	

NDUFA10	NADH dehydrogenase (ubiquinone) 1 alpha subcomplEX, 10, 42kDa	ChEST 652d5	Gga.17230	Y	
NDUFA11	NADH dehydrogenase (ubiquinone) 1 alpha subcomplEX, 11, 14.7kDa	ChEST 593f21	Gga.1735	Y	
NDUFA12	NADH dehydrogenase (ubiquinone) 1 alpha subcomplEX, 12	ChEST 564m7	Gga.7183	Y	Y
NDUFA4	NADH dehydrogenase (ubiquinone) 1 alpha subcomplEX, 4, 9kDa	ChEST 606m22	Gga.4526	Y	Y
NDUFA5	NADH dehydrogenase (ubiquinone) 1 alpha subcomplEX, 5, 13kDa	ChEST 600n17	Gga.6336	Y	
NDUFA6	NADH dehydrogenase (ubiquinone) 1 alpha subcomplEX, 6, 14kDa	ChEST 603g16	Gga.2235	Y	
NDUFA7	NADH dehydrogenase (ubiquinone) 1 alpha subcomplEX, 7, 14.5kDa	ChEST 698d22	Gga.6088	Y	

NDUFA9	NADH dehydrogenase (ubiquinone) 1 alpha subcomplEX, 9, 39kDa	ChEST 624h24	Gga.1626	Y	
NDUFAB1	NADH dehydrogenase (ubiquinone) 1, alpha/beta subcomplEX, 1, 8kDa	ChEST 600n10	Gga.2627		
NDUFAF1	NADH dehydrogenase (ubiquinone) 1 alpha subcomplEX, assembly factor 1	ChEST 647l7	Gga.12267		
NDUFB1	NADH dehydrogenase (ubiquinone) 1 beta subcomplEX, 1, 7kDa	ChEST 652d11	Gga.4746	Y	
NDUFB10	NADH dehydrogenase (ubiquinone) 1 beta subcomplEX, 10, 22kDa	ChEST 620f17	Gga.11296	Y	Y
NDUFB2	NADH dehydrogenase (ubiquinone) 1 beta subcomplEX, 2, 8kDa	ChEST 639g19	Gga.43134	Y	
NDUFB4	NADH dehydrogenase (ubiquinone) 1 beta subcomplEX, 4, 15kDa	ChEST 628j24	Gga.39013	Y	

NDUFB6	NADH dehydrogenase (ubiquinone) 1 beta subcomplEX, 6, 17kDa	ChEST 624m11	Gga.8285		
NDUFB8	NADH dehydrogenase (ubiquinone) 1 beta subcomplEX, 8, 19kDa	ChEST 612k21	Gga.8822		
NDUFB9	NADH dehydrogenase (ubiquinone) 1 beta subcomplEX, 9, 22kDa	ChEST 609a12	Gga.13245		
NDUFC2	NADH dehydrogenase (ubiquinone) 1, subcomplEX unknown, 2, 14.5kDa	ChEST 640a22	Gga.5843		
NDUFS3	NADH dehydrogenase (ubiquinone) Fe-S protein 3, 30kDa (NADH-coenzyme Q reductase)	ChEST 652k7	Gga.1290		
NDUFS4	NADH dehydrogenase (ubiquinone) Fe-S protein 4, 18kDa (NADH-coenzyme Q reductase)	ChEST 533e8	Gga.22180	Y	Y
NDUFS5	NADH dehydrogenase (ubiquinone) Fe-S protein 5, 15kDa (NADH-coenzyme Q reductase)	ChEST 514c2	Gga.12624	Y	Y

NDUFS6	NADH dehydrogenase (ubiquinone) Fe-S protein 6, 13kDa (NADH-coenzyme Q reductase)	ChEST 978o24	Gga.8284	Y	
NDUFS8	NADH dehydrogenase (ubiquinone) Fe-S protein 8, 23kDa (NADH-coenzyme Q reductase)	ChEST 564i2	Gga.34750	Y	
NDUFV1	NADH dehydrogenase (ubiquinone) flavoprotein 1, 51kDa	ChEST 600a19	Gga.42010	Y	Y
NDUFV2	NADH dehydrogenase (ubiquinone) flavoprotein 2, 24kDa	ChEST 609p14	Gga.4778	Y	
NDUFV3	NADH dehydrogenase (ubiquinone) flavoprotein 3, 10kDa	ChEST 746o9	Gga.29746	Y	
NEK3	NIMA (never in mitosis gene a)-related kinase 3	ChEST 642f18	Gga.43546	Y	
NEK4	NIMA (never in mitosis gene a)-related kinase 4	ChEST 726c21	Gga.5384	Y	Y

NEK9	NIMA (never in mitosis gene a)- related kinase 9	ChEST 634e6	Gga.4667	Y	Y
NEU3	Sialidase 3 (membrane sialidase)	ChEST 578i9	Gga.30866	Y	Y
NGLY1	N-glycanase 1	ChEST 602o13	Gga.22517		
NIT2	Nitrilase family, member 2	ChEST 600l24	Gga.21371	Y	
NKAIN3	Na+/K+ transporting ATPase interacting 3	ChEST 676o9	Gga.9681	Y	
NKAIN4	Na+/K+ transporting ATPase interacting 4	ChEST 650j18	Gga.10033	Y	Y
NLK	Nemo-like kinase	ChEST 697k11	Gga.11987		

NLN	Neurolysin (metallopeptidase M3 family)	ChEST 648i20	Gga.13207	Y	
NMT1	N-myristoyltransferase 1	ChEST 713l3	Gga.11792	Y	Y
NMT2	N-myristoyltransferase 2	ChEST 652h10	Gga.9785		
NOTUM	Notum pectinacetylesterase homolog (Drosophila)	ChEST 691c13	Gga.19655	Y	
NOX1	NADPH oxidase 1	ChEST 638e23	Gga.31441	Y	Y
NOX5	NADPH oxidase, EF-hand calcium binding domain 5	ChEST 602j6	Gga.40313	Y	
NOX5	NADPH oxidase, EF-hand calcium binding domain 5	ChEST 602j6	Gga.40313		

NOXA1	NADPH oxidase activator 1	ChEST 731i19	Gga.29754	Y	Y
NPC1	Niemann-Pick disease, type C1	ChEST 578a24	Gga.23934		
NPC2	Niemann-Pick disease, type C2	ChEST 649h22	Gga.4677		
NPEPL1	Aminopeptidase-like 1	ChEST 374p21	Gga.25589		
NPEPPS	Aminopeptidase puromycin sensitive	ChEST 652m20	Gga.13539	Y	
NPL	N-acetylneuraminate pyruvate lyase (dihydrodipicolinate synthase)	ChEST 678l14	Gga.42622	Y	
NPNT	Transcribed locus, moderately similar to NP_003240.1 thimet oligopeptidase 1 [Homo sapiens]	ChEST 637g14	Gga.43405	Y	

NQO2	NAD(P)H dehydrogenase, quinone 2	ChEST 608n18	Gga.11743	Y	
NRD1	Nardilysin (N-arginine dibasic convertase)	ChEST 563e5	Gga.8172	Y	
NSDHL	NAD(P) dependent steroid dehydrogenase-like	ChEST 601f19	Gga.9949	Y	
NSMAF	Neutral sphingomyelinase (N-SMase) activation associated factor	ChEST 608l10	Gga.10577		
NT5C1B	5'-nucleotidase, cytosolic IB	ChEST 604g6	Gga.14492		
NT5C2	5'-nucleotidase, cytosolic II	ChEST 650n12	Gga.22448	Y	
NT5C3	5'-nucleotidase, cytosolic III	ChEST 622p8	Gga.5031	Y	

NT5C3L	5'-nucleotidase, cytosolic III-like	ChEST 603b10	Gga.4321	Y	
NT5C3L	5'-nucleotidase, cytosolic III-like	ChEST 603b10	Gga.4321		
NT5DC1	5'-nucleotidase domain containing 1	ChEST 635l14	Gga.12224	Y	
NT5DC2	5'-nucleotidase domain containing 2	ChEST 606b11	Gga.6090		
NT5E	5'-nucleotidase, ecto (CD73)	ChEST 824n21	Gga.31825	Y	Y
NTAN1	N-terminal asparagine amidase	ChEST 640l23	Gga.3121	Y	
NTRK1	Neurotrophic tyrosine kinase, receptor, type 1	ChEST 664j15	Gga.579	Y	

NTRK2	Neurotrophic tyrosine kinase, receptor, type 2	ChEST 739f10	Gga.681	Y	Y
NUAK1	NUAK family, SNF1-like kinase, 1	ChEST 718n12	Gga.34763	Y	Y
NUCKS1	Nuclear casein kinase and cyclin-dependent kinase substrate 1	ChEST 671o21	Gga.42383	Y	
NUS1	Nuclear undecaprenyl pyrophosphate synthase 1 homolog (S. cerevisiae)	ChEST 799h23	Gga.16764	Y	
OAT	Ornithine aminotransferase	ChEST 608e2	Gga.12529	Y	Y
OAZ1	Ornithine decarboxylase antizyme 1	ChEST 563e11	Gga.24988	Y	
OAZ2	Similar to ornithine decarboxylase antizyme 2	ChEST 646d11	Gga.33532	Y	Y

ODC1	Ornithine decarboxylase 1	ChEST 603c6	Gga.4340	Y	
OGDHL	Oxoglutarate dehydrogenase-like	ChEST 662h10	Gga.24669	Y	
OGFOD1	2-oxoglutarate and iron-dependent oxygenase domain containing 1	ChEST 533f1	Gga.31213		
OGFRL1	Transcribed locus, strongly similar to XP_001233469.1 PREDICTED: similar to protein tyrosine phosphatase, non-receptor type 14 isoform 1 [Gallus gallus]	ChEST 668b22	Gga.24199	Y	
OGG1	8-oxoguanine DNA glycosylase	ChEST 604c23	Gga.11806	Y	
OGT	O-linked N-acetylglucosamine (GlcNAc) transferase (UDP-N-acetylglucosamine:polypeptide-N-acetylglucosaminyl transferase)	ChEST 387o3	Gga.41951	Y	
OLA1	Obg-like ATPase 1	ChEST 714o4	Gga.1056	Y	

OMA1	OMA1 homolog, zinc metallopeptidase (S. cerevisiae)	ChEST 662h3	Gga.13076	Y	
ORAI1	ORAI calcium release-activated calcium modulator 1	ChEST 666b17	Gga.22197	Y	
ORAI2	ORAI calcium release-activated calcium modulator 2	ChEST 653c12	Gga.2376	Y	
OSGEPL1	O-sialoglycoprotein endopeptidase-like 1	ChEST 634p12	Gga.5350	Y	
OVCH2	Ovochymase 2	ChEST 629g9	Gga.40314	Y	
OXCT1	3-oxoacid CoA transferase 1	ChEST 571p7	Gga.17826	Y	Y
OXNAD1	Oxidoreductase NAD-binding domain containing 1	ChEST 374d7	Gga.12035	Y	Y

P4HA1	Procollagen-proline, 2-oxoglutarate 4-dioxygenase (proline 4-hydroxylase), alpha polypeptide I	ChEST 609h13	Gga.17975	Y	Y
P4HA2	Procollagen-proline, 2-oxoglutarate 4-dioxygenase (proline 4-hydroxylase), alpha polypeptide II	ChEST 601b12	Gga.5799	Y	Y
P4HA3	Procollagen-proline, 2-oxoglutarate 4-dioxygenase (proline 4-hydroxylase), alpha polypeptide III	ChEST 644n23	Gga.16019	Y	Y
PAAF1	Proteasomal ATPase-associated factor 1	ChEST 638k20	Gga.22242	Y	
PACSIN2	Protein kinase C and casein kinase substrate in neurons 2	ChEST 613h17	Gga.3831		
PACSIN3	Protein kinase C and casein kinase substrate in neurons 3	ChEST 665k16	Gga.12120	Y	Y
PAFAH1B1	Platelet-activating factor acetylhydrolase, isoform Ib, alpha subunit 45kDa	ChEST 514c4	Gga.11892	Y	

PAFAH1B2	Platelet-activating factor acetylhydrolase, isoform Ib, beta subunit 30kDa	ChEST 563d6	Gga.17363	Y	Y
PAFAH2	Similar to platelet-activating factor acetylhydrolase 2	ChEST 730j10	Gga.9619	Y	Y
PAH	Phenylalanine hydroxylase	ChEST 732d5	Gga.12887	Y	Y
PAICS	Phosphoribosylaminoimidazole carboxylase, phosphoribosylaminoimidazole succinocarboxamide synthetase	ChEST 609o24	Gga.3058		
PAK1	P21/Cdc42/Rac1-activated kinase 1 (STE20 homolog, yeast)	ChEST 641n12	Gga.31573	Y	
PAK2	P21 (CDKN1A)-activated kinase 2	ChEST 597h19	Gga.7822	Y	
PAK4	P21(CDKN1A)-activated kinase 4	ChEST 617c21	Gga.13850		

PAK7	P21(CDKN1A)-activated kinase 7	ChEST 705o8	Gga.15200	Y	
PAM	Peptidylglycine alpha-amidating monooxygenase	ChEST 533p14	Gga.17282	Y	Y
PAN3	PAN3 polyA specific ribonuclease subunit homolog (S. cerevisiae)	ChEST 603j1	Gga.18898	Y	
PANK1	Pantothenate kinase 1	ChEST 697k12	Gga.20562	Y	
PANK2	Pantothenate kinase 2 (Hallervorden-Spatz syndrome)	ChEST 737a6	Gga.35670	Y	
PAPSS2	3'-phosphoadenosine 5'-phosphosulfate synthase 2	ChEST 606c6	Gga.47276	Y	Y
PARG	Poly (ADP-ribose) glycohydrolase	ChEST 611p21	Gga.36320		

PARK7	Parkinson disease (autosomal recessive, early onset) 7	ChEST 387a17	Gga.3836	Y	
PBK	PDZ binding kinase	ChEST 642j17	Gga.11746	Y	
PCBD1	Pterin-4 alpha-carbinolamine dehydratase/dimerization cofactor of hepatocyte nuclear factor 1 alpha (TCF1)	ChEST 722b3	Gga.427	Y	
PCBD2	Pterin-4 alpha-carbinolamine dehydratase/dimerization cofactor of hepatocyte nuclear factor 1 alpha (TCF1) 2	ChEST 674o18	Gga.181		
PCCA	Propionyl Coenzyme A carboxylase, alpha polypeptide	ChEST 564e3	Gga.26686	Y	Y
PCCB	Propionyl Coenzyme A carboxylase, beta polypeptide	ChEST 653l10	Gga.4089	Y	
PCMT1	Protein-L-isoaspartate (D-aspartate) O-methyltransferase	ChEST 657h23	Gga.16623	Y	Y

PCMTD1	Protein-L-isoaspartate (D-aspartate) O-methyltransferase domain containing 1	ChEST 651c5	Gga.21457	Y	
PCMTD2	Protein-L-isoaspartate (D-aspartate) O-methyltransferase domain containing 2	ChEST 634j1	Gga.12331	Y	
PCSK2	Proprotein convertase subtilisin/kEXin type 2	ChEST 611n2	Gga.9404	Y	
PCSK5	Proprotein convertase subtilisin/kEXin type 5	ChEST 608b12	Gga.12660	Y	
PCSK6	Proprotein convertase subtilisin/kEXin type 6	ChEST 597l19	Gga.21090		
PCSK7	Proprotein convertase subtilisin/kEXin type 7	ChEST 602f19	Gga.5311	Y	
PCTK2	PCTAIRE protein kinase 2	ChEST 602j9	Gga.42341		

PCYOX1	Prenylcysteine oxidase 1	ChEST 609o19	Gga.6140		
PCYOX1L	Prenylcysteine oxidase 1 like	ChEST 533i14	Gga.31499	Y	
PDDC1	Parkinson disease 7 domain containing 1	ChEST 619l10	Gga.9696	Y	
PDE10A	Phosphodiesterase 10A	ChEST 668a23	Gga.27969		
PDE11A	Phosphodiesterase 11A	ChEST 709f12	Gga.10719	Y	
PDE1A	Phosphodiesterase 1A, calmodulin-dependent	ChEST 681l5	Gga.13812	Y	
PDE3B	Phosphodiesterase 3B, cGMP-inhibited	ChEST 652f23	Gga.3624		

PDE4B	Phosphodiesterase 4B, cAMP-specific (phosphodiesterase E4 dunce homolog, Drosophila)	ChEST 643p10	Gga.19233	Y	
PDE5A	Phosphodiesterase 5A, cGMP-specific	ChEST 689k7	Gga.14226	Y	Y
PDE7B	Phosphodiesterase 7B	ChEST 648n24	Gga.37525	Y	
PDE8A	Phosphodiesterase 8A	ChEST 611i3	Gga.30112		
PDE9A	Phosphodiesterase 9A	ChEST 571l7	Gga.10055		
PDHA1	Pyruvate dehydrogenase (lipoamide) alpha 1	ChEST 597m3	Gga.2052	Y	Y
PDHB	Pyruvate dehydrogenase (lipoamide) beta	ChEST 600b18	Gga.9380		

PDIA3	Protein disulfide isomerase family A, member 3	ChEST 606p21	Gga.3802	Y	Y
PDIA4	Protein disulfide isomerase family A, member 4	ChEST 643k13	Gga.2205	Y	Y
PDIA5	Protein disulfide isomerase family A, member 5	ChEST 639c15	Gga.12934	Y	
PDIA6	Protein disulfide isomerase family A, member 6	ChEST 578i14	Gga.20145	Y	
PDK3	Pyruvate dehydrogenase kinase, isozyme 3	ChEST 739g16	Gga.21933	Y	Y
PDSS2	Prenyl (decaprenyl) diphosphate synthase, subunit 2	ChEST 978i22	Gga.44784	Y	
PDXDC1	Pyridoxal-dependent decarboxylase domain containing 1	ChEST 723f22	Gga.30019	Y	Y

PDXDC1	Transcribed locus, strongly similar to NP_940933.3 ATPase, class II, type 9B [Homo sapiens]	ChEST 602c21	Gga.30020	Y	
PECI	Peroxisomal D3,D2-enoyl-CoA isomerase	ChEST 593c13	Gga.11390	Y	
PECR	Peroxisomal trans-2-enoyl-CoA reductase	ChEST 592e15	Gga.22449		
PEMT	Phosphatidylethanolamine N-methyltransferase	ChEST 604k11	Gga.8004	Y	
PEPD	Peptidase D	ChEST 563n1	Gga.5853	Y	
PFKFB3	6-phosphofructo-2-kinase/fructose-2,6-biphosphatase 3	ChEST 602c7	Gga.34514	Y	
PFKL	Phosphofructokinase, liver	ChEST 642j20	Gga.2810	Y	Y

PGAM1	Phosphoglycerate mutase 1 (brain)	ChEST 387j10	Gga.6033	Y	Y
PGAM5	Phosphoglycerate mutase family member 5	ChEST 604h14	Gga.42225	Y	
PGD	Phosphogluconate dehydrogenase	ChEST 641h7	Gga.1282	Y	
PGDS	Prostaglandin-D synthase	ChEST 640o19	Gga.3137	Y	Y
PGK1	Phosphoglycerate kinase 1	ChEST 613o23	Gga.8606		
PGM1	Phosphoglucomutase 1	ChEST 601b4	Gga.33728		
PGM2L1	Phosphoglucomutase 2-like 1	ChEST 634d17	Gga.22261	Y	

PGM3	Phosphoglucomutase 3	ChEST 533m3	Gga.42726	Y	
PGM5	Phosphoglucomutase 5	ChEST 514g7	Gga.31282	Y	Y
PGS1	Phosphatidylglycerophosphate synthase 1	ChEST 578h17	Gga.22573	Y	Y
PHACTR1	Phosphatase and actin regulator 1	ChEST 563h14	Gga.8433	Y	Y
PHGDH	Phosphoglycerate dehydrogenase	ChEST 675a22	Gga.7837		
PHKB	Phosphorylase kinase, beta	ChEST 650i4	Gga.27645	Y	
PHKG1	Phosphorylase kinase, gamma 1 (muscle)	ChEST 657k24	Gga.6022	Y	

PHLPP	PH domain and leucine rich repeat protein phosphatase	ChEST 571d8	Gga.19253	Y	
PHLPPL	PH domain and leucine rich repeat protein phosphatase-like	ChEST 659l21	Gga.26763	Y	
PHOSPHO1	Phosphatase, orphan 1	ChEST 638p3	Gga.2334	Y	Y
PHOSPHO2	Phosphatase, orphan 2	ChEST 671n17	Gga.11690	Y	Y
PHPT1	Phosphohistidine phosphatase 1	ChEST 655h17	Gga.9876	Y	
PHPT1	Phosphohistidine phosphatase 1	ChEST 586n6	Gga.4609	Y	
PHYH	Phytanoyl-CoA 2-hydroxylase	ChEST 729i23	Gga.13495	Y	

PHYHD1	Phytanoyl-CoA dioxygenase domain containing 1	ChEST 533p12	Gga.30850	Y	
PHYHIPL	Phytanoyl-CoA 2-hydroxylase interacting protein-like	ChEST 738c22	Gga.21820	Y	
PI4K2A	Phosphatidylinositol 4-kinase type 2 alpha	ChEST 746l23	Gga.23371	Y	
PI4K2B	Phosphatidylinositol 4-kinase type 2 beta	ChEST 650m21	Gga.20611	Y	
PI4KA	Phosphatidylinositol 4-kinase, catalytic, alpha	ChEST 652b15	Gga.29066	Y	
PIF1	PIF1 5'-to-3' DNA helicase homolog (S. cerevisiae)	ChEST 659a11	Gga.16531	Y	
PIK3AP1	Phosphoinositide-3-kinase adaptor protein 1	ChEST 634o1	Gga.11560	Y	

PIK3C2A	Phosphoinositide-3-kinase, class 2, alpha polypeptide	ChEST 622i4	Gga.42850	Y	
PIK3C2B	Phosphoinositide-3-kinase, class 2, beta polypeptide	ChEST 646j17	Gga.7643	Y	
PIK3C2G	Phosphoinositide-3-kinase, class 2, gamma polypeptide	ChEST 664c23	Gga.24803		
PIK3C2G	Phosphoinositide-3-kinase, class 2, gamma polypeptide	ChEST 664c23	Gga.24803		
PIK3CD	Phosphoinositide-3-kinase, catalytic, delta polypeptide	ChEST 691f11	Gga.8789	Y	
PIK3R1	Phosphoinositide-3-kinase, regulatory subunit 1 (p85 alpha)	ChEST 609o21	Gga.18018	Y	
PIK3R2	Phosphoinositide-3-kinase, regulatory subunit 2 (p85 beta)	ChEST 659o6	Gga.34354	Y	

PIK3R4	Phosphoinositide-3-kinase, regulatory subunit 4, p150	ChEST 602d14	Gga.30898		
PIK3R4	Phosphoinositide-3-kinase, regulatory subunit 4, p150	ChEST 602d14	Gga.30898		
PIK3R5	Phosphoinositide-3-kinase, regulatory subunit 5, p101	ChEST 824m14	Gga.39258	Y	
PIK3R6	Phosphoinositide-3-kinase, regulatory subunit 6	ChEST 617e16	Gga.30518		
PIN4	Protein (peptidylprolyl cis/trans isomerase) NIMA-interacting, 4 (parvulin)	ChEST 662n22	Gga.7537	Y	
PINK1	PTEN induced putative kinase 1	ChEST 667i11	Gga.7826	Y	
PIP4K2A	Phosphatidylinositol-5-phosphate 4-kinase, type II, alpha	ChEST 703c15	Gga.22009	Y	

PIP5K1C	Phosphatidylinositol-4-phosphate 5-kinase, type I, gamma	ChEST 714p21	Gga.24426		
PISD	Phosphatidylserine decarboxylase	ChEST 640c17	Gga.9957	Y	
PITRM1	Pitrilysin metallopeptidase 1	ChEST 571k8	Gga.2002	Y	
PKD2	Polycystic kidney disease 2 (autosomal dominant)	ChEST 563a18	Gga.6054	Y	
PKD2L2	Transcribed locus, weakly similar to XP_542191.2 PREDICTED: similar to Histone-lysine N-methyltransferase, H3 lysine-79 specific (Histone H3-K79 methyltransferase) (H3-K79-HMTase)	ChEST 660c19	Gga.29400	Y	
PKHD1	Polycystic kidney and hepatic disease 1 (autosomal recessive)	ChEST 592p19	Gga.11026	Y	
PKM2	Pyruvate kinase, muscle	ChEST 612m11	Gga.4299	Y	

PKN2	Protein kinase N2	ChEST 601k12	Gga.8657	Y	Y
PLA1A	Phospholipase A1 member A	ChEST 650e13	Gga.31437	Y	
PLA2G12A	Phospholipase A2, group XIIA	ChEST 681a21	Gga.19331	Y	
PLA2G12B	Phospholipase A2, group XIIB	ChEST 716f9	Gga.24458	Y	Y
PLA2G1B	Phospholipase A2, group IB (pancreas)	ChEST 615k10	Gga.6214	Y	
PLA2G4A	Phospholipase A2, group IVA (cytosolic, calcium-dependent)	ChEST 695o14	Gga.3723		
PLA2G6	Phospholipase A2, group VI (cytosolic, calcium-independent)	ChEST 533m4	Gga.9267	Y	

PLA2G7	Phospholipase A2, group VII (platelet-activating factor acetylhydrolase, plasma)	ChEST 609o7	Gga.42432	Y	
PLA2R1	Phospholipase A2 receptor 1, 180kDa	ChEST 724b16	Gga.12884		
PLAA	Phospholipase A2-activating protein	ChEST 646n6	Gga.39236		
PLB1	Phospholipase B1	ChEST 703k17	Gga.29659	Y	Y
PLCB1	Phospholipase C, beta 1 (phosphoinositide-specific)	ChEST 740a8	Gga.38382	Y	
PLCB4	Phospholipase C, beta 4	ChEST 571b2	Gga.22043	Y	
PLCD1	Phospholipase C, delta 1	ChEST 600e16	Gga.3447	Y	

PLCH1	Phospholipase C, eta 1	ChEST 630h12	Gga.2646	Y	Y
PLCH2	Phospholipase C, eta 2	ChEST 711m24	Gga.19520		
PLCL1	Phospholipase C-like 1	ChEST 703i1	Gga.22031	Y	
PLCL2	Phospholipase C-like 2	ChEST 698g9	Gga.25229		
PLCXD1	Phosphatidylinositol-specific phospholipase C, X domain containing 1	ChEST 691g14	Gga.24899	Y	
PLD4	Phospholipase D family, member 4	ChEST 631h8	Gga.22995	Y	
PLD6	Similar to novel Phospholipase D Active site motif-containing protein	ChEST 713e7	Gga.31077	Y	Y

PLK1	Polo-like kinase 1 (Drosophila)	ChEST 659i4	Gga.22245	Y	
PLK3	Polo-like kinase 3 (Drosophila)	ChEST 660j6	Gga.8732	Y	
PLOD1	Procollagen-lysine 1, 2-oxoglutarate 5-dioxygenase 1	ChEST 592o7	Gga.4726	Y	Y
PLSCR1	Phospholipid scramblase 1	ChEST 586a23	Gga.7604	Y	Y
PMM2	Phosphomannomutase 2	ChEST 514k1	Gga.13436	Y	Y
PMPCA	Transcribed locus, moderately similar to XP_001176403.1 PREDICTED: similar to fructose-biphosphate aldolase [Strongylocentrotus purpuratus]	ChEST 564d17	Gga.4745	Y	Y
PMS1	PMS1 postmeiotic segregation increased 1 (S. cerevisiae)	ChEST 647h15	Gga.4931	Y	

PNLIP	Pancreatic lipase	ChEST 615l15	Gga.4868	Y	
PNPLA7	Patatin-like phospholipase domain containing 7	ChEST 654h11	Gga.5922	Y	
PNPLA8	Patatin-like phospholipase domain containing 8	ChEST 622l1	Gga.35937		
PNPT1	Polyribonucleotide nucleotidyltransferase 1	ChEST 622j17	Gga.15745	Y	
POFUT1	Protein O-fucosyltransferase 1	ChEST 387m19	Gga.5100	Y	
POFUT2	Protein O-fucosyltransferase 2	ChEST 613e20	Gga.8806	Y	
POMP	Proteasome maturation protein	ChEST 634j7	Gga.5765	Y	

POMT1	Protein-O-mannosyltransferase 1	ChEST 718c21	Gga.22554	Y	
POMT2	Protein-O-mannosyltransferase 2	ChEST 602d9	Gga.43262	Y	
PON2	Paraoxonase 2	ChEST 646g1	Gga.23753	Y	
POP5	Processing of precursor 5, ribonuclease P/MRP subunit (S. cerevisiae)	ChEST 605p23	Gga.39315		
POR	P450 (cytochrome) oxidoreductase	ChEST 603j3	Gga.8335	Y	Y
PPA1	Pyrophosphatase (inorganic) 1	ChEST 649h24	Gga.4740	Y	Y
PPAP2B	Phosphatidic acid phosphatase type 2B	ChEST 653l11	Gga.1568	Y	

PPAPDC1B	Phosphatidic acid phosphatase type 2 domain containing 1B	ChEST 631h21	Gga.35295	Y	
PPAT	Phosphoribosyl pyrophosphate amidotransferase	ChEST 697o5	Gga.2870	Y	
PPCDC	Phosphopantothenoylcysteine decarboxylase	ChEST 597i22	Gga.10779		
PPEF1	Similar to serine /threonine protein phosphatase	ChEST 670k5	Gga.30225	Y	Y
PPFIA1	Protein tyrosine phosphatase, receptor type, f polypeptide (PTPRF), interacting protein (liprin), alpha 1	ChEST 685n6	Gga.12241	Y	
PPFIA2	Protein tyrosine phosphatase, receptor type, f polypeptide (PTPRF), interacting protein (liprin), alpha 2	ChEST 669a21	Gga.10621		
PPIB	Peptidylprolyl isomerase B (cyclophilin B)	ChEST 667k17	Gga.3382	Y	Y

PPID	Peptidylprolyl isomerase D (cyclophilin D)	ChEST 374e8	Gga.2412	Y	Y
PPIE	Peptidylprolyl isomerase E (cyclophilin E)	ChEST 533n23	Gga.4709		
PPIH	Peptidylprolyl isomerase H (cyclophilin H)	ChEST 651l2	Gga.7074	Y	
PPIL2	Peptidylprolyl isomerase (cyclophilin)-like 2	ChEST 655j9	Gga.17248	Y	
PPIL3	Peptidylprolyl isomerase (cyclophilin)-like 3	ChEST 709g4	Gga.6287		
PPIL4	Peptidylprolyl isomerase (cyclophilin)-like 4	ChEST 691c11	Gga.34615	Y	
PPM1A	Protein phosphatase 1A (formerly 2C), magnesium-dependent, alpha isoform	ChEST 604h8	Gga.28906	Y	Y

PPM1B	Protein phosphatase 1B (formerly 2C), magnesium-dependent, beta isoform	ChEST 604g14	Gga.43302		
PPM1D	Protein phosphatase 1D magnesium-dependent, delta isoform	ChEST 578d22	Gga.2181		
PPM1E	Protein phosphatase 1E (PP2C domain containing)	ChEST 533d9	Gga.42973	Y	Y
PPM1F	Protein phosphatase 1F (PP2C domain containing)	ChEST 533a4	Gga.40581	Y	
PPM1J	Protein phosphatase 1J (PP2C domain containing)	ChEST 571h13	Gga.32455	Y	
PPM1K	Protein phosphatase 1K (PP2C domain containing)	ChEST 663f17	Gga.41931		
PPM1M	Protein phosphatase 1M (PP2C domain containing)	ChEST 634o10	Gga.4799	Y	

PPP1CB	Protein phosphatase 1, catalytic subunit, beta isoform	ChEST 592e10	Gga.42422		
PPP1CC	Protein phosphatase 1, catalytic subunit, gamma isoform	ChEST 620b10	Gga.5664	Y	
PPP1R10	Protein phosphatase 1, regulatory (inhibitor) subunit 10	ChEST 520m19	Gga.15195	Y	
PPP1R12B	Protein phosphatase 1M regulatory subunit	ChEST 668h21	Gga.2172	Y	Y
PPP1R13B	Protein phosphatase 1, regulatory (inhibitor) subunit 13B	ChEST 635o6	Gga.7098	Y	
PPP1R14C	Protein phosphatase 1, regulatory (inhibitor) subunit 14C	ChEST 533m11	Gga.15918	Y	Y
PPP1R14D	Protein phosphatase 1, regulatory (inhibitor) subunit 14D	ChEST 602a17	Gga.7422	Y	

PPP1R1C	Protein phosphatase 1, regulatory (inhibitor) subunit 1C	ChEST 675f11	Gga.11862	Y	
PPP1R2	Protein phosphatase 1, regulatory (inhibitor) subunit 2	ChEST 638d3	Gga.22942	Y	Y
PPP1R7	Protein phosphatase 1, regulatory (inhibitor) subunit 7	ChEST 660m10	Gga.5583	Y	
PPP1R8	Protein phosphatase 1, regulatory (inhibitor) subunit 8	ChEST 630n2	Gga.3224	Y	Y
PPP2CB	Protein phosphatase 2 (formerly 2A), catalytic subunit, beta isoform	ChEST 604j4	Gga.4287	Y	
PPP2R2A	Protein phosphatase 2 (formerly 2A), regulatory subunit B, alpha isoform	ChEST 597f7	Gga.9510	Y	Y
PPP2R2C	Protein phosphatase 2 (formerly 2A), regulatory subunit B, gamma isoform	ChEST 710b15	Gga.12447	Y	Y

PPP2R2D	Protein phosphatase 2, regulatory subunit B, delta isoform	ChEST 600c24	Gga.1129		
PPP2R3A	Protein phosphatase 2 (formerly 2A), regulatory subunit B", alpha	ChEST 634c24	Gga.16207	Y	
PPP2R3B	Protein phosphatase 2 (formerly 2A), regulatory subunit B", beta	ChEST 658o23	Gga.12463		
PPP2R4	Protein phosphatase 2A activator, regulatory subunit 4	ChEST 586n2	Gga.999	Y	Y
PPP2R4	Protein phosphatase 2A activator, regulatory subunit 4	ChEST 586n2	Gga.999		
PPP2R5A	Protein phosphatase 2, regulatory subunit B', alpha isoform	ChEST 563m17	Gga.39550	Y	
PPP2R5C	Protein phosphatase 2, regulatory subunit B', gamma isoform	ChEST 627p18	Gga.17571	Y	

PPP2R5E	Protein phosphatase 2, regulatory subunit B', epsilon isoform	ChEST 685o3	Gga.36779	Y	
PPP3CA	Protein phosphatase 3 (formerly 2B), catalytic subunit, alpha isoform	ChEST 604a18	Gga.3869		
PPP3R1	Protein phosphatase 3 (formerly 2B), regulatory subunit B, alpha isoform	ChEST 645n17	Gga.3833		
PPP4R2	Protein phosphatase 4, regulatory subunit 2	ChEST 514c12	Gga.5231	Y	
PPT1	Palmitoyl-protein thioesterase 1 (ceroid-lipofuscinosis, neuronal 1, infantile)	ChEST 597n18	Gga.4593	Y	
PPTC7	PTC7 protein phosphatase homolog (S. cerevisiae)	ChEST 640f19	Gga.12843	Y	
PPWD1	Peptidylprolyl isomerase domain and WD repeat containing 1	ChEST 374n7	Gga.23666	Y	

PRCP	Prolylcarboxypeptidase (angiotensinase C)	ChEST 586k13	Gga.34843	Y	Y
PRDX1	Peroxiredoxin 1	ChEST 387h2	Gga.5204	Y	
PRDX3	Peroxiredoxin 3	ChEST 689j24	Gga.8044	Y	
PRDX4	Peroxiredoxin 4	ChEST 609e6	Gga.34481	Y	
PRDX6	Peroxiredoxin 6	ChEST 608h9	Gga.34325		
PREP	Prolyl endopeptidase	ChEST 571g7	Gga.22224	Y	
PREPL	Prolyl endopeptidase-like	ChEST 747n20	Gga.40410	Y	Y

PRIM1	Primase, polypeptide 1, 49kDa	ChEST 641e24	Gga.22829	Y	
PRIM2	Primase, DNA, polypeptide 2 (58kDa)	ChEST 658j7	Gga.2485	Y	
PRKAA1	Protein kinase, AMP-activated, alpha 1 catalytic subunit	ChEST 624c14	Gga.33723	Y	Y
PRKAA2	Protein kinase, AMP-activated, alpha 2 catalytic subunit	ChEST 666j7	Gga.33715	Y	
PRKAB1	Protein kinase, AMP-activated, beta 1 non-catalytic subunit	ChEST 620c18	Gga.7310	Y	Y
PRKAG1	Transcribed locus, strongly similar to NP_003782.1 membrane-bound transcription factor site-1 protease preproprotein [Homo sapiens]	ChEST 387j14	Gga.29062	Y	
PRKAR1B	Protein kinase, cAMP-dependent, regulatory, type I, beta	ChEST 661j20	Gga.9736	Y	Y

PRKAR2A	Protein kinase, cAMP-dependent, regulatory, type II, alpha	ChEST 605a16	Gga.37800	Y	
PRKCB1	Protein kinase C, beta 1	ChEST 603c4	Gga.7157	Y	
PRKCD	Protein kinase C, delta	ChEST 619k3	Gga.21292	Y	
PRKCE	Protein kinase C, epsilon	ChEST 637f23	Gga.40318	Y	
PRKCH	Protein kinase C, eta	ChEST 655b12	Gga.8329	Y	Y
PRKDC	Protein kinase, DNA-activated, catalytic polypeptide	ChEST 603p3	Gga.189		
PRKRIR	Protein-kinase, interferon-inducible double stranded RNA dependent inhibitor, repressor of (P58 repressor)	ChEST 739p6	Gga.20433	Y	

PRMT3	Protein arginine methyltransferase 3	ChEST 637o6	Gga.39694	Y	
PRMT7	Protein arginine methyltransferase 7	ChEST 659b16	Gga.22346		
PRMT8	Protein arginine methyltransferase 8	ChEST 630h13	Gga.39488		
PRNP	Prion protein (p27-30) (Creutzfeldt-Jakob disease, Gerstmann-Strausler-Scheinker syndrome, fatal familial insomnia)	ChEST 630n21	Gga.3867	Y	Y
PROSC	Proline synthetase co-transcribed homolog (bacterial)	ChEST 624d17	Gga.1013	Y	Y
PRPS1	Phosphoribosyl pyrophosphate synthetase 1	ChEST 667p9	Gga.19287	Y	Y
PRPS2	Phosphoribosyl pyrophosphate synthetase 2	ChEST 727a23	Gga.7766	Y	

PRPSAP1	Phosphoribosyl pyrophosphate synthetase-associated protein 1	ChEST 605n2	Gga.22539	Y	
PRSS2	Protease, serine, 2 (trypsin 2)	ChEST 615a17	Gga.4279		
PRSS23	Protease, serine, 23	ChEST 613m11	Gga.5640		
PRSS3	Protease, serine, 3	ChEST 615b10	Gga.4442		
PRSS35	Protease, serine, 35 (PRSS35)	ChEST 586m21	Gga.26708	Y	
PRSSL1	Protease, serine-like 1	ChEST 708i12	Gga.24757	Y	
PSAT1	Phosphoserine aminotransferase 1	ChEST 647g19	Gga.1510	Y	

PSEN1	Presenilin 1 (Alzheimer disease 3)	ChEST 533l9	Gga.3864	Y	
PSMA1	Proteasome (prosome, macropain) subunit, alpha type, 1	ChEST 606a16	Gga.3661		
PSMA2	Proteasome (prosome, macropain) subunit, alpha type, 2	ChEST 644l6	Gga.23720	Y	Y
PSMA7	Proteasome (prosome, macropain) subunit, alpha type, 7	ChEST 648i19	Gga.2045	Y	
PSMB2	Proteasome (prosome, macropain) subunit, beta type, 2	ChEST 646a20	Gga.8492	Y	
PSMB3	Proteasome (prosome, macropain) subunit, beta type, 3	ChEST 646p11	Gga.1459		
PSMB4	Proteasome (prosome, macropain) subunit, beta type, 4	ChEST 646j5	Gga.1184		

PSMD10	Proteasome (prosome, macropain) 26S subunit, non-ATPase, 10	ChEST 628h8	Gga.12265		
PSMD12	Proteasome (prosome, macropain) 26S subunit, non-ATPase, 12	ChEST 601b7	Gga.21287	Y	Y
PSMD14	Proteasome (prosome, macropain) 26S subunit, non-ATPase, 14	ChEST 735c15	Gga.22172	Y	Y
PSMD2	Proteasome (prosome, macropain) 26S subunit, non-ATPase, 2	ChEST 602h15	Gga.1351		
PSMD5	Proteasome (prosome, macropain) 26S subunit, non-ATPase, 5	ChEST 611g24	Gga.42443		
PSMD6	Proteasome (prosome, macropain) 26S subunit, non-ATPase, 6	ChEST 597o13	Gga.39564		
PSMD7	Proteasome (prosome, macropain) 26S subunit, non-ATPase, 7 (Mov34 homolog)	ChEST 713m20	Gga.9789	Y	

PSME3	Proteasome (prosome, macropain) activator subunit 3 (PA28 gamma; Ki)	ChEST 620o5	Gga.5999	Y	
PSMF1	Proteasome (prosome, macropain) inhibitor subunit 1 (PI31)	ChEST 662g10	Gga.39344	Y	
PSMG3	Proteasome (prosome, macropain) assembly chaperone 3	ChEST 647f15	Gga.34281		
PSPH	Phosphoserine phosphatase	ChEST 621f7	Gga.4663		
PSTK	Phosphoseryl-tRNA kinase	ChEST 665k18	Gga.35681		
PSTPIP2	Proline-serine-threonine phosphatase interacting protein 2	ChEST 668i18	Gga.36176	Y	
PTDSS1	Phosphatidylserine synthase 1	ChEST 718p19	Gga.16724	Y	

PTEN	Phosphatase and tensin homolog (mutated in multiple advanced cancers 1)	ChEST 622j21	Gga.13561		
PTER	Phosphotriesterase related	ChEST 639m7	Gga.13173	Y	
PTGDS	Prostaglandin D2 synthase 21kDa (brain)	ChEST 609l8	Gga.3350		
PTGES	Prostaglandin E synthase	ChEST 606n15	Gga.12397	Y	Y
PTGES2	Prostaglandin E synthase 2	ChEST 592c14	Gga.2926	Y	Y
PTK2	Transcribed locus, moderately similar to NP_058043.3 hydroxyacyl-Coenzyme A dehydrogenase type II [Mus musculus]	ChEST 722m13	Gga.42877	Y	Y
PTK2	PTK2 protein tyrosine kinase 2	ChEST 617f2	Gga.42870		

PTK7	PTK7 protein tyrosine kinase 7	ChEST 643g21	Gga.43472	Y	Y
PTP4A1	Protein tyrosine phosphatase type IVA, member 1	ChEST 600e8	Gga.4888		
PTP4A2	Protein tyrosine phosphatase type IVA, member 2	ChEST 658i4	Gga.5381	Y	
PTP4A3	Protein tyrosine phosphatase type IVA, member 3	ChEST 670m1	Gga.39491		
PTPDC1	Protein tyrosine phosphatase domain containing 1	ChEST 604n5	Gga.29510	Y	
PTPLA	Protein tyrosine phosphatase-like (proline instead of catalytic arginine), member A	ChEST 651l7	Gga.2576	Y	
PTPLAD1	Protein tyrosine phosphatase-like A domain containing 1	ChEST 639n5	Gga.1825	Y	

PTPLB	Protein tyrosine phosphatase-like (proline instead of catalytic arginine), member b	ChEST 597b15	Gga.20415		
PTPN11	Protein tyrosine phosphatase, non-receptor type 11	ChEST 620n17	Gga.482		
PTPN12	Protein tyrosine phosphatase, non-receptor type 12	ChEST 600b16	Gga.28765	Y	
PTPN13	Similar to protein tyrosine phosphatase type 1	ChEST 645l12	Gga.12818	Y	Y
PTPN14	Protein tyrosine phosphatase, non-receptor type 14	ChEST 664o20	Gga.25314	Y	
PTPN2	Protein tyrosine phosphatase, non-receptor type 2	ChEST 564b8	Gga.42075	Y	Y
PTPN21	Protein tyrosine phosphatase, non-receptor type 21	ChEST 824n10	Gga.25199	Y	

PTPN3	Protein tyrosine phosphatase, non-receptor type 3	ChEST 705d11	Gga.13704		
PTPN4	Transcribed locus, weakly similar to NP_000148.2 glucocerebrosidase precursor [Homo sapiens]	ChEST 675l21	Gga.30498	Y	
PTPN5	Protein tyrosine phosphatase, non-receptor type 5 (striatum-enriched)	ChEST 678j24	Gga.10177	Y	
PTPN6	Protein tyrosine phosphatase, non-receptor type 6	ChEST 619b19	Gga.9379	Y	
PTPN9	Protein tyrosine phosphatase, non-receptor type 9	ChEST 665g16	Gga.28681	Y	Y
PTPRA	Protein tyrosine phosphatase, receptor type, A	ChEST 563h5	Gga.11228	Y	
PTPRB	Protein tyrosine phosphatase, receptor type, B	ChEST 704j16	Gga.24653		

PTPRC	Protein tyrosine phosphatase, receptor type, C	ChEST 619l4	Gga.1193	Y	
PTPRD	Similar to protein tyrosine phosphatase, receptor type, D	ChEST 571j2	Gga.32428	Y	Y
PTPRF	Protein tyrosine phosphatase, receptor type, F	ChEST 602d20	Gga.12338	Y	
PTPRG	Protein tyrosine phosphatase, receptor type, G	ChEST 514c11	Gga.2516	Y	
PTPRJ	Protein tyrosine phosphatase, receptor type, J	ChEST 691l12	Gga.966	Y	
PTPRN2	Protein tyrosine phosphatase, receptor type, N polypeptide 2	ChEST 661d22	Gga.28277	Y	
PTPRR	Protein tyrosine phosphatase, receptor type, R	ChEST 533g4	Gga.26691	Y	Y

PTPRS	Protein tyrosine phosphatase, receptor type, S	ChEST 650h19	Gga.42907	Y	Y
PTPRU	Transcribed locus, strongly similar to NP_001012550.1 euchromatic histone-lysine N-methyltransferase 1 [Gallus gallus]	ChEST 653j10	Gga.43629	Y	
PTRH2	Peptidyl-tRNA hydrolase 2	ChEST 597g15	Gga.1086		
PTS	6-pyruvoyltetrahydropterin synthase	ChEST 684e1	Gga.5120	Y	
PUS1	Pseudouridylate synthase 1	ChEST 533f5	Gga.16736	Y	
PUS7	Pseudouridylate synthase 7 homolog (S. cerevisiae)	ChEST 709k5	Gga.8127		
PUS7L	Pseudouridylate synthase 7 homolog (S. cerevisiae)-like	ChEST 600b10	Gga.2946		

PUSL1	Pseudouridylate synthase-like 1	ChEST 609f22	Gga.10892	Y	Y
PXK	PX domain containing serine/threonine kinase	ChEST 660b16	Gga.39298	Y	Y
PYCR2	Pyrroline-5-carboxylate reductase family, member 2	ChEST 374m13	Gga.9281	Y	
PYGL	Liver glycogen phosphorylase	ChEST 631f17	Gga.7031	Y	
QDPR	Quinoid dihydropteridine reductase	ChEST 649n21	Gga.21121	Y	Y
QPCT	Glutaminyl-peptide cyclotransferase (glutaminyl cyclase)	ChEST 718d10	Gga.12082	Y	Y
QRSL1	Glutaminyl-tRNA synthase (glutamine-hydrolyzing)-like 1	ChEST 620l13	Gga.11968		

QSOX2	Quiescin Q6 sulfhydryl oxidase 2	ChEST 374c1	Gga.18094	Y	
QTRTD1	Queuine tRNA-ribosyltransferase domain containing 1	ChEST 597p14	Gga.4463	Y	Y
RAB3GAP1	RAB3 GTPase activating protein subunit 1 (catalytic)	ChEST 593f23	Gga.30904	Y	
RAB3GAP2	RAB3 GTPase activating protein subunit 2 (non-catalytic)	ChEST 656d23	Gga.10761	Y	
RABEP1	Rabaptin, RAB GTPase binding effector protein 1	ChEST 520c8	Gga.549	Y	Y
RABGAP1	RAB GTPase activating protein 1	ChEST 624a21	Gga.15938		
RABGGTB	Rab geranylgeranyltransferase, beta subunit	ChEST 620c24	Gga.12066	Y	

RANGAP1	Ran GTPase activating protein 1	ChEST 638e9	Gga.6270	Y	Y
RARS	Arginyl-tRNA synthetase	ChEST 602c14	Gga.22550	Y	
RASA1	RAS p21 protein activator (GTPase activating protein) 1	ChEST 629g8	Gga.14265	Y	
RBL2	Transcribed locus, weakly similar to XP_862635.1 PREDICTED: similar to sphingomyelin phosphodiesterase 1, acid lysosomal isoform 1 precursor isoform 2 [Canis familiaris]	ChEST 605n4	Gga.43166		
RBM43	Transcribed locus, weakly similar to NP_001009670.1 abhydrolase domain containing 14A [Rattus norvegicus]	ChEST 638e7	Gga.31471	Y	Y
RCJMB04_10a6	FAD-dependent oxidoreductase domain containing 2	ChEST 678d11	Gga.22308	Y	
RCJMB04_10m2	Histone deacetylase 9	ChEST 387d7	Gga.1378		

RCJMB04_11e1	Endoplasmic reticulum aminopeptidase 1	ChEST 631f4	Gga.1140	Y	
RCJMB04_11l2	Tyrosine 3-monooxygenase/tryptophan 5-monooxygenase activation protein, zeta polypeptide	ChEST 609a9	Gga.8276		
RCJMB04_11p1	Asparagine-linked glycosylation 10 homolog (yeast, alpha-1,2-glucosyltransferase)	ChEST 601o17	Gga.12946		
RCJMB04_12b8	Methylenetetrahydrofolate dehydrogenase (NADP+ dependent) 2, methenyltetrahydrofolate cyclohydrolase	ChEST 564j23	Gga.2672		
RCJMB04_12m	Acyl-Coenzyme A dehydrogenase, short/branched chain	ChEST 593k14	Gga.19133	Y	
RCJMB04_13a2	Signal peptide peptidase-like 2A	ChEST 619j19	Gga.9215	Y	
RCJMB04_13m17	MYST histone acetyltransferase 2	ChEST 571b3	Gga.7189	Y	

RCJMB04_14h2	Transcribed locus, strongly similar to NP_001026759.1 bromodomain containing 4 [Gallus gallus]	ChEST 520o1	Gga.22362	Y	Y
RCJMB04_16e1	Ectonucleoside triphosphate diphosphohydrolase 1	ChEST 651l3	Gga.1884	Y	
RCJMB04_17g4	Pyruvate dehydrogenase complEX, component X	ChEST 745j2	Gga.20259	Y	
RCJMB04_17j1[illegible]	ADAM metallopeptidase domain 28	ChEST 603l9	Gga.19451	Y	
RCJMB04_17o1	Inhibitor of kappa light polypeptide gene enhancer in B-cells, kinase complEX-associated protein	ChEST 674d13	Gga.5317	Y	
RCJMB04_18p7	GTPase activating Rap/RanGAP domain-like 4	ChEST 622e16	Gga.22399	Y	
RCJMB04_19h2	Conserved helix-loop-helix ubiquitous kinase	ChEST 604c15	Gga.5483		

RCJMB04_19j12	Oxoglutarate (alpha-ketoglutarate) dehydrogenase (lipoamide)	ChEST 625g9	Gga.7238	Y	
RCJMB04_19j20	Guanosine monophosphate reductase	ChEST 631j12	Gga.45154		
RCJMB04_1f1	Solute carrier family 20 (phosphate transporter), member 2	ChEST 520o5	Gga.3329	Y	
RCJMB04_1f4	Similar to methylcrotonoyl-Coenzyme A carboxylase 2 (beta); non-biotin containing subunit of 3-methylcrotonyl-CoA carboxylase; biotin carboxylase	ChEST 684l17	Gga.22435	Y	Y
RCJMB04_1j11	IMP (inosine monophosphate) dehydrogenase 2	ChEST 621g16	Gga.5296	Y	
RCJMB04_1j22	Catalase	ChEST 611e9	Gga.1183		
RCJMB04_1o7	Peptidylprolyl isomerase F (cyclophilin F)	ChEST 658l11	Gga.1727	Y	

RCJMB04_1p22	Transcribed locus, strongly similar to XP_420139.2 PREDICTED: similar to aminopeptidase P [Gallus gallus]	ChEST 727a13	Gga.30057	Y	
RCJMB04_20c6	CDC-like kinase 2	ChEST 712j12	Gga.15733	Y	
RCJMB04_20j15	Phosphatidylinositol-4-phosphate 5-kinase, type I, beta	ChEST 622i11	Gga.17037		
RCJMB04_20o1	Meningioma EXpressed antigen 5 (hyaluronidase)	ChEST 630f13	Gga.16851	Y	
RCJMB04_21j5	Argininosuccinate synthetase 1	ChEST 678o4	Gga.5385	Y	
RCJMB04_21k9	RAB GTPase activating protein 1-like	ChEST 674h14	Gga.42152	Y	
RCJMB04_21o2	Similar to Serine/threonine kinase 3 (STE20 homolog, yeast)	ChEST 661m6	Gga.12683	Y	

RCJMB04_22k1	Protein phosphatase 1, regulatory (inhibitor) subunit 12A	ChEST 600d9	Gga.3159	Y	
RCJMB04_22m2	Solute carrier family 19, member 3	ChEST 619k20	Gga.32108	Y	
RCJMB04_23a5	Thioredoxin reductase 1	ChEST 675n5	Gga.4380	Y	
RCJMB04_23h2	Protein phosphatase 6, catalytic subunit	ChEST 630k21	Gga.2782	Y	
RCJMB04_24e1	Enolase 1, (alpha)	ChEST 664f21	Gga.1383	Y	
RCJMB04_27l14	Similar to UDP-glucose:glycoprotein glucosyltransferase 2	ChEST 571c8	Gga.22677		
RCJMB04_27p1	5-methyltetrahydrofolate-homocysteine methyltransferase	ChEST 685g1	Gga.21288	Y	

RCJMB04_28b1	WW domain containing oxidoreductase	ChEST 635d3	Gga.22968		
RCJMB04_28i1[illegible]	Acid phosphatase 2, lysosomal	ChEST 661n7	Gga.22582	Y	
RCJMB04_28l2[illegible]	Glucuronidase, beta	ChEST 661d20	Gga.43690	Y	
RCJMB04_2f16	Phosphorylase, glycogen; brain	ChEST 622a22	Gga.8203	Y	
RCJMB04_2g8	Serine/threonine kinase 11 interacting protein	ChEST 640n15	Gga.10922		
RCJMB04_2h11	Transcribed locus, strongly similar to XP_001234488.1 PREDICTED: similar to lysosomal alpha-N-acetyl glucosaminidase, partial [Gallus gallus]	ChEST 685k10	Gga.4672	Y	
RCJMB04_2k24	Similar to serine/threonine protein kinase MASK; STE20-like kinase MST4	ChEST 609f24	Gga.4492	Y	

RCJMB04_30g1	HEXosaminidase A (alpha polypeptide)	ChEST 592k19	Gga.8411	Y	
RCJMB04_31g1	Adenosine deaminase, tRNA-specific 1	ChEST 514m11	Gga.39178	Y	
RCJMB04_32e4	Solute carrier family 41, member 2	ChEST 649f3	Gga.21992	Y	
RCJMB04_33d2	Phosphofructokinase, platelet	ChEST 603a13	Gga.5753	Y	
RCJMB04_33e1	Phosphoglucomutase 2	ChEST 651i12	Gga.21172	Y	
RCJMB04_34p8	Transcribed locus, moderately similar to XP_001235180.1 PREDICTED: similar to Cytochrome P450 4A2 precursor (CYPIVA2) (Lauric acid omega-hydroxylase) (P450-LA-omega 2) (P450	ChEST 722j5	Gga.43183		
RCJMB04_35i2[illegible]	Protein phosphatase methylesterase 1	ChEST 689b4	Gga.16890	Y	

RCJMB04_36h7	Mitogen-activated protein kinase kinase 5	ChEST 667d11	Gga.42556	Y	
RCJMB04_39i8	Dihydrolipoamide S-succinyltransferase (E2 component of 2-oxo-glutarate complEX)	ChEST 593d10	Gga.6085	Y	
RCJMB04_3e13	PAN2 polyA specific ribonuclease subunit homolog (S. cerevisiae)	ChEST 654g5	Gga.18769	Y	
RCJMB04_3n15	Stromal membrane-associated GTPase-activating protein 2	ChEST 374c16	Gga.4890	Y	Y
RCJMB04_4c5	CTP synthase II	ChEST 631d16	Gga.16217	Y	
RCJMB04_4j19	Acetyl-Coenzyme A acyltransferase 1 (peroxisomal 3-oxoacyl-Coenzyme A thiolase)	ChEST 706i4	Gga.17023		
RCJMB04_4k14	Threonyl-tRNA synthetase	ChEST 725f10	Gga.22081		

RCJMB04_4l2	G-2 and S-phase EXpressed 1	ChEST 711m18	Gga.22410	Y	
RCJMB04_4o20	Pyruvate dehydrogenase kinase, isozyme 1	ChEST 602g17	Gga.21396		
RCJMB04_4p8	Similar to cytochrome b-5 reductase	ChEST 653o17	Gga.22504		
RCJMB04_5a10	Protein kinase C, alpha	ChEST 629l8	Gga.39759	Y	
RCJMB04_5e12	Tyrosine 3-monooxygenase/tryptophan 5-monooxygenase activation protein, gamma polypeptide	ChEST 387m6	Gga.9354		
RCJMB04_5f7	Similar to Histone lysine N methyltransferase, H3 lysine-9 specific 2 (Histone H3-K9 methyltransferase 2) (H3-K9-HMTase 2) (Suppressor of variegation 3-9 homolog 2) (Su(var)3-9	ChEST 650g13	Gga.13450	Y	
RCJMB04_5i17	Mitogen-activated protein kinase 6	ChEST 637k12	Gga.3411	Y	

RCJMB04_5j9	1-acylglycerol-3-phosphate O-acyltransferase 9	ChEST 669d7	Gga.16404	Y	
RCJMB04_5k4	Selenoprotein I	ChEST 597b10	Gga.4604		
RCJMB04_6n19	N-acyl phosphatidylethanolamine phospholipase D	ChEST 601g3	Gga.8588		
RCJMB04_6p13	Nicotinamide phosphoribosyltransferase	ChEST 592j21	Gga.22203	Y	
RCJMB04_7i6	Alpha-methylacyl-CoA racemase	ChEST 644d24	Gga.22560	Y	
RCJMB04_8d17	Peptidase M20 domain containing 1	ChEST 514l14	Gga.5406	Y	
RCJMB04_8f4	Solute carrier family 6 (neurotransmitter transporter, glycine), member 9	ChEST 578a14	Gga.5405	Y	

RCJMB04_9i11	Acyl-CoA synthetase bubblegum family member 2	ChEST 600i7	Gga.22498	Y	
RCL1	RNA terminal phosphate cyclase-like 1	ChEST 642o14	Gga.9558	Y	Y
RCN2	Transcribed locus, moderately similar to NP_000283.1 phosphorylase kinase, alpha 2 (liver) [Homo sapiens]	ChEST 647o15	Gga.42479		
RDH12	Retinol dehydrogenase 12 (all-trans/9-cis/11-cis)	ChEST 697i10	Gga.2472		
RDH14	Retinol dehydrogenase 14 (all-trans/9-cis/11-cis)	ChEST 629p21	Gga.9612	Y	
RECQL	RecQ protein-like (DNA helicase Q1-like)	ChEST 671k5	Gga.115	Y	
RETSAT	Retinol saturase (all-trans-retinol 13,14-reductase)	ChEST 564n2	Gga.3030	Y	

REXO1	REX1, RNA EXonuclease 1 homolog (S. cerevisiae)	ChEST 721i24	Gga.5256	Y	Y
RFNG	RFNG O-fucosylpeptide 3-beta-N-acetylglucosaminyltransferase	ChEST 647n17	Gga.4783		
RFX2	Transcribed locus, strongly similar to NP_078881.3 Rho GTPase activating protein 10 [Homo sapiens]	ChEST 722m20	Gga.29942	Y	Y
RG9MTD1	RNA (guanine-9-) methyltransferase domain containing 1	ChEST 739e6	Gga.5727	Y	
RHPN2	Rhophilin, Rho GTPase binding protein 2	ChEST 634c6	Gga.12909		
RIC8B	Resistance to inhibitors of cholinesterase 8 homolog B (C. elegans)	ChEST 729f20	Gga.7820		
RIOK1	RIO kinase 1 (yeast)	ChEST 627e7	Gga.9366	Y	

RIOK3	RIO kinase 3 (yeast)	ChEST 603o2	Gga.34487		
RIPK1	Receptor (TNFRSF)-interacting serine-threonine kinase 1	ChEST 673o17	Gga.5199		
RIPK2	Receptor-interacting serine-threonine kinase 2	ChEST 676h4	Gga.22379	Y	
RIPK4	Receptor-interacting serine-threonine kinase 4	ChEST 631d22	Gga.15973	Y	Y
RNASEH1	Ribonuclease H1	ChEST 651i4	Gga.500		
RNASEL	Ribonuclease L (2',5'-oligoisoadenylate synthetase-dependent)	ChEST 740j4	Gga.22526	Y	
RNASET2	Ribonuclease T2	ChEST 600h11	Gga.16342	Y	

RND3	Rho family GTPase 3	ChEST 638j17	Gga.35033	Y	Y
RNF41	ChDSD mRNA for chicken D-serine dehydratase	ChEST 799n21	Gga.11267		
RNGTT	RNA guanylyltransferase and 5'-phosphatase	ChEST 661k23	Gga.43858	Y	
RNMTL1	RNA methyltransferase like 1	ChEST 675j7	Gga.8859		
RNPEP	Arginyl aminopeptidase (aminopeptidase B)	ChEST 593b4	Gga.34791	Y	
ROCK1	Rho-associated, coiled-coil containing protein kinase 1	ChEST 681n21	Gga.34433	Y	
ROR1	Receptor tyrosine kinase-like orphan receptor 1	ChEST 667n11	Gga.9476		

RPE	Ribulose-5-phosphate-3-epimerase	ChEST 563c19	Gga.34478	Y	
RPIA	Ribose 5-phosphate isomerase A (ribose 5-phosphate epimerase)	ChEST 608o14	Gga.22302		
RPP14	Ribonuclease P 14kDa subunit	ChEST 597n1	Gga.4482		
RPP38	Ribonuclease P/MRP 38kDa subunit	ChEST 675c2	Gga.6844	Y	
RPP40	Ribonuclease P 40kDa subunit	ChEST 676e2	Gga.8882	Y	
RPS6KA1	Ribosomal protein S6 kinase, 90kDa, polypeptide 1	ChEST 654p21	Gga.4333	Y	
RPS6KA5	Ribosomal protein S6 kinase, 90kDa, polypeptide 5	ChEST 639i8	Gga.14856		

RPS6KA6	Ribosomal protein S6 kinase, 90kDa, polypeptide 6	ChEST 700p23	Gga.28146	Y	
RPS6KL1	Ribosomal protein S6 kinase-like 1	ChEST 662m11	Gga.13205	Y	
RPUSD4	RNA pseudouridylate synthase domain containing 4	ChEST 597b17	Gga.1463	Y	
RRM1	Ribonucleotide reductase M1 polypeptide	ChEST 650d2	Gga.4514	Y	
RRM2	Ribonucleotide reductase M2 polypeptide	ChEST 604a4	Gga.13504		
RRM2B	Ribonucleotide reductase M2 B (TP53 inducible)	ChEST 634g13	Gga.22087	Y	
RSC1A1	Regulatory solute carrier protein, family 1, member 1	ChEST 619a18	Gga.35503	Y	Y

RTCD1	RNA terminal phosphate cyclase domain 1	ChEST 564g17	Gga.9061		
RTEL1	Regulator of telomere elongation helicase 1	ChEST 617d22	Gga.24784	Y	
SAFB2	Transcribed locus, strongly similar to NP_001026223.1 protein phosphatase 1B [Gallus gallus]	ChEST 635a12	Gga.19313	Y	
SARS	Seryl-tRNA synthetase	ChEST 631b22	Gga.22320	Y	Y
SAT1	Spermidine/spermine N1-acetyltransferase 1	ChEST 592g3	Gga.9589	Y	
SC4MOL	Sterol-C4-methyl oxidase-like	ChEST 608g11	Gga.21297		
SCAMP2	Secretory carrier membrane protein 2	ChEST 597b13	Gga.13477	Y	

SCAMP5	Secretory carrier membrane protein 5	ChEST 722k4	Gga.39466		
SCAPER	S phase cyclin A-associated protein in the ER	ChEST 651f8	Gga.30035	Y	
SCCPDH	Saccharopine dehydrogenase (putative)	ChEST 613h21	Gga.4678		
SCD	Stearoyl-CoA desaturase (delta-9-desaturase)	ChEST 520e1	Gga.17055	Y	Y
SCD5	Stearoyl-CoA desaturase 5	ChEST 603f22	Gga.39443		
SCLY	Selenocysteine lyase	ChEST 593k13	Gga.7265	Y	
SCP2	Sterol carrier protein 2	ChEST 520a4	Gga.3425	Y	Y

SDHA	Succinate dehydrogenase complEX, subunit A, flavoprotein (Fp)	ChEST 586h1	Gga.993	Y	
SDHD	Succinate dehydrogenase complEX, subunit D, integral membrane protein	ChEST 571l14	Gga.34318		
SDSL	Serine dehydratase-like	ChEST 601o1	Gga.11448	Y	
SELS	Selenoprotein S	ChEST 651j24	Gga.3071	Y	
SENP1	SUMO1/sentrin specific peptidase 1	ChEST 738o18	Gga.21817	Y	Y
SENP2	SUMO1/sentrin/SMT3 specific peptidase 2	ChEST 617g7	Gga.19083	Y	Y
SENP5	SUMO1/sentrin specific peptidase 5	ChEST 627e15	Gga.39604	Y	

SENP6	SUMO1/sentrin specific peptidase 6	ChEST 698k22	Gga.22317	Y	
SENP8	SUMO/sentrin specific peptidase family member 8	ChEST 656e22	Gga.22747	Y	
SEPHS1	Selenophosphate synthetase 1	ChEST 387o13	Gga.5144	Y	
SEPP1	Selenoprotein P, plasma, 1	ChEST 606g11	Gga.34413	Y	Y
SEPSECS	Sep (O-phosphoserine) tRNA:Sec (selenocysteine) tRNA synthase	ChEST 727n7	Gga.22802	Y	
SEPX1	Selenoprotein X, 1	ChEST 631o15	Gga.12688	Y	
SERHL2	Serine hydrolase-like 2	ChEST 656g5	Gga.11488	Y	Y

SERPINA1	Serpin peptidase inhibitor, clade A (alpha-1 antiproteinase, antitrypsin), member 1	ChEST 731b12	Gga.10858	Y	
SERPINA10	Serpin peptidase inhibitor, clade A (alpha-1 antiproteinase, antitrypsin), member 10	ChEST 736m10	Gga.10644		
SERPINA5	Serpin peptidase inhibitor, clade A (alpha-1 antiproteinase, antitrypsin), member 5	ChEST 703h2	Gga.12540	Y	
SERPINB6	Serpin peptidase inhibitor, clade B (ovalbumin), member 6	ChEST 728p4	Gga.22267		
SERPINE2	Serpin peptidase inhibitor, clade E (nEXin, plasminogen activator inhibitor type 1), member 2	ChEST 674e16	Gga.4105	Y	
SERPINF2	Serpin peptidase inhibitor, clade F (alpha-2 antiplasmin, pigment epithelium derived factor), member 2	ChEST 612k13	Gga.1180	Y	
SERPINH1	Serpin peptidase inhibitor, clade H (heat shock protein 47), member 1, (collagen binding protein 1)	ChEST 586k11	Gga.4168	Y	Y

SERPINI1	Serpin peptidase inhibitor, clade I (neuroserpin), member 1	ChEST 717n11	Gga.4416		
SETD7	SET domain containing (lysine methyltransferase) 7	ChEST 630b2	Gga.20531	Y	Y
SGK269	NKF3 kinase family member	ChEST 610f17	Gga.1366		
SGK3	Serum/glucocorticoid regulated kinase family, member 3	ChEST 631c17	Gga.21399	Y	Y
SGMS1	Sphingomyelin synthase 1	ChEST 605f24	Gga.2939		
SGPL1	Sphingosine-1-phosphate lyase 1	ChEST 604m3	Gga.16071	Y	
SGSH	N-sulfoglucosamine sulfohydrolase (sulfamidase)	ChEST 681n24	Gga.10256	Y	

SH3KBP1	SH3-domain kinase binding protein 1	ChEST 606c2	Gga.3379		
SHMT1	Serine hydroxymethyltransferase 1 (soluble)	ChEST 624b12	Gga.12070	Y	
SKAP2	Src kinase associated phosphoprotein 2	ChEST 647i20	Gga.25340	Y	Y
SKP1	S-phase kinase-associated protein 1	ChEST 608a13	Gga.1718		
SKP2	S-phase kinase-associated protein 2 (p45)	ChEST 629a7	Gga.42319	Y	Y
SLC10A7	Solute carrier family 10 (sodium/bile acid cotransporter family), member 7	ChEST 697j17	Gga.19160	Y	
SLC12A7	Solute carrier family 12 (potassium/chloride transporters), member 7	ChEST 663l1	Gga.30049	Y	

SLC13A2	Solute carrier family 13 (sodium-dependent dicarboxylate transporter), member 2	ChEST 631p14	Gga.26124	Y	
SLC13A5	Solute carrier family 13 (sodium-dependent citrate transporter), member 5	ChEST 672p16	Gga.26164	Y	
SLC15A1	Solute carrier family 15 (oligopeptide transporter), member 1	ChEST 628e14	Gga.4106	Y	
SLC15A4	Solute carrier family 15, member 4	ChEST 621p20	Gga.39200		
SLC16A1	Solute carrier family 16, member 1 (monocarboxylic acid transporter 1)	ChEST 564c15	Gga.38993		
SLC16A10	Solute carrier family 16, member 10 (aromatic amino acid transporter)	ChEST 600l2	Gga.8200	Y	
SLC16A12	Solute carrier family 16, member 12 (monocarboxylic acid transporter 12)	ChEST 727g2	Gga.14029	Y	

SLC16A2	Solute carrier family 16, member 2 (monocarboxylic acid transporter 8)	ChEST 728h4	Gga.1750		
SLC16A2	Solute carrier family 16, member 2 (monocarboxylic acid transporter 8)	ChEST 728h4	Gga.1750		
SLC16A4	Solute carrier family 16, member 4 (monocarboxylic acid transporter 5)	ChEST 592l5	Gga.43169	Y	
SLC16A5	Solute carrier family 16, member 5 (monocarboxylic acid transporter 6)	ChEST 630p23	Gga.23796	Y	
SLC16A6	Solute carrier family 16, member 6 (monocarboxylic acid transporter 7)	ChEST 691g1	Gga.25054	Y	Y
SLC16A7	Solute carrier family 16, member 7 (monocarboxylic acid transporter 2)	ChEST 691f1	Gga.21283		
SLC17A5	Solute carrier family 17 (anion/sugar transporter), member 5	ChEST 639l3	Gga.22626	Y	Y

SLC18A3	Solute carrier family 18 (vesicular acetylcholine), member 3	ChEST 671a2	Gga.12077	Y	Y
SLC19A1	Solute carrier family 19 (folate transporter), member 1	ChEST 597c1	Gga.4821	Y	Y
SLC19A1	Solute carrier family 19 (folate transporter), member 1	ChEST 597c1	Gga.4821	Y	Y
SLC1A1	Solute carrier family 1 (neuronal/epithelial high affinity glutamate transporter, system Xag), member 1	ChEST 631j6	Gga.23808	Y	Y
SLC1A3	Solute carrier family 1 (glial high affinity glutamate transporter), member 3	ChEST 564p7	Gga.21206	Y	Y
SLC22A1	Solute carrier family 22 (organic cation transporter), member 1	ChEST 732g8	Gga.14910	Y	
SLC22A13	Solute carrier family 22, member 13	ChEST 592k8	Gga.38638	Y	

SLC22A18	Solute carrier family 22 (organic cation transporter), member 18	ChEST 651e15	Gga.6442		
SLC22A23	Solute carrier family 22, member 23	ChEST 619d12	Gga.20431	Y	
SLC22A3	Solute carrier family 22 (EXtraneuronal monoamine transporter), member 3	ChEST 619d3	Gga.40321	Y	Y
SLC23A2	Solute carrier family 23 (nucleobase transporters), member 2	ChEST 627k8	Gga.7071	Y	Y
SLC23A3	Solute carrier family 23 (nucleobase transporters), member 3	ChEST 586m10	Gga.15083	Y	
SLC24A5	Solute carrier family 24, member 5	ChEST 654m12	Gga.16998		
SLC24A5	Solute carrier family 24, member 5	ChEST 654m12	Gga.16998		

SLC24A6	Solute carrier family 24 (sodium/potassium/calcium EXchanger), member 6	ChEST 604h23	Gga.34523	Y	Y
SLC25A13	Solute carrier family 25, member 13 (citrin)	ChEST 602m3	Gga.4188		
SLC25A13	Solute carrier family 25, member 13 (citrin)	ChEST 602m3	Gga.4188		
SLC25A25	Similar to solute carrier family 25, member 25	ChEST 713p14	Gga.35614	Y	Y
SLC25A26	Solute carrier family 25, member 26	ChEST 630o19	Gga.11135	Y	
SLC25A28	Solute carrier family 25, member 28	ChEST 655a2	Gga.18831	Y	
SLC25A29	Solute carrier family 25, member 29	ChEST 747b4	Gga.8788		

SLC25A30	Solute carrier family 25, member 30	ChEST 739l19	Gga.29496	Y	
SLC25A32	Solute carrier family 25, member 32	ChEST 578j8	Gga.17073		
SLC25A33	Solute carrier family 25, member 33	ChEST 651j13	Gga.16161	Y	
SLC25A36	Solute carrier family 25, member 36	ChEST 617a13	Gga.15825	Y	
SLC25A43	Solute carrier family 25, member 43	ChEST 662o9	Gga.19825	Y	
SLC25A46	Solute carrier family 25, member 46	ChEST 620h23	Gga.44173	Y	
SLC26A11	Solute carrier family 26, member 11	ChEST 646m23	Gga.1730	Y	

SLC26A2	Solute carrier family 26 (sulfate transporter), member 2	ChEST 671h9	Gga.23654	Y	Y
SLC26A6	Solute carrier family 26, member 6	ChEST 619h11	Gga.40338	Y	Y
SLC26A8	Solute carrier family 26, member 8	ChEST 712k17	Gga.44779	Y	Y
SLC27A1	Solute carrier family 27 (fatty acid transporter), member 1	ChEST 668l24	Gga.33727	Y	
SLC27A4	Solute carrier family 27 (fatty acid transporter), member 4	ChEST 603l7	Gga.8457		
SLC27A6	Solute carrier family 27 (fatty acid transporter), member 6	ChEST 704c1	Gga.45792	Y	Y
SLC28A3	Solute carrier family 28 (sodium-coupled nucleoside transporter), member 3	ChEST 695c23	Gga.40913		

SLC29A1	Solute carrier family 29 (nucleoside transporters), member 1	ChEST 659g13	Gga.24319	Y	
SLC29A1	Transcribed locus, moderately similar to XP_420520.2 PREDICTED: similar to Polypeptide N-acetylgalactosaminyltransferase 17 [Gallus gallus]	ChEST 685c8	Gga.24323	Y	
SLC2A1	Solute carrier family 2 (facilitated glucose transporter), member 1	ChEST 592p7	Gga.1040	Y	
SLC2A10	Solute carrier family 2 (facilitated glucose transporter), member 10	ChEST 660e17	Gga.11730	Y	
SLC2A12	Solute carrier family 2 (facilitated glucose transporter), member 12	ChEST 706h4	Gga.7988		
SLC2A5	Solute carrier family 2 (facilitated glucose/fructose transporter), member 5	ChEST 617i17	Gga.29950	Y	Y
SLC2A6	Solute carrier family 2 (facilitated glucose transporter), member 6	ChEST 708o7	Gga.25593	Y	

SLC2A8	Solute carrier family 2, (facilitated glucose transporter) member 8	ChEST 533j9	Gga.43303	Y	Y
SLC30A1	Solute carrier family 30 (zinc transporter), member 1	ChEST 638a18	Gga.10012	Y	Y
SLC30A5	Solute carrier family 30 (zinc transporter), member 5	ChEST 605f20	Gga.42627		
SLC30A6	Solute carrier family 30 (zinc transporter), member 6	ChEST 571d1	Gga.17649	Y	
SLC30A9	Solute carrier family 30 (zinc transporter), member 9	ChEST 613p6	Gga.10399		
SLC31A1	Solute carrier family 31 (copper transporters), member 1	ChEST 635a21	Gga.2148	Y	
SLC31A2	Solute carrier family 31 (copper transporters), member 2	ChEST 630g15	Gga.7305	Y	Y

SLC31A2	Solute carrier family 31 (copper transporters), member 2	ChEST 630g15	Gga.7305		
SLC33A1	Solute carrier family 33 (acetyl-CoA transporter), member 1	ChEST 520m9	Gga.13557		
SLC34A2	Solute carrier family 34 (sodium phosphate), member 2	ChEST 630e21	Gga.216	Y	
SLC35A1	Solute carrier family 35 (CMP-sialic acid transporter), member A1	ChEST 628o11	Gga.4430	Y	
SLC35A3	Solute carrier family 35 (UDP-N-acetylglucosamine (UDP-GlcNAc) transporter), member A3	ChEST 630o7	Gga.22061	Y	
SLC35A5	Solute carrier family 35, member A5	ChEST 728g2	Gga.31406	Y	
SLC35B1	Solute carrier family 35, member B1	ChEST 597j21	Gga.1484	Y	

SLC35B2	Solute carrier family 35, member B2	ChEST 675c8	Gga.11551	Y	
SLC35B3	Solute carrier family 35, member B3	ChEST 609j10	Gga.32543	Y	
SLC35B4	Solute carrier family 35, member B4	ChEST 663f1	Gga.6228		
SLC35C1	Solute carrier family 35, member C1	ChEST 514g13	Gga.7831	Y	Y
SLC35C2	Solute carrier family 35, member C2	ChEST 597o12	Gga.22521	Y	
SLC35D1	Solute carrier family 35 (UDP-glucuronic acid/UDP-N-acetylgalactosamine dual transporter), member D1	ChEST 737h12	Gga.5479	Y	Y
SLC35E1	Solute carrier family 35, member E1	ChEST 628g9	Gga.9848	Y	

SLC35E2	Solute carrier family 35, member E2	ChEST 563a8	Gga.39384	Y	Y
SLC35E3	Solute carrier family 35, member E3	ChEST 533i16	Gga.5475	Y	
SLC35F1	Solute carrier family 35, member F1	ChEST 564b24	Gga.2764	Y	Y
SLC35F5	Solute carrier family 35, member F5	ChEST 621n23	Gga.34094	Y	Y
SLC36A1	Solute carrier family 36 (proton/amino acid symporter), member 1	ChEST 604d17	Gga.16922	Y	
SLC37A2	Solute carrier family 37 (glycerol-3-phosphate transporter), member 2	ChEST 609i24	Gga.2836	Y	
SLC37A3	Solute carrier family 37 (glycerol-3-phosphate transporter), member 3	ChEST 664g3	Gga.8894		

SLC37A4	Solute carrier family 37 (glucose-6-phosphate transporter), member 4	ChEST 638f19	Gga.39477	Y	Y
SLC38A2	Solute carrier family 38, member 2	ChEST 387m18	Gga.3105	Y	
SLC38A6	Solute carrier family 38, member 6	ChEST 641p9	Gga.26130	Y	Y
SLC38A7	Solute carrier family 38, member 7	ChEST 725m22	Gga.12758	Y	
SLC39A11	Solute carrier family 39 (metal ion transporter), member 11	ChEST 799g6	Gga.9966	Y	Y
SLC39A12	Solute carrier family 39 (zinc transporter), member 12	ChEST 719l14	Gga.13510		
SLC39A13	Solute carrier family 39 (zinc transporter), member 13	ChEST 520i9	Gga.42259	Y	Y

SLC39A6	Solute carrier family 39 (zinc transporter), member 6	ChEST 695l2	Gga.19094		
SLC39A9	Solute carrier family 39 (zinc transporter), member 9	ChEST 662a10	Gga.2489	Y	
SLC3A1	Solute carrier family 3 (cystine, dibasic and neutral amino acid transporters, activator of cystine, dibasic and neutral amino acid transport), member 1	ChEST 641l10	Gga.21925	Y	
SLC40A1	Solute carrier family 40 (iron-regulated transporter), member 1	ChEST 571a7	Gga.45184	Y	Y
SLC41A1	Solute carrier family 41, member 1	ChEST 563a21	Gga.14417	Y	Y
SLC43A2	Solute carrier family 43, member 2	ChEST 619c9	Gga.15714	Y	Y
SLC43A3	Solute carrier family 43, member 3	ChEST 640a16	Gga.5953	Y	

SLC44A5	Solute carrier family 44, member 5	ChEST 646f5	Gga.44610	Y	Y
SLC45A1	Solute carrier family 45, member 1	ChEST 640d14	Gga.12632	Y	
SLC46A1	Solute carrier family 46, member 1	ChEST 533j23	Gga.8993	Y	
SLC46A3	Solute carrier family 46, member 3	ChEST 617i19	Gga.8116	Y	Y
SLC4A1	Solute carrier family 4, anion EXchanger, member 1	ChEST 667o11	Gga.4842	Y	
SLC4A1AP	Solute carrier family 4 (anion EXchanger), member 1, adaptor protein	ChEST 603l15	Gga.16478	Y	
SLC4A4	Solute carrier family 4, sodium bicarbonate cotransporter, member 4	ChEST 592l2	Gga.28233	Y	Y

SLC4A7	Solute carrier family 4, sodium bicarbonate cotransporter, member 7	ChEST 642l21	Gga.9352	Y	Y
SLC5A1	Solute carrier family 5 (sodium/glucose cotransporter), member 1	ChEST 601p21	Gga.8594	Y	
SLC5A9	Solute carrier family 5 (sodium/glucose cotransporter), member 9	ChEST 603g17	Gga.10744	Y	
SLC6A11	Solute carrier family 6 (neurotransmitter transporter, GABA), member 11	ChEST 700e3	Gga.23546		
SLC6A13	Solute carrier family 6 (neurotransmitter transporter, GABA), member 13	ChEST 724o16	Gga.23026	Y	
SLC6A15	Solute carrier family 6, member 15	ChEST 732j3	Gga.29461		
SLC6A19	Solute carrier family 6 (neutral amino acid transporter), member 19	ChEST 624k8	Gga.30995	Y	

SLC6A4	Solute carrier family 6 (neurotransmitter transporter, serotonin), member 4	ChEST 601p17	Gga.10763	Y	
SLC6A5	Solute carrier family 6 (neurotransmitter transporter, glycine), member 5	ChEST 706i15	Gga.45780	Y	
SLC6A6	Solute carrier family 6 (neurotransmitter transporter, taurine), member 6	ChEST 597g4	Gga.43929	Y	Y
SLC7A1	Solute carrier family 7 (cationic amino acid transporter, y+ system), member 1	ChEST 701b2	Gga.29662	Y	
SLC7A10	Solute carrier family 7, (neutral amino acid transporter, y+ system) member 10	ChEST 725d2	Gga.39554	Y	Y
SLC7A3	Solute carrier family 7 (cationic amino acid transporter, y+ system), member 3	ChEST 637m19	Gga.36836	Y	
SLC7A4	Solute carrier family 7 (cationic amino acid transporter, y+ system), member 4	ChEST 668d12	Gga.15355	Y	

SLC7A5	Solute carrier family 7 (cationic amino acid transporter, y+ system), member 5	ChEST 564i14	Gga.22121	Y	Y
SLC7A6OS	Solute carrier family 7, member 6 opposite strand	ChEST 651n11	Gga.11874		
SLC7A9	Solute carrier family 7 (cationic amino acid transporter, y+ system), member 9	ChEST 625d4	Gga.8218	Y	
SLC9A1	Solute carrier family 9 (sodium/hydrogen EXchanger), member 1 (antiporter, Na+/H+, amiloride sensitive)	ChEST 609g14	Gga.33747	Y	Y
SLC9A3	Solute carrier family 9 (sodium/hydrogen EXchanger), member 3	ChEST 629b11	Gga.40337	Y	
SLC9A3R1	Solute carrier family 9 (sodium/hydrogen EXchanger), member 3 regulator 1	ChEST 649p10	Gga.1222	Y	
SLC9A3R2	Solute carrier family 9 (sodium/hydrogen EXchanger), member 3 regulator 2	ChEST 374b16	Gga.39551	Y	Y

SLC9A6	Solute carrier family 9 (sodium/hydrogen EXchanger), member 6	ChEST 667a11	Gga.17193	Y	
SLC9A7	Solute carrier family 9 (sodium/hydrogen EXchanger), member 7	ChEST 698i14	Gga.7330	Y	
SLC9A8	Solute carrier family 9 (sodium/hydrogen EXchanger), member 8	ChEST 602p19	Gga.13523	Y	
SLCO1B3	Solute carrier organic anion transporter family, member 1B3	ChEST 672h22	Gga.23751	Y	
SLCO2A1	Solute carrier organic anion transporter family, member 2A1	ChEST 722d12	Gga.8256	Y	
SLCO4A1	Solute carrier organic anion transporter family, member 4A1	ChEST 605d18	Gga.16658		
SLCO5A1	Solute carrier organic anion transporter family, member 5A1	ChEST 665k12	Gga.13871	Y	

SLK	STE20-like kinase (yeast)	ChEST 660p18	Gga.29709	Y	
SMAD3	Transcribed locus, weakly similar to XP_417972.2 PREDICTED: similar to SLIT-ROBO Rho GTPase activating protein 2 [Gallus gallus]	ChEST 664i19	Gga.44059	Y	
SMEK2	Transcribed locus, weakly similar to NP_853511.2 abhydrolase domain containing 12B isoform b [Homo sapiens]	ChEST 610c12	Gga.42157		
SMPD2	Sphingomyelin phosphodiesterase 2, neutral membrane (neutral sphingomyelinase)	ChEST 652i6	Gga.11867	Y	
SMPD3	Sphingomyelin phosphodiesterase 3, neutral membrane (neutral sphingomyelinase II)	ChEST 746o10	Gga.1652	Y	Y
SMPD4	Sphingomyelin phosphodiesterase 4, neutral membrane (neutral sphingomyelinase-3)	ChEST 664b3	Gga.16003	Y	
SMPDL3B	Sphingomyelin phosphodiesterase, acid-like 3B	ChEST 597o5	Gga.17301	Y	

SMS	Spermine synthase	ChEST 685l15	Gga.17551	Y	
SMURF1	SMAD specific E3 ubiquitin protein ligase 1	ChEST 563c9	Gga.20622	Y	
SMURF2	SMAD specific E3 ubiquitin protein ligase 2	ChEST 700c21	Gga.32493	Y	
SNF1LK	SNF1-like kinase	ChEST 978a23	Gga.2833	Y	Y
SNF1LK2	SNF1-like kinase 2	ChEST 696o8	Gga.39419		
SOAT1	Sterol O-acyltransferase (acyl-Coenzyme A: cholesterol acyltransferase) 1	ChEST 605p14	Gga.7080	Y	
SOD1	Superoxide dismutase 1, soluble (amyotrophic lateral sclerosis 1 (adult))	ChEST 604i6	Gga.3346	Y	

SOD3	Superoxide dismutase 3, EXtracellular	ChEST 617g8	Gga.1128	Y	Y
SORD	Sorbitol dehydrogenase	ChEST 631i2	Gga.9991	Y	Y
SPCS1	Signal peptidase complEX subunit 1 homolog (S. cerevisiae)	ChEST 374h9	Gga.16842	Y	
SPCS2	Signal peptidase complEX subunit 2 homolog (S. cerevisiae)	ChEST 625f13	Gga.8045	Y	
SPCS3	Signal peptidase complEX subunit 3 homolog (S. cerevisiae)	ChEST 608a17	Gga.3294	Y	
SPINK5	Serine peptidase inhibitor, Kazal type 5	ChEST 696p19	Gga.24701		
SPTLC3	Serine palmitoyltransferase, long chain base subunit 3	ChEST 738p11	Gga.45426	Y	

SQLE	Squalene epoxidase	ChEST 593n20	Gga.22304	Y	
SQRDL	Sulfide quinone reductase-like (yeast)	ChEST 713k14	Gga.22782	Y	Y
SRD5A2L2	Steroid 5 alpha-reductase 2-like 2	ChEST 664h24	Gga.12132	Y	Y
SRD5A3	Steroid 5 alpha-reductase 3	ChEST 563n13	Gga.39957	Y	
SRGAP1	SLIT-ROBO Rho GTPase activating protein 1	ChEST 563p22	Gga.12764		
SRGAP2	SLIT-ROBO Rho GTPase activating protein 2	ChEST 520k21	Gga.31979	Y	Y
SRM	Spermidine synthase	ChEST 654n5	Gga.4618		

SRPK1	SFRS protein kinase 1	ChEST 656o3	Gga.39181	Y	
ST3GAL1	ST3 beta-galactoside alpha-2,3-sialyltransferase 1	ChEST 660g17	Gga.3672	Y	
ST3GAL3	ST3 beta-galactoside alpha-2,3-sialyltransferase 3	ChEST 689e11	Gga.17429		
ST3GAL6	ST3 beta-galactoside alpha-2,3-sialyltransferase 6	ChEST 652m23	Gga.43077	Y	
ST6GAL1	ST6 beta-galactosamide alpha-2,6-sialyltranferase 1	ChEST 663h10	Gga.1148	Y	Y
ST6GALNAC2	ST6 (alpha-N-acetyl-neuraminyl-2,3-beta-galactosyl-1,3)-N-acetylgalactosaminide alpha-2,6-sialyltransferase 2	ChEST 674i15	Gga.39684		
ST6GALNAC3	ST6 (alpha-N-acetyl-neuraminyl-2,3-beta-galactosyl-1,3)-N-acetylgalactosaminide alpha-2,6-sialyltransferase 3	ChEST 651o11	Gga.15311		

ST8SIA3	ST8 alpha-N-acetyl-neuraminide alpha-2,8-sialyltransferase 3	ChEST 387f22	Gga.19311	Y	
ST8SIA4	ST8 alpha-N-acetyl-neuraminide alpha-2,8-sialyltransferase 4	ChEST 697d7	Gga.894	Y	
STAT3	Signal transducer and activator of transcription 3 (acute-phase response factor)	ChEST 603h18	Gga.32114	Y	
STK10	Serine/threonine kinase 10	ChEST 609h8	Gga.7203	Y	
STK17A	Serine/threonine kinase 17a	ChEST 647g17	Gga.20393		
STK17B	Serine/threonine kinase 17b	ChEST 603a17	Gga.9012	Y	
STK25	Serine/threonine kinase 25 (STE20 homolog, yeast)	ChEST 603l2	Gga.43174	Y	

STK31	Serine/threonine kinase 31	ChEST 713g4	Gga.32518	Y	Y
STRAP	Serine/threonine kinase receptor associated protein	ChEST 676b23	Gga.44357	Y	
STT3A	STT3, subunit of the oligosaccharyltransferase complEX, homolog A (S. cerevisiae)	ChEST 622l12	Gga.4288		
STYK1	Serine/threonine/tyrosine kinase 1	ChEST 605h19	Gga.30369	Y	Y
SUCLA2	Succinate-CoA ligase, ADP-forming, beta subunit	ChEST 648j18	Gga.22008	Y	
SUCLG1	Succinate-CoA ligase, GDP-forming, alpha subunit	ChEST 514f11	Gga.16559	Y	Y
SUCLG2	Succinate-CoA ligase, GDP-forming, beta subunit	ChEST 701b20	Gga.2557		

SULF2	Sulfatase 2	ChEST 605b19	Gga.12100	Y	
SULT1B1	Similar to sulfotransferase 1B	ChEST 592l3	Gga.8853	Y	Y
SULT1C3	Sulfotransferase family, cytosolic, 1C, member 3	ChEST 586f17	Gga.2667	Y	Y
SULT1E1	Sulfotransferase family 1E, estrogen-preferring, member 1	ChEST 648d16	Gga.5859	Y	
SULT4A1	Sulfotransferase family 4A, member 1	ChEST 670l23	Gga.22041	Y	
SULT6B1	Sulfotransferase family, cytosolic, 6B, member 1	ChEST 709a9	Gga.27805		
SUMF2	Sulfatase modifying factor 2	ChEST 665n1	Gga.12468		

SYDE2	Synapse defective 1, Rho GTPase, homolog 2 (C. elegans)	ChEST 609f10	Gga.28870		
SYK	Spleen tyrosine kinase	ChEST 697a4	Gga.22529	Y	Y
SYNGR3	Transcribed locus, strongly similar to NP_001008463.1 phosphatidylglycerophosphate synthase 1 [Gallus gallus]	ChEST 642m4	Gga.42500		
TALDO1	Transaldolase 1	ChEST 387m21	Gga.22033	Y	
TAOK1	TAO kinase 1	ChEST 601i15	Gga.44768	Y	Y
TAOK3	TAO kinase 3	ChEST 662j7	Gga.4360	Y	
TAP2	Transporter 2, ATP-binding cassette, sub-family B (MDR/TAP)	ChEST 619l22	Gga.2865	Y	

TARSL2	Threonyl-tRNA synthetase-like 2	ChEST 652c8	Gga.11029	Y	
TASP1	Taspase, threonine aspartase, 1	ChEST 715k12	Gga.14160	Y	
TAT	Tyrosine aminotransferase	ChEST 736g14	Gga.23758	Y	Y
TATDN1	TatD DNase domain containing 1	ChEST 564p9	Gga.39213	Y	
TCIRG1	T-cell, immune regulator 1, ATPase, H+ transporting, lysosomal V0 subunit A3	ChEST 675n14	Gga.4511	Y	
TDG	Thymine-DNA glycosylase	ChEST 600m20	Gga.3142	Y	Y
TDP1	Tyrosyl-DNA phosphodiesterase 1	ChEST 654c19	Gga.16007		

TDRD3	Transcribed locus, moderately similar to NP_569735.1 dermatan 4 sulfotransferase 1 [Homo sapiens]	ChEST 731k23	Gga.44573		
TEC	Tec protein tyrosine kinase	ChEST 630a14	Gga.2063		
TEK	TEK tyrosine kinase, endothelial (venous malformations, multiple cutaneous and mucosal)	ChEST 698n16	Gga.30770	Y	Y
TERT	Telomerase reverse transcriptase	ChEST 696f7	Gga.11755		
TGDS	TDP-glucose 4,6-dehydratase	ChEST 709j14	Gga.27361		
TGFB3	Transcribed locus, strongly similar to NP_055733.2 carbohydrate (chondroitin) synthase 1 [Homo sapiens]	ChEST 597o19	Gga.42151	Y	Y
TGFBR1	Transforming growth factor, beta receptor I (activin A receptor type II-like kinase, 53kDa)	ChEST 613o6	Gga.143		

TGM2	Transglutaminase 2 (C polypeptide, protein-glutamine-gamma-glutamyltransferase)	ChEST 586g15	Gga.31956	Y	Y
TGM3	Transglutaminase 3 (E polypeptide, protein-glutamine-gamma-glutamyltransferase)	ChEST 697i14	Gga.40927	Y	
TGM4	Transglutaminase 4 (prostate)	ChEST 619i17	Gga.7363	Y	
TGS1	Trimethylguanosine synthase homolog (S. cerevisiae)	ChEST 713h20	Gga.18138	Y	
THEM2	Thioesterase superfamily member 2	ChEST 612d10	Gga.12114	Y	Y
THEX1	Three prime histone mRNA EXonuclease 1	ChEST 646i13	Gga.2609	Y	
THG1L	TRNA-histidine guanylyltransferase 1-like (S. cerevisiae)	ChEST 670p21	Gga.22167	Y	

THNSL2	Threonine synthase-like 2 (S. cerevisiae)	ChEST 639b23	Gga.12047	Y	
TIMP2	TIMP metallopeptidase inhibitor 2	ChEST 619g13	Gga.2965		
TIMP3	TIMP metallopeptidase inhibitor 3 (Sorsby fundus dystrophy, pseudoinflammatory)	ChEST 685p19	Gga.2827	Y	
TKT	Transketolase (Wernicke-Korsakoff syndrome)	ChEST 563n11	Gga.21333	Y	
TLK2	Tousled-like kinase 2	ChEST 621a3	Gga.6272	Y	
TMLHE	Trimethyllysine hydroxylase, epsilon	ChEST 684c1	Gga.18010	Y	
TMPRSS2	Transmembrane protease, serine 2	ChEST 604o16	Gga.15567	Y	

TMPRSS6	Transmembrane protease, serine 6	ChEST 736k24	Gga.43067	Y	Y
TMPRSS7	Transmembrane protease, serine 7	ChEST 639b19	Gga.29761	Y	
TNIK	TRAF2 and NCK interacting kinase	ChEST 635g19	Gga.26472	Y	
TNKS1BP1	Tankyrase 1 binding protein 1, 182kDa	ChEST 564i12	Gga.31558		
TOP1	Topoisomerase (DNA) I	ChEST 677n13	Gga.42138	Y	
TOP2A	Topoisomerase (DNA) II alpha 170kDa	ChEST 650i10	Gga.2010	Y	
TOP2B	Topoisomerase (DNA) II beta 180kDa	ChEST 387o14	Gga.34448	Y	Y

TOP3A	Topoisomerase (DNA) III alpha	ChEST 533p13	Gga.32079	Y	
TOP3B	Topoisomerase (DNA) III beta	ChEST 689m16	Gga.16362	Y	
TOPBP1	Topoisomerase (DNA) II binding protein 1	ChEST 649b21	Gga.11098	Y	
TOPORS	Topoisomerase I binding, arginine/serine-rich	ChEST 685a10	Gga.27232		
TPI1	Triosephosphate isomerase 1	ChEST 571i19	Gga.4148	Y	Y
TPK1	Thiamin pyrophosphokinase 1	ChEST 637d12	Gga.12854	Y	
TPMT	Thiopurine S-methyltransferase	ChEST 637e17	Gga.16729	Y	

TPST1	Tyrosylprotein sulfotransferase 1	ChEST 564m22	Gga.12045		
TPST2	Tyrosylprotein sulfotransferase 2	ChEST 613g5	Gga.6013		
TPTE2	Transmembrane phosphoinositide 3-phosphatase and tensin homolog 2	ChEST 659h14	Gga.16817	Y	
TRDMT1	TRNA aspartic acid methyltransferase 1	ChEST 620h7	Gga.21361		
TRIM24	Transcribed locus, moderately similar to NP_033869.1 branched chain ketoacid dehydrogenase kinase [Mus musculus]	ChEST 675b10	Gga.30692		
TRIT1	TRNA isopentenyltransferase 1	ChEST 645b7	Gga.35356	Y	Y
TRMT12	TRNA methyltransferase 12 homolog (S. cerevisiae)	ChEST 597b14	Gga.15389		

TRMT5	TRM5 tRNA methyltransferase 5 homolog (S. cerevisiae)	ChEST 602c9	Gga.17444	Y	
TRPS1	Transcribed locus, weakly similar to NP_112217.2 ADAM metallopeptidase with thrombospondin type 1 motif, 12 preproprotein [Homo sapiens]	ChEST 657p12	Gga.25818	Y	
TRSPAP1	TRNA selenocysteine associated protein 1	ChEST 707o12	Gga.31065	Y	
TRUB1	Transcribed locus, strongly similar to XP_421776.1 PREDICTED: similar to TruB pseudouridine synthase-like protein 1 [Gallus gallus]	ChEST 683i19	Gga.16428	Y	Y
TRUB1	TruB pseudouridine (psi) synthase homolog 1 (E. coli)	ChEST 642n17	Gga.16428	Y	Y
TRUB2	TruB pseudouridine (psi) synthase homolog 2 (E. coli)	ChEST 719l22	Gga.9990	Y	
TSEN54	TRNA splicing endonuclease 54 homolog (S. cerevisiae)	ChEST 619f13	Gga.26678	Y	Y

TSGA10	Transcribed locus, moderately similar to XP_001232196.1 PREDICTED: similar to rac GTPase activating protein; GAB-associated CDC42 [Gallus gallus]	ChEST 721k2	Gga.15505	Y	
TST	Thiosulfate sulfurtransferase (rhodanese)	ChEST 520i2	Gga.5294		
TTL	Tubulin tyrosine ligase	ChEST 645g14	Gga.14457	Y	
TTLL1	Tubulin tyrosine ligase-like family, member 1	ChEST 571b9	Gga.27485	Y	
TTLL11	Tubulin tyrosine ligase-like family, member 11	ChEST 711p17	Gga.11675	Y	
TTLL12	Tubulin tyrosine ligase-like family, member 12	ChEST 640k22	Gga.21097	Y	
TTLL4	Tubulin tyrosine ligase-like family, member 4	ChEST 659j7	Gga.35832	Y	

TTLL5	Tubulin tyrosine ligase-like family, member 5	ChEST 672d12	Gga.16381	Y	
TWSG1	Transcribed locus, strongly similar to NP_001026027.1 solute carrier organic anion transporter family member 4A1 [Gallus gallus]	ChEST 728m21	Gga.43836		
TXK	TXK tyrosine kinase	ChEST 609h1	Gga.30009	Y	
TXN	Thioredoxin	ChEST 571i8	Gga.8723		
TXN2	Thioredoxin 2	ChEST 563j10	Gga.8941	Y	Y
TXN2	Thioredoxin 2	ChEST 563j10	Gga.8941		
TXNDC10	Thioredoxin domain containing 10	ChEST 514k10	Gga.15128	Y	

TXNDC11	Thioredoxin domain containing 11	ChEST 648p4	Gga.38492	Y	
TXNDC12	Thioredoxin domain containing 12 (endoplasmic reticulum)	ChEST 601n13	Gga.1223	Y	
TXNDC12	Thioredoxin domain containing 12 (endoplasmic reticulum)	ChEST 601n13	Gga.1223		
TXNDC13	Thioredoxin domain containing 13	ChEST 374c21	Gga.44456	Y	
TXNDC14	Thioredoxin domain containing 14	ChEST 514f15	Gga.34886	Y	Y
TXNDC15	Thioredoxin domain containing 15	ChEST 586b21	Gga.11431		
TXNDC16	Thioredoxin domain containing 16	ChEST 613p24	Gga.35236		

TXNDC17	Thioredoxin domain containing 17	ChEST 602n13	Gga.4867	Y	
TXNDC4	Thioredoxin domain containing 4 (endoplasmic reticulum)	ChEST 601b3	Gga.22091	Y	Y
TXNDC5	Thioredoxin domain containing 5	ChEST 671e10	Gga.3319	Y	
TXNDC9	Thioredoxin domain containing 9	ChEST 613b8	Gga.5770	Y	
TXNL1	Thioredoxin-like 1	ChEST 612c5	Gga.2083		
TXNL4A	Thioredoxin-like 4A	ChEST 619h13	Gga.7723	Y	
TXNL4B	Thioredoxin-like 4B	ChEST 601o15	Gga.9828	Y	

TXNL4B	Thioredoxin-like 4B	ChEST 601o15	Gga.9828		
TXNRD3	Thioredoxin reductase 3	ChEST 647n4	Gga.8908		
TYMS	Thymidylate synthetase	ChEST 672h23	Gga.12234		
TYRO3	TYRO3 protein tyrosine kinase	ChEST 613j24	Gga.4266		
UAP1L1	Similar to UDP-N-acteylglucosamine pyrophosphorylase 1-like 1	ChEST 712b9	Gga.30676	Y	
UBE3A	Ubiquitin protein ligase E3A (human papilloma virus E6-associated protein, Angelman syndrome)	ChEST 387a21	Gga.17148	Y	
UBE3B	Ubiquitin protein ligase E3B	ChEST 631h2	Gga.10374	Y	Y

UBE3C	Ubiquitin protein ligase E3C	ChEST 639k19	Gga.10028	Y	Y
UBIAD1	UbiA prenyltransferase domain containing 1	ChEST 600a5	Gga.6000	Y	Y
UBLCP1	Ubiquitin-like domain containing CTD phosphatase 1	ChEST 731c10	Gga.22390	Y	
UBR1	Ubiquitin protein ligase E3 component n-recognin 1	ChEST 514k8	Gga.24188	Y	Y
UBR3	Ubiquitin protein ligase E3 component n-recognin 3	ChEST 699k8	Gga.23653	Y	
UBR4	Ubiquitin protein ligase E3 component n-recognin 4	ChEST 653j9	Gga.14530	Y	
UCHL1	Ubiquitin carboxyl-terminal esterase L1 (ubiquitin thiolesterase)	ChEST 563j21	Gga.12837		

UCHL3	Ubiquitin carboxyl-terminal esterase L3 (ubiquitin thiolesterase)	ChEST 387f2	Gga.43413	Y	
UCHL5	Ubiquitin carboxyl-terminal hydrolase L5	ChEST 374d22	Gga.3929	Y	
UCK1	Uridine-cytidine kinase 1	ChEST 631o8	Gga.34733	Y	
UCK2	Uridine-cytidine kinase 2	ChEST 637a23	Gga.34527		
UEVLD	UEV and lactate/malate dehyrogenase domains	ChEST 374h22	Gga.9332		
UGCG	UDP-glucose ceramide glucosyltransferase	ChEST 627f8	Gga.35424	Y	
UGCGL1	UDP-glucose ceramide glucosyltransferase-like 1	ChEST 655a17	Gga.19243	Y	

UGDH	UDP-glucose dehydrogenase	ChEST 374e11	Gga.8846	Y	Y
UGP2	UDP-glucose pyrophosphorylase 2	ChEST 657f24	Gga.2423	Y	
UGT1A1	Transcribed locus, strongly similar to XP_542973.2 PREDICTED: similar to Matrix metalloproteinase-24 precursor (MMP-24) (Membrane-type matrix metalloproteinase 5) (MT-MMP 5)	ChEST 717b24	Gga.30084	Y	
UGT2A3	UDP glucuronosyltransferase 2 family, polypeptide A3	ChEST 604g18	Gga.29991	Y	
UGT8	UDP glycosyltransferase 8 (UDP-galactose ceramide galactosyltransferase)	ChEST 677m20	Gga.183	Y	
ULK4	Unc-51-like kinase 4 (C. elegans)	ChEST 672k4	Gga.21957		
UMPS	Uridine monophosphate synthetase (orotate phosphoribosyl transferase and orotidine-5'-decarboxylase)	ChEST 387d13	Gga.9322	Y	

UNG	Uracil-DNA glycosylase	ChEST 649b1	Gga.42288	Y	
UPB1	Ureidopropionase, beta	ChEST 621k12	Gga.13071		
UPP2	Uridine phosphorylase 2	ChEST 659d20	Gga.10346	Y	Y
UQCC	Ubiquinol-cytochrome c reductase complEX chaperone, CBP3 homolog (yeast)	ChEST 622m6	Gga.19121	Y	Y
UQCRB	Ubiquinol-cytochrome c reductase binding protein	ChEST 672f15	Gga.9987		
UQCRC1	Similar to ubiquinol--cytochrome c reductase	ChEST 650d14	Gga.4338	Y	Y
UQCRC2	Ubiquinol-cytochrome c reductase core protein II	ChEST 387b12	Gga.1021	Y	

UQCRFSL1	Ubiquinol-cytochrome c reductase, Rieske iron-sulfur polypeptide-like 1	ChEST 609k4	Gga.5791	Y	
UQCRQ	Ubiquinol-cytochrome c reductase, complEX III subunit VII, 9.5kDa	ChEST 601g4	Gga.2751	Y	
UROD	Uroporphyrinogen decarboxylase	ChEST 619n9	Gga.31285	Y	Y
UROS	Uroporphyrinogen III synthase (congenital erythropoietic porphyria)	ChEST 628g14	Gga.5964	Y	
USP1	Ubiquitin specific peptidase 1	ChEST 619j6	Gga.22540	Y	
USP10	Ubiquitin specific peptidase 10	ChEST 681i1	Gga.3193	Y	
USP13	Ubiquitin specific peptidase 13 (isopeptidase T-3)	ChEST 644n10	Gga.27614	Y	Y

USP14	Ubiquitin specific peptidase 14 (tRNA-guanine transglycosylase)	ChEST 659h20	Gga.16427	Y	
USP16	Ubiquitin specific peptidase 16	ChEST 617m4	Gga.38263	Y	Y
USP18	Ubiquitin specific peptidase 18	ChEST 724l4	Gga.1111		
USP2	Ubiquitin specific peptidase 2	ChEST 563l3	Gga.454	Y	
USP20	Ubiquitin specific peptidase 20	ChEST 593o11	Gga.31346	Y	
USP22	Ubiquitin specific peptidase 22	ChEST 625a6	Gga.39717	Y	
USP24	Ubiquitin specific peptidase 24	ChEST 514m9	Gga.10597	Y	Y

USP25	Ubiquitin specific peptidase 25	ChEST 564p5	Gga.16908	Y	
USP28	Ubiquitin specific peptidase 28	ChEST 684h1	Gga.22397	Y	
USP3	Ubiquitin specific peptidase 3	ChEST 604e14	Gga.39245	Y	
USP30	Ubiquitin specific peptidase 30	ChEST 717m24	Gga.17448	Y	
USP32	Ubiquitin specific peptidase 32	ChEST 592c18	Gga.8855		
USP33	Ubiquitin specific peptidase 33	ChEST 564l12	Gga.26668	Y	
USP34	Ubiquitin specific peptidase 34	ChEST 608m21	Gga.6715	Y	Y

USP37	Ubiquitin specific peptidase 37	ChEST 723d24	Gga.35854		
USP38	Ubiquitin specific peptidase 38	ChEST 602p6	Gga.36386	Y	
USP4	Ubiquitin specific peptidase 4 (proto-oncogene)	ChEST 799d23	Gga.41912	Y	
USP42	Ubiquitin specific peptidase 42	ChEST 824i7	Gga.30554	Y	
USP45	Ubiquitin specific peptidase 45	ChEST 691b15	Gga.22454		
USP47	Ubiquitin specific peptidase 47	ChEST 601e6	Gga.5374	Y	
USP48	Ubiquitin specific peptidase 48	ChEST 617c19	Gga.42306	Y	

USP5	Ubiquitin specific peptidase 5 (isopeptidase T)	ChEST 611b14	Gga.5788		
USP54	Ubiquitin specific peptidase 54	ChEST 600k8	Gga.10065	Y	
USP7	Ubiquitin specific peptidase 7 (herpes virus-associated)	ChEST 593a2	Gga.9341	Y	
USP8	Ubiquitin specific peptidase 8	ChEST 387a2	Gga.9603	Y	
USP9X	Ubiquitin specific peptidase 9, X-linked	ChEST 611f7	Gga.18949		
USPL1	Ubiquitin specific peptidase like 1	ChEST 617b1	Gga.4471	Y	
UST	Uronyl-2-sulfotransferase	ChEST 696h19	Gga.31791	Y	Y

UTP14A	Transcribed locus, strongly similar to NP_005843.2 chromodomain helicase DNA binding protein 3 isoform 2 [Homo sapiens]	ChEST 658n19	Gga.42620		
UXS1	UDP-glucuronate decarboxylase 1	ChEST 571a17	Gga.21981	Y	
VKORC1L1	Vitamin K epoxide reductase complEX, subunit 1-like 1	ChEST 619b21	Gga.22912	Y	
VPS29	Transcribed locus, moderately similar to NP_001025778.1 arginyl-tRNA synthetase [Gallus gallus]	ChEST 648i12	Gga.42541	Y	
VRK3	Vaccinia related kinase 3	ChEST 635p18	Gga.42400	Y	
WASF3	Transcribed locus, moderately similar to NP_766280.1 bile acid beta-glucosidase [Mus musculus]	ChEST 605f13	Gga.42756		
WDR25	Transcribed locus, strongly similar to NP_001034405.1 glucuronidase, beta [Gallus gallus]	ChEST 627c4	Gga.26823	Y	

WNK2	WNK lysine deficient protein kinase 2	ChEST 743e2	Gga.26018	Y	Y
WRNIP1	Werner helicase interacting protein 1	ChEST 645l18	Gga.8370	Y	
WWP1	WW domain containing E3 ubiquitin protein ligase 1	ChEST 571h7	Gga.4998	Y	Y
WWP2	WW domain containing E3 ubiquitin protein ligase 2	ChEST 656i24	Gga.16253	Y	Y
XAB1	XPA binding protein 1, GTPase	ChEST 685p18	Gga.13464	Y	
XDH	Xanthine dehydrogenase	ChEST 710e2	Gga.43173	Y	Y
XPNPEP1	X-prolyl aminopeptidase (aminopeptidase P) 1, soluble	ChEST 601p12	Gga.27228	Y	

XPNPEP3	X-prolyl aminopeptidase (aminopeptidase P) 3, putative	ChEST 631i18	Gga.11722	Y	
XRN1	5'-3' EXoribonuclease 1	ChEST 638n14	Gga.23111	Y	
XRN2	5'-3' EXoribonuclease 2	ChEST 620m13	Gga.8002	Y	
XYLB	Xylulokinase homolog (H. influenzae)	ChEST 638i23	Gga.10481	Y	
YARS	Tyrosyl-tRNA synthetase	ChEST 563c22	Gga.4637		
YSK4	Transcribed locus, moderately similar to NP_035075.1 neutral sphingomyelinase (N-SMase) activation associated factor [Mus musculus]	ChEST 533g3	Gga.44398	Y	Y
YWHAB	Tyrosine 3-monooxygenase/tryptophan 5-monooxygenase activation protein, beta polypeptide	ChEST 608d23	Gga.34332		

YWHAE	Tyrosine 3-monooxygenase/tryptophan 5-monooxygenase activation protein, epsilon polypeptide	ChEST 650h8	Gga.4550	Y	Y
YWHAH	Tyrosine 3-monooxygenase/tryptophan 5-monooxygenase activation protein, eta polypeptide	ChEST 514d6	Gga.1521	Y	Y
YWHAQ	Tyrosine 3-monooxygenase/tryptophan 5-monooxygenase activation protein, theta polypeptide	ChEST 608l16	Gga.8471	Y	Y
ZADH1	Zinc binding alcohol dehydrogenase, domain containing 1	ChEST 593e16	Gga.16852	Y	
ZADH2	Zinc binding alcohol dehydrogenase, domain containing 2	ChEST 533b17	Gga.13394	Y	
ZAK	Sterile alpha motif and leucine zipper containing kinase AZK	ChEST 640g11	Gga.11492	Y	
ZDHHC16	Transcribed locus, moderately similar to NP_006832.1 solute carrier family 38, member 3 [Homo sapiens]	ChEST 729l10	Gga.28407		

ZMPSTE24	Zinc metallopeptidase (STE24 homolog, S. cerevisiae)	ChEST 625j11	Gga.31086	Y	
ZNF259	Transcribed locus, moderately similar to NP_001011441.1 phosphoribosylglycinamide formyltransferase, phosphoribosylglycinamide synthetase,	ChEST 605d16	Gga.42373	Y	
ZNF592	Transcribed locus, moderately similar to XP_001236566.1 PREDICTED: similar to DNA primase (subunit p48), partial [Gallus gallus]	ChEST 631p13	Gga.44191		
